# Unique expression, processing regulation, and regulatory network of peach (*Prunus persica*) miRNAs

**DOI:** 10.1186/1471-2229-12-149

**Published:** 2012-08-21

**Authors:** Hong Zhu, Rui Xia, Bingyu Zhao, Yong-qiang An, Chris D Dardick, Ann M Callahan, Zongrang Liu

**Affiliations:** 1 Department of Horticulture, Virginia Polytechnic Institute and State University, Blacksburg, VA, 24061, USA; 2 Alson H. Smith Agricultural Research and Extension Center, Virginia Polytechnic Institute and State University, Winchester, VA, 22602, USA; 3 Appalachian Fruit Research Station, Agricultural Research Service, United States Department of Agriculture, Kearneysville, WV, 25430, USA; 4 USDA-ARS, Plant Genetic Research, Danforth Plant Science Center, 975 N. Warson Road, St. Louis, MO, 63132, USA

**Keywords:** miRNA, Deep sequencing, *Prunus persica*, Pre-miRNA processing, *Trans*-acting siRNA, MYB

## Abstract

**Background:**

MicroRNAs (miRNAs) have recently emerged as important gene regulators in plants. MiRNAs and their targets have been extensively studied in *Arabidopsis* and rice. However, relatively little is known about the characterization of miRNAs and their target genes in peach (*Prunus persica*), which is a complex crop with unique developmental programs.

**Results:**

We performed small RNA deep sequencing and identified 47 peach-specific and 47 known miRNAs or families with distinct expression patterns. Together, the identified miRNAs targeted 80 genes, many of which have not been reported previously. Like the model plant systems, peach has two of the three conserved *trans*-acting siRNA biogenesis pathways with similar mechanistic features and target specificity. Unique to peach, three of the miRNAs collectively target 49 *MYB*s, 19 of which are known to regulate phenylpropanoid metabolism, a key pathway associated with stone hardening and fruit color development, highlighting a critical role of miRNAs in the regulation of peach fruit development and ripening. We also found that the majority of the miRNAs were differentially regulated in different tissues, in part due to differential processing of miRNA precursors. Up to 16% of the peach-specific miRNAs were differentially processed from their precursors in a tissue specific fashion, which has been rarely observed in plant cells. The miRNA precursor processing activity appeared not to be coupled with its transcriptional activity but rather acted independently in peach.

**Conclusions:**

Collectively, the data characterizes the unique expression pattern and processing regulation of peach miRNAs and demonstrates the presence of a complex, multi-level miRNA regulatory network capable of targeting a wide variety of biological functions, including phenylpropanoid pathways which play a multifaceted spatial-temporal role in peach fruit development.

## Background

There are many mechanisms by which plants regulate gene expression to ensure normal development and appropriate responses to both biotic and abiotic signals. One regulatory mechanism involves endogenous small RNA (sRNA) molecules, 20~24-nt in length [1,2], which act by silencing gene expression. In plants, sRNAs have been classified based on their biogenesis, including microRNAs (miRNAs), heterochromatic siRNAs (hc-siRNAs), *trans*-acting siRNAs (tasiRNAs) and natural antisense siRNAs (nat-siRNAs) [1,3-6]. TasiRNA biogenesis from *TAS* loci depends on miRNA-directed cleavage of their transcripts [4,7,8] and three tasiRNA pathways have been characterized in *Arabidopsis*[7,9]. Although miRNAs only constitute a small fraction in the sRNA population [10,11], the miRNA-guided post-transcriptional gene regulation is one of the most conserved and well-characterized gene regulatory mechanisms [6,10,12]. Increasing evidence shows that miRNAs negatively regulate their target genes, which function in a wide range of biological processes, including organogenesis, signal transduction and stress responses [13,14].

MiRNAs are derived from a precursor sequence of ~70 bases that usually forms a 21 bp duplex with a conserved stem and variable loops which is excised to produce the mature miRNA. The 21 bp sequence matches one or more target sequences for cleavage [10,13]. MiRNAs were initially identified by direct cloning with bioinformatic prediction or Sanger sequencing of relatively small-sized cDNA libraries [15,16]. The application of deep sequencing has greatly facilitated the pace of miRNA identification in plants. In addition to *Arabidopsis* and rice [9,17], miRNAs have been identified in many other plant species, including poplar [18], tomato [19], maize [20], grape [21], peanut [22] and soybean [23]. Comparative analysis reveals that some of the miRNA families are highly conserved among all plant species while others have diverged and evolved, generating abundant family- and species-specific miRNAs [10,24,25]. These dynamic and evolving miRNAs could serve as a driving force for the selection of improved and novel traits in plants.

Peach (*Prunus persica*) is a model species for genomics studies in the Rosaceae family, which includes a number of economically important fruit tree species such as apple, cherry and plum. It has a relatively small and well annotated genome (~230 Mb), diploid, and there are numerous EST sequences. Peach have a number of unique biological facets not commonly found in model organisms such as a 3–5 year juvenility period before the trees flower and fruit [26]. In addition, as temperate zone plants, the reproductive cycle is one year with flower buds initiating during the previous summer. They enter dormancy triggered by cold weather and/or short photoperiod in the fall, and continue developing when released by the seasonal accumulation of chilling stimulus to bloom in the spring [27,28]. Another distinct feature of peach fruit relative to *Arabidopsis* is the formation of fleshy fruit with hardened endocarp or stone surrounding the seed. Stone formation is closely coordinated with fruit development [29,30]. Lastly, peach can be productive for several decades under changing conditions in the orchard. Conceivably, all these developmental programs require an array of sophisticated regulatory networks, involving numerous players presumably including miRNAs.

Recently, Zhang *et al*. [31] initiated the exploration of miRNAs in peach by computationally identifying 22 miRNAs and experimentally verifying miRNAs for seven conserved miRNA families. We wanted to know if peach has evolved novel miRNAs to correspond with its potentially novel development and growth, and what their targets are. To address this, we performed a comprehensive analysis of peach miRNAs from different tissues by deep sequencing, computational prediction, and molecular approaches. We were able to identify novel and conserved peach miRNAs as well as their targets. A majority of the miRNAs showed tissue-specific expression and 16% of them were found to be regulated at the post-transcriptional level. In addition, peach conserves two of the three *trans*-acting siRNA pathways and we identified additional protein-coding transcripts as tasiRNA biogenesis loci.

## Results

### sRNA population in peach

**Figure 1:**
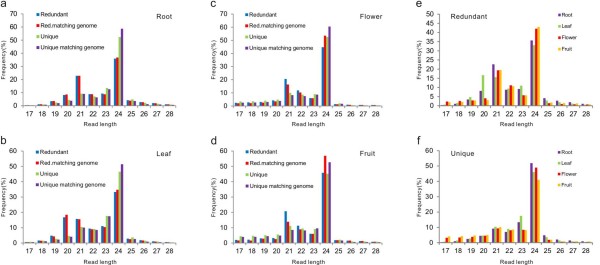
**Length distribution of redundant and unique sRNA sequences.** (**a**-**d**) The percentage of each size sRNA sequence (17~28-nt) in root, leaf, flower, and fruit is similar. The data is further grouped by whether the sequences are redundant or unique and by whether they align to the peach genome. (**e**-**f**) The data from (a-d) is summarized to represent the length distribution of redundant and unique sRNAs in root, leaf, flower, and fruit. In all cases, the 24-nt is the predominant sRNA species and the 21-nt is the next most abundant.

Four cDNA libraries made from peach root, leaf, flower and mixed fruit sRNAs yielded 50 million high quality reads. Among 10 million unique reads, ranging from 15- to 31-nt, 70% were perfectly matched to at least one locus in the peach genome (Peach Genome V1.0 scaffolds, http://www.rosaceae.org/peach/genome). These reads were used for further analysis (Additional file 1: Table S1). The 20~24-nt sRNAs constituted over 90% of the identified peach sRNAs, and the 24-nt class of sRNAs was the most abundant class in all tissues (Figure 1a-d). The redundant 24-nt sRNAs were more abundant in flower and mixed fruit than in root and leaf, and the redundant 21-nt sRNAs were more highly expressed in root than other tissues (Figure 1e). Notably, the expression of the unique 24-nt sRNAs was much higher than the 21-nt class in all tissues, especially in root (Figure 1f).

### Known miRNA families and their expression in peach

To identify known miRNA families in peach, we blasted all sRNA sequences against miRBase (release 18). A total of 258 unique sRNA sequences (20~22-nt) were identified belonging to 23 miRNA families that are conserved in both angiosperms and coniferophyta lineages [25] and referred to as conserved miRNAs in this study. These conserved miRNAs varied greatly in expression levels (Additional file 2: Table S2). In addition, most conserved miRNA families showed differential expression among root, leaf, flower and mixed fruit tissues. An additional eighty-three miRNA sequences belonged to 24 miRNA families (Additional file 3: Table S3) that have been identified and reported in at least one plant species or family [10]. These are referred to as less-conserved miRNAs in this work. A canonical predicted stem-loop structure could be identified in seven of the 24 less-conserved miRNA families (Additional file 4: Table S4, Additional file 5: Figure S1). Overall, all the less-conserved miRNAs displayed relatively low expression levels compared to the conserved miRNAs except for miR535, and they, like the conserved miRNAs, displayed differential expression among tissues (Additional file 3: Table S3).

**Figure 2:**
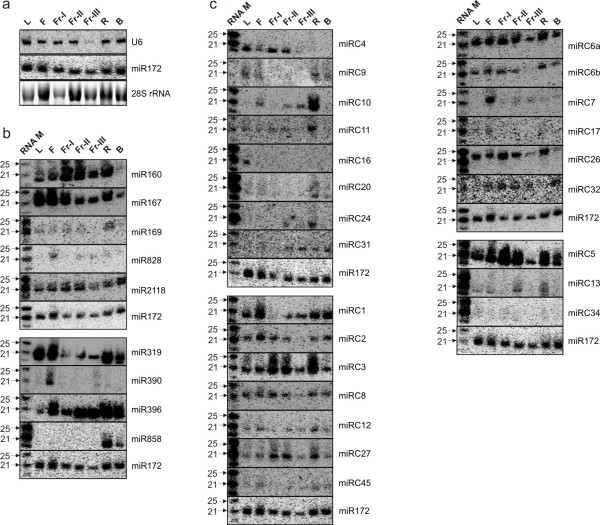
**RNA blot analysis of miRNA expression.** (**a**) The expression profiles of three molecules for normalizing gel loading were used to determine the one most uniformly expressed in the seven different RNA samples. MiR172 was chosen as the loading control throughout all RNA blots in this study instead of peach U6 because the U6 proved to be expressed at a substantially lower level in the ripest fruit tissue in these experiments. (**b**-**c**) The expression of selected previously-known and peach-specific miRNAs in different peach tissues. All hybridization results from the same membrane are grouped. 25 μg of total RNA isolated from each tissue was separated, transferred to nylon membranes and hybridized using γ^32^P-labeled oligo probe complimentary to RNA marker sequence and along with the probe to the indicated miRNA or gene sequence. For all the blots shown, L, leaf; F, flower; Fr-I, fruit at 19 Day After Bloom (DAB); Fr-II, fruit at 40 DAB; Fr-III, fruit at 82 DAB; R, root; B, bark.

A random subset of the conserved and less-conserved miRNAs was analyzed with RNA blot to validate the expression data from the deep sequencing, utilizing miR172 as a standard (Figure 2a). The tissue-specific expression patterns were presented for the conserved miRNAs, miR160, miR167, miR169, miR319, miR390 and miR396, and the less-conserved miRNAs, miR828, miR858 and miR2118 (Figure 2b).

### Peach-specific miRNAs

After excluding sRNA reads homologous to known miRNAs and other non-coding RNAs, the remaining 20~22-nt sRNA reads were selected for secondary structure prediction. Those with canonical stem-loop structures and sequence specificity (>75% of the reads mapped to the unique locus) were considered as putative new miRNAs (Additional file 5: Figure S1). In total, 47 sRNAs from 134 loci were identified. 29 of these sRNAs qualified as novel peach miRNAs since their star stands were identified and the remaining 18 sRNAs without star strand detected were classified as candidate peach miRNAs (Table 1 and Additional file 6: Table S5). Collectively, we refer to them as peach-specific miRNAs as they at present have only been found in peach. Of the 47 peach-specific miRNAs, 29 were 21-nt, 17 were 22-nt, and one was 20-nt (Table 1). Thirty-eight of the miRNAs were transcribed from single loci and the remaining nine matched 2 to 15 loci. Approximately 67% of the peach-specific miRNAs mapped to the sense strand of genome scaffolds while only 33% of them mapped to the antisense strand (Additional file 6: Table S5).

**Table 1:** **Novel or candidate miRNAs identified from*P. persica*sRNA libraries**

**miRNA**	**miRNA sequence (5’-3’)**	**Length**	**Scaffold**^**a**^	**Match site**	**Strand**	**Normalized abundance (rpm**^**b**^**)**					miRNA* sequence (5’-3’)
						**Root**	**Leaf**	**Flower**	**Fruit**	**Total**	
**miRC1**	ACCUGGCUCUGAUACCAUAAC	21	Scaffold_3	8575480	+	1571	908	13139	7818	23436	CGUGGUAUCAGAGUCAUGUUA
**miRC2**	UGAAGGAAGAUUUGUGGAAAG	21	Scaffold_7	18921374	-	1573	3962	117	53	5705	UUCCACAGAUCUUUCCUCAUU
**miRC3**	CUUCCCAAACCUCCCAUUCCUA	22	Scaffold_1	29648613	+	40	33	1288	2804	4165	GGAAUGGGAGGAUUGGGAAAA
**miRC4**	UGAGCAAUGGCACACAGCCCU	21	Scaffold_3	2185580	+	0	0	1273	1837	3110	UUGUGCCAUUGCUCAAGC
**miRC5**	UUUCCGAAACCUCCCAUUCCAA	22	Scaffold_1	29646139	+	60	3	515	2080	2658	GGGUGAGAGGUUGCCGGAAAGA
**miRC6a**	UUAUACAAUGAAAUCACGGCCG	22	Scaffold_1	2254120	+	1286	309	249	328	2172	GCCGUGUUUCUUUGUAUAAAG
**miRC6b**	UUAUACAAUGAAAUCACGGUCG	22	Scaffold_1	2244523	+	97	15	58	10	180	CCGUGUUUCCUUGUAUAAAG
**miRC7**	UGGCACCAAUGAUACCAAGUUU	22	Scaffold_7	18801272	-	0	0	986	404	1390	ACUUGGUAUCUUGGUGCCGGU
**miRC8**	CAGGAAAGAAUGUGAUGAGUA	21	Scaffold_2	2899303	+	24	469	11	0	504	UUUGCUCGUCACAUUCUUUCC
**miRC9**	UCGCAGGAGAGAUGGCACUGUC	22	Scaffold_3	19986823	-	109	16	0	0	125	UGGUGUCAUCCCUCCUGUGACC
**miRC10**	CGAACUUAUUGCAACUAGCUU	21	Scaffold_4	6342241	+	3	19	0	56	78	GCUAGGUGCAACAAGUUCAAU
**miRC11**	GGAGCGACCUGGGAUCACAUG	21	Scaffold_4	23714205	+	12	14	1	22	49	UGUGUUCUCAGGUCGCCCCUG
**miRC12**	UCUGAGUCAGAUUACUGAAUA	21	Scaffold_6	8360495	+	10	32	1	0	43	UUCAGUAUUUUGACUCAGAA
**miRC13**	ACUCUCCCUCAAAGGCUUCUAG	22	Scaffold_5	11892663	+	7	14	4	12	37	CGAAGCCUUUGGGGAGAGUAA
**miRC14**	UAGAGAGAUGGUCAGCAAUGU	21	Scaffold_4	14225624	+	3	3	1	26	33	AUUGCUGAUCACCUCUCUAAU
**miRC15**	CCACAUUUAUAGAUUACCUUG	21	Scaffold_7	10402743	-	0	0	9	20	29	CAAGGUAGUUUAUAAAUGUGG
**miRC16**	UUCAAAGGGUACAUCCACAGU	21	Scaffold_2	18505840	+	0	5	8	9	22	CAACUGUGGACAUACCCUUUG
**miRC17**	UCUGUCGUAGGAGAGAUGGCGC	22	Scaffold_3	19984391	-	9	0	0	12	21	UCAUCUCUCCUCGACUGAA
**miRC18**	UCGUGGGGAGAGAUCUAAUCG	21	Scaffold_7	18333173	-	0	0	6	12	18	AUUAGACCUCUCCCGACGAAA
**miRC19**	CCUCCCAUGCCACGCAUUUCUA	22	Scaffold_8	10608407	-	0	0	11	6	17	GAGAUGGGUGGCUGGGAAGGA
**miRC20**	AUUUCGACUAAUAACACAAUG	21	Scaffold_7	1039113	+	3	0	2	11	16	UUGUGUUAUUGGCCGAAAAUAG
**miRC21**	AUAAUAAUGUCCGGAUGUCAA	21	Scaffold_6	19901315	+	0	11	0	0	11	GAUAUCCGCACAUUAUUAUUG
**miRC22**	CCCUUCCAGUAAGGCACCCCC	21	Scaffold_5	13061652	+	0	0	1	10	11	GGGUUCCUUGUUGGAAGGACU
**miRC23**	AUUUCAGCUAAGUUGAGUUGU	21	Scaffold_3	13433851	+	0	1	8	1	10	AAUCAACUCAGCUUAGCUGAACUG
**miRC24**	UCCCUCAAGGGCUCCCAAUAUU	22	Scaffold_3	9747805	-	2	0	0	8	10	UGUUGGGGGCUCUUUUG
**miRC25**	UCAAUUAGAAAAUGAUAAGUG	21	Scaffold_6	7122989	+	0	0	5	2	7	CUUGUUAUUUUUUAAUUGAUU
**miRC26**	UCCAACGAUGGGUGACCACAA	21	Scaffold_7	16562805	-	0	0	2	5	7	UUUGUGGUCAUUCACCGUUGGA
**miRC27**	UCCUGUGCGAACGUCCAGAAG	21	Scaffold_1	498129	+	3	4	0	0	7	UAACUUCCGAACGUCCGCAUA
**miRC28**	CUUGUUAUUUUUUAAUUGAUU	21	Scaffold_6	9969911	-	0	0	4	1	5	AACCAAUUAGAAAAUAACAAGUGG
**miRC29**	AAAGACUAAAAUACCCUUGA	20	Scaffold_5	6250039	-	8	6	45	1	60	None detected
**miRC30**	UACUUGACCCCACAACUGGUU	21	Scaffold_1	27613921	+	1	6	13	24	44	None detected
**miRC31**	UGGGCACGCCAGAAUAAAGCAA	22	Scaffold_7	13727378	+	12	27	0	0	39	None detected
**miRC32**	UAAGGUUGAGCCGGAAAUCGGA	22	Scaffold_6	8366918	+	2	3	2	19	26	None detected
**miRC33**	CUCUUAAUCGUUGGAUCAAAUU	22	Scaffold_5	10113319	-	0	0	0	22	22	None detected
**miRC34**	UGCUUGUUGAGAUGUGCGGUU	21	Scaffold_8	6836158	+	17	1	0	1	19	None detected
**miRC35**	UGUGUUAAUCGUAGAAAAUAU	21	Scaffold_1	27061839	+	6	2	8	1	17	None detected
**miRC36**	AAUGUCACCUCCCACACUCCU	21	Scaffold_4	23445268	+	0	0	0	16	16	None detected
**miRC37**	UGGACGUCUAGAAAAAUACGG	21	Scaffold_4	23035470	+	7	8	0	0	15	None detected
**miRC38**	UUAAGCCCAAGAAAGCCCGAC	21	Scaffold_4	23839384	+	0	0	0	14	14	None detected
**miRC39**	ACCUCUUAUAGAUAGUCCCCA	21	Scaffold_3	193497	+	0	0	0	12	12	None detected
**miRC40**	AGACAGGUUCUUUUAUCUCAUG	22	Scaffold_1	22425519	-	0	0	3	9	12	None detected
**miRC41**	UCGAUUUUAUGUUUUAAGUAUC	22	Scaffold_4	22126310	+	0	0	6	5	11	None detected
**miRC42**	UCUGACUUUUACCAGAAUCUGA	22	Scaffold_5	11020912	+	3	0	0	5	8	None detected
**miRC43**	CAUUAGAGCGGUGGUACACAA	21	Scaffold_1	30892686	+	1	4	2	0	7	None detected
**miRC44**	UGCCAAGAAAGAGUUGCCCUA	21	Scaffold_3	1327042	-	0	3	2	0	5	None detected
**miRC45**	ACCUCCUCAUUCUAACCCCUCA	22	Scaffold_1	29656462	-	0	0	0	4	4	None detected
**miRC46**	UGCAUGCACCUUGAUAGAUGU	21	Scaffold_5	17169215	-	0	0	0	4	4	None detected

^a^Only a single mapped scaffold is shown here. All matched scaffolds are included in Additional file 6: Table S5. ^b^rpm, reads per million.

The expression of all peach-specific miRNAs was analyzed and compared by both sRNA-seq and RNA blot analyses. In general, peach-specific miRNAs had low expression which varied in different tissues (Table 1 and Figure 2c). We detected signals for 24 of the 47 peach-specific miRNAs by RNA blot analyses in one or all seven tissues tested, and the hybridization signal intensity was, in general, correlated with the reads per million (rpm) values as demonstrated for miRC1 and miRC7 with some exceptions (Figure 2c).

### Detection of miRNA precursors and tissue- and development-specific differential processing in peach

**Figure 3:**
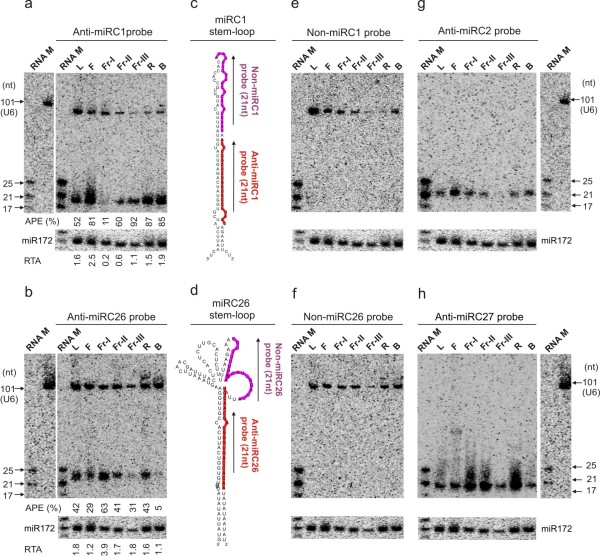
**Detection of differential miRNA processing and expression.** Two sets of membranes were prepared as described in Figure 2. Each membrane was sequentially probed with labeled RNA marker (RNA M) for size standards, probe complementary to a specific miRNA that shows incomplete processing, a 21-nt region immediately next to the specific miRNA sequence, a second miRNA that shows complete processing and then U6 as a standard. MiR172 was used as a loading control. (**a**-**b**) The expression of miRC1 and miRC26, respectively, showing incomplete processing. Based on the expression of the control RNA miR172, the processing efficiency is calculated and presented below the blots. APE, Arbitrary Processing Efficiency (%) = Small Fragment Intensity (SFI)*100/[Sum of Large Fragment Intensity (LFI) + SFI]. RTA, Relative Transcription Activity = (LFI + SFI)/Control RNA Fragment Intensity (CFI). (**c**-**d**) The diagram of the specifically probed sequence regions is shown. (**e**-**f**) The expression of precursors only is shown of miRC1 and miRC26, respectively. (**g**-**h**) The expression of miRC2 and miRC27 respectively is shown to demonstrate complete processing detected on the same blot. For all the blots shown, L, leaf; F, flower; Fr-I, fruit at 19 Day After Bloom (DAB); Fr-II, fruit at 40 DAB; Fr-III, fruit at 82 DAB; R, root; B, bark.

In plants, miRNA transcription and processing appear to be closely coupled as the transcripts are immediately processed [32,33]. This appeared to hold true for most of the peach miRNAs we analyzed. However, in the case of nine miRNAs, miRC1, miRC9, miRC11, miRC14, miRC16, miRC17, miRC26, miRC31 and miRC34 (Figure 3a,b and Additional file 7: Figure S2) two fragments were detected by RNA blot analyses; one corresponding to the expected 21~22-nt miRNA species and the other corresponding to RNA species ranging from 90 to 130-nt in size, which is the predicted size range of most miRNA precursors. To assess if these large fragment were miRNA precursors, we designed two 21-nt oligo probes (designated as Non-miRC1 and Non-miRC26) complementary only with non-miRNA sequences within stem loops of the selected miRC1 and miRC26 (Figure 3c,d). These probes should detect only the large fragment because they were not complementary with the miRNA sequences. Indeed, only the large fragment was detected with each of these probes (Figure 3e,f). Re-probing the same blot with another miRNA did not detect the large fragment, indicating that it was not an artifact (Figure 3g,h). Therefore, the detected large fragments likely represented miRNA transcript precursors.

A comparison between large and small fragment intensity among tissues revealed that their relative ratios varied among tissues. For example, miRC26 was more abundant than its precursor in young fruit (Fr-I) while the opposite was true in bark tissue (Figure 3b). These findings suggest that these miRNAs are differentially processed in different tissues. To compare miRNA processing efficiency, we quantified both small and large fragment signal intensity and calculated the percentage of the small fragment intensity relative to the total fragment intensity (APE, arbitrary processing efficiency), as listed in bottom panels of Figure 3a,b. The APE for both miRC1 and miRC26 showed substantial variations among tissues, as over 80% of transcript precursors were processed in flower (F), root (R) and bark (B) but only 11% was processed in young fruit (Fr-I) for miRC1 (Figure 3a). Similarly, 63% of miRC26 precursors was processed in young fruit (Fr-I) while as little as 5% was processed in bark tissue (Figure 3b). In addition to tissue-specific regulation, miRNA processing was also influenced by fruit development stage as the APE for miRC1 increased from 11% to 92% during development from young fruit (Fr-I) to mature fruit (Fr-III) (Figure 3a) while an opposite trend was observed for miRC26 (Figure 3b). Apparently, the expression of these peach miRNAs was subjected to differential processing regulation in a tissue-specific and development-dependent fashion.

MiRNAs in plants are primarily regulated at the transcriptional level. We quantified the changes in transcription of the specific miRNAs among tissues by relative transcription activity (RTA) of miRC1 and miRC26, in regard to miR172 as the loading control. Specifically, miRC1 had the highest RTA in flower (F) and the lowest RTA in young fruit (Fr-I), while miRC26 had the highest RTA in young fruit (Fr-I) and the lowest RTA in bark (B) (Figure 3a,b). MiRNA transcription and processing shared similar activity in some tissues but different in other tissues, indicating that the two regulatory mechanisms could be uncoupled.

### Identification of miRNA targets in peach

We identified 64 target genes for known miRNAs through sequencing of a peach degradome library and found that most of them were abundantly represented as conserved miRNA targets (Table 2). Thirty-five target genes were identified for 15 of the conserved miRNA families, and 29 target genes for the nine less-conserved miRNA families. The target transcripts were then classified into five categories (0–4) based on their abundance (Table 2 and Table 3) [34]. Ten targets for the conserved miRNAs fell into the category 0, the most abundant, and half of the targets fell into category 2. We could not identify any targets for some conserved miRNA families regardless of whether the expression level of the corresponding miRNAs was low or high, indicating there is no clear correlation between miRNA level and the efficiency of cleavage of its target transcript.

**Table 2:** Targets for known miRNA in peach identified by degradome sequencing

**miRNA**	**Target gene**^**a**^	**Align score**^**b**^	**Normalized reads at cleavage site (tpb)**^**c**^	**Category**^**d**^	**Target gene annotation**
**Conserved targets for conserved miRNAs**					
miR156	ppa006611m	3	150.2	3	Squamosa promoter-binding-like protein
miR156	ppa007056m	2	500.7	2	Squamosa promoter-binding-like protein
miR156	ppa021582m	2	1151.5	2	Squamosa promoter-binding-like protein
miR156	ppa024285m	3	3704.8	0	Squamosa promoter-binding-like protein
miR159	ppa003628m	3.5	2252.9	0	MYB transcription factor
miR160	ppa002082m	1	1301.7	2	Auxin response factor
miR160	ppa002710m	1	1251.6	2	Auxin response factor
miR164	ppa007653m	2.5	11214.7	0	NAC domain-containing protein
miR165	ppa001343m	2.5	2353.1	2	Homeobox-leucine zipper protein
miR165	ppa001378m	2.5	4856.3	0	Homeobox-leucine zipper protein
miR166	ppa001343m	3	2353.1	2	Homeobox-leucine zipper protein
miR166	ppa001378m	3	4856.3	0	Homeobox-leucine zipper protein
miR167	ppa001179m	4.5	1201.6	2	Auxin response factor
miR168	ppa000619m	4	275.4	2	Argonaute protein
miR168	ppa000900m	4	275.4	2	Argonaute protein
miR169	ppa006634m	3.5	1226.6	2	Nuclear transcription factor Y subunit A
miR390	AJ875750 (EST)				PpTAS3
miR393	ppa003344m	1	1401.8	2	Auxin signaling F-box protein
miR393	ppa003465m	2	550.7	3	Auxin signaling F-box protein
miR395	ppa002425m	2	600.8	2	Sulfate transmembrane transporter
miR396	ppa003017m	3.5	4005.2	0	Growth-regulating factor
miR396	ppa006912m	3	4956.5	2	Growth-regulating factor
miR396	ppa011917m	3.5	7960.4	0	Growth-regulating factor
miR396	ppa019623m	3	9312.2	0	Growth-regulating factor
miR396	ppa021277m	4	22179.0	0	Growth-regulating factor
miR396	ppa022199m	3	28086.7	0	Growth-regulating factor
miR396	ppa024293m	3	700.9	2	Growth-regulating factor
miR397	ppa003308m	4	550.7	2	Laccase
miR397	ppa003408m	3	300.4	3	Laccase
miR397	ppa003646m	1.5	9011.8	0	Laccase
miR397	ppa003714m	3	400.5	3	Laccase
miR397	ppa022440m	1.5	250.3	3	Laccase
miR408	ppa018507m	1	1051.4	2	Copper ion binding protein
miR408	ppa021383m	3	3104.1	2	Copper ion binding protein
Tas3-siRNA	ppa001557m	2	650.9	2	Auxin response factor
Tas3-siRNA	ppa001392m	1.5	22529.4	2	Auxin response factor
**Novel targets for conserved miRNAs**					
miR396	ppa003643m	4	400.5	3	Rho guanyl-nucleotide exchange factor
miR408	ppa004802m	3.5	500.7	3	Selenium-binding protein
miR408	ppa007350m	4	3404.4	2	Cyclin D3
**Targets for other known miRNAs**					
miR505	ppa012208m	4.5	275.4	2	ATP synthase
miR505	ppa012241m	4.5	275.4	2	ATP synthase
miR530	ppa004922m	4	50.1	4	ATP binding
miR828	ppa010908m	1	55247.2	0	MYB transcription factor
miR828	ppa016135m	3	2152.8	2	MYB transcription factor
miR828	ppa024533m	2	1852.4	2	MYB transcription factor
miR858	ppa005421m	4.5	851.1	2	3-ketoacyl-CoA thiolase
miR858	ppa006057m	4.5	851.1	2	3-ketoacyl-CoA thiolase
miR858	ppa006769m	4	150.2	3	MYB transcription factor
miR858	ppa009143m	4	150.2	3	MYB transcription factor
miR858	ppa010252m	4	3754.9	2	MYB transcription factor
miR858	ppa015883m	3	650.9	0	MYB transcription factor
miR858	ppa016135m	3.5	16481.5	2	MYB transcription factor
miR858	ppa016385m	4	1201.6	0	MYB transcription factor
miR858	ppa016708m	4.5	650.9	2	MYB transcription factor
miR858	ppa017136m	3.5	35.0	3	MYB transcription factor
miR858	ppa018561m	4	1201.6	2	MYB transcription factor
miR858	ppa019380m	4	35.0	1	MYB transcription factor
miR858	ppa022205m	4	4070.3	0	MYB transcription factor
miR858	ppa022431m	4.5	6959.1	2	MYB transcription factor
miR858	ppa022465m	3.5	16481.5	0	MYB transcription factor
miR858	ppa023768m	2.5	3704.8	2	MYB transcription factor
miR858	ppa023812m	3.5	4070.3	0	MYB transcription factor
miR858	ppa024074m	4.5	4070.3	0	MYB transcription factor
miR894	ppa005211m	4	300.4	3	Ankyrin repeat family protein
miR2478	ppa024560m	4.5	300.4	3	Disease resistance-responsive protein
miR2911	ppa004713m	4.5	2052.7	2	Vacuolar processing enzyme
miR2916	ppa008099m	4.5	200.3	2	Galacturonosyltransferase-like protein
miR4171-5	ppa010474m	4.5	650.9	2	C3HC4-type RING finger family protein
miR4171-5	ppa012554m	4	150.2	3	Universal stress protein (USP)

^a^ Target gene is the identified transcript from the peach transcriptome (http://www.rosaceae.org/node/35) or the EST sequence in the case of miR390.

^b^ Align score is calculated according to [7].

^c^ tpb, transcripts per billion, according to [21].

^d^ Category 0: >1 raw read at the position, abundance at position is equal to the maximum on the transcript, and there is only one maximum on the transcript; Category 1: >1 raw read at the position, abundance at position is equal to the maximum on the transcript, and there is more than one maximum position on the transcript; Category 2: >1 raw read at the position, abundance at position is less than the maximum but higher than the median for the transcript; Category 3: >1 raw read at the position, abundance at position is equal to or less than the median for the transcript; Category 4: Only 1 raw read at the position.

**Table 3:** Targets for peach-specific miRNAs identified by degradome sequencing

**miRNA**	**Target gene**^**a**^	**Align score**^**b**^	**Normalized reads at cleavage site (tpb)**^**c**^	**Category**^**d**^	**Target gene annotation**
miRC2	ppa010261m			5	751.0	2	Zinc finger protein
miRC3	ppb024266m			3.5	350.5	2	NBS-LRR class disease resistance protein
miRC4	ppa012465m			4.5	10263.4	2	Unknown protein
miRC5	ppb024266m			5	350.5	2	NBS-LRR class disease resistance protein
miRC6a	ppa019098m			4	40.1	2	Pentatricopeptide (PPR) repeat-containing protein
miRC6a	ppa020475m			1	5997.8	0	Pentatricopeptide (PPR) repeat-containing protein
miRC6a	ppa023796m			5	15.0	4	Pentatricopeptide (PPR) repeat-containing protein
miRC6b	ppa019098m			4.5	40.1	2	Pentatricopeptide (PPR) repeat-containing protein
miRC6b	ppa020475m			1.5	5997.8	0	Pentatricopeptide (PPR) repeat-containing protein
miRC12	ppa000294m			5	600.8	2	Protein kinase family protein
miRC12	ppa000823m			5	851.1	2	Translation initiation factor
miRC13	ppa008890m			5	2353.1	2	Esterase/lipase/thioesterase family protein
miRC13	ppa010397m			4.5	100.1	3	Allene-oxide cyclase
miRC16	ppa018545m			5	100.1	3	FAR1-related sequence 3; zinc ion binding
miRC16	ppa022612m			5	100.1	3	FAR1-related sequence 3; zinc ion binding
miRC29	ppa002618m			5	50.1	3	RNA binding / translation initiation factor
miRC29	ppa002620m			5	50.1	3	RNA binding / translation initiation factor
miRC30	ppa004763m			4.5	25.0	4	Catalase
miRC45	ppa002666m			5	1552.0	2	Vernalization independence; DNA binding

^a^ Target gene is the identified transcript from the peach transcriptome (http://www.rosaceae.org/node/35).

^b^ Align score is calculated according to [7].

^c^ tpb, transcripts per billion, according to [21].

^d^ Category 0: >1 raw read at the position, abundance at position is equal to the maximum on the transcript, and there is only one maximum on the transcript; Category 1: >1 raw read at the position, abundance at position is equal to the maximum on the transcript, and there is more than one maximum position on the transcript; Category 2: >1 raw read at the position, abundance at position is less than the maximum but higher than the median for the transcript; Category 3: >1 raw read at the position, abundance at position is equal to or less than the median for the transcript; Category 4: Only 1 raw read at the position.

A large number of the identified targets were members of transcription factor gene families, including SPL, MYB, ARF, NAC and GRF, while others were related to sRNA binding (AGO), auxin signaling (TIR/AFB), sulfate transport (AST) and redox reactions (LAC and ARPN) (Table 2). For a given miRNA with multiple conserved target transcripts, the frequency of each target in the degradome varied. More importantly, novel targets were also identified for at least two conserved miRNAs, i.e. miR396 and miR408. The miR396 target encodes a rho guanyl-nucleotide exchange factor and the two miR408 targets encode a selenium-binding protein and a D-type cyclin (Table 2). We also identified either single or multiple targets for the nine less-conserved miRNAs in peach (Table 2). Both miR828 and miR858 targeted MYB family genes. MiR828 could cleave three *MYB* genes while miR858 targeted 18, among which they shared one common target. In addition, miR858 was found to target two other genes both encoding peroxisomal 3-ketoacyl-CoA thiolases that have critical roles in fatty acid metabolism [35] (Table 2). Notably, miR858 had the most gene targets identified in this study.

A total of 16 targets were identified for 12 of the peach-specific miRNAs (Table 3). Among the identified targets, only one fell into category 0; eight into category 2 and seven into category 3 or 4 (Table 3). MiRC6a and miRC6b shared two of three gene targets identified while miRC3 and miRC5 targeted the same gene transcript. MiRC12 and miRC13 target different genes while miRC16 and miRC29 target two genes from the same gene family. The other four peach-specific miRNAs were found to target single genes. The identified 16 gene targets encode diverse proteins including zinc finger, NBS-LRR class disease resistance, PPR containing, protein kinase, FAR1-related, RNA binding, catalase and vernalization-related proteins (Table 3), suggesting that these peach-specific miRNAs are likely involved in regulation of a wide range of biological processes or metabolic pathways.

### *Trans*-acting siRNAs in peach

**Figure 4:**
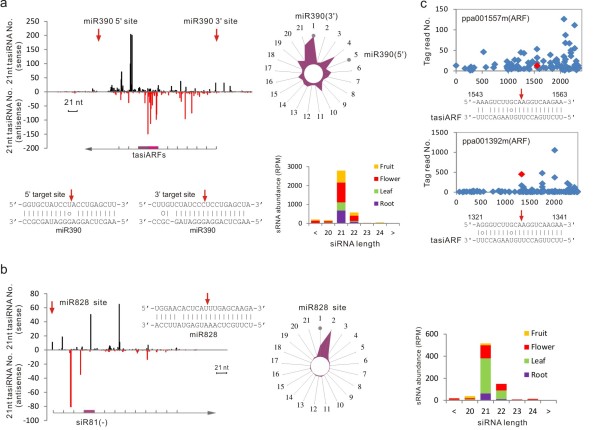
**Two peach trans-acting siRNA biogenesis pathways.** (**a**) The miR390-*TAS3* biogenesis pathway, showing the dual miR390 target sites on the *PpTAS3* transcript as denoted by red arrows. The number of sRNA sequences mapped along the *PpTAS3* transcript is plotted for sense (black line) and antisense (red line) strands, with the position of two conserved tasiARFs indicated below. The phasing radial graph is represented next to this. Each spoke of the radial graph represents 1 of the 21 phasing registers, with the total number of sRNAs mapping to that register plotted as distance from the center. Grey dots indicate the specific registers predicted by 21-nt processing from the 5’ and 3’ cleavage sites. The tissue-specific accumulation of the phased siRNAs in fruit, flower, leaf and root is shown below the phasing graph. (**b**) The miR828-*TAS4* biogenesis pathway, showing the miR828 cleavage site on the *PpTAS4* transcript as denoted with a red arrow at the 5' end. The phasing graph and tissue-specific accumulation of the phased siRNAs are shown, as illustrated in (a). (**c**) Degradome confirmation of the cleavage of two peach *ARF* transcripts by *TAS3*-derived tasiARFs using t-plot with the red diamond and arrow indicating cleavage sites.

In this study, we found that both miR390-*TAS3* and miR828-*TAS4* tasiRNA pathways are conserved in peach as evidenced by the identification of miR390 and miR828, *TAS3* and *TAS4* transcripts and the generation of phased 21-nt siRNAs along both *TAS3* and *TAS4* transcripts (Figure 4a,b). RNA blot analysis showed that both miR390 and miR828 had detectable expression in various peach tissues (Figure 2b). MiR390's cleavage target, a *TAS3* ortholog (EST: AJ875750, defined as *PpTAS3*) was identified in peach, and shared similar dual miR390 target sites with its *Arabidopsis* counterpart. Mapping of sRNA reads against *PpTAS3* defined a similar tasiRNA generation region and pattern between these dual target sites. Mostly 21-nt tasiRNAs were generated in the flower tissue (Figure 4a), which correlated with flower-specific expression of miR390 (Figure 2b). Of these siRNA populations, two siRNAs shared > 95% sequence identity with the characterized *Arabidopsis* tasiARFs, which were shown to target *AtARF2AtARF3*, and *AtARF4 *that negatively regulate auxin signaling [36]. Our degradome analysis also showed that these two peach tasiARFs targeted two ARF transcription factors (ppa001557m and ppa001392m) (Figure 4c), indicating functional conservation in peach.

A peach *TAS4* ortholog, defined as *PpTAS4*, was also identified. Its transcript bore a miR828 signature binding site at the 5' end with a similar siRNA biogenesis pattern (Figure 4b) and *PpTAS4* siRNA was preferentially produced in the leaf and flower (Figure 4b). One of *TAS4*-derived siRNAs, *TAS4*-siRNA(−81), shared extensive sequence identity with its *Arabidopsis* counterpart which has been shown to target at least three *MYB*s that up-regulate anthocyanin production [37]. Our degradome analysis did not identify any target for the peach *TAS4*-siRNA(−81), but *in silico* analysis predicted at least two *MYB*s (ppa024617m and ppa022808m) as targets for peach *TAS4*-siRNA(−81). These predicted *MYB* targets are closely related to *AtMYB113* which is targeted by *Arabidopsis TAS4*-siRNA(−81) [38].

### Multiple miRNA targeting of MYB transcription factors with diverse functions in peach

**Figure 5:**
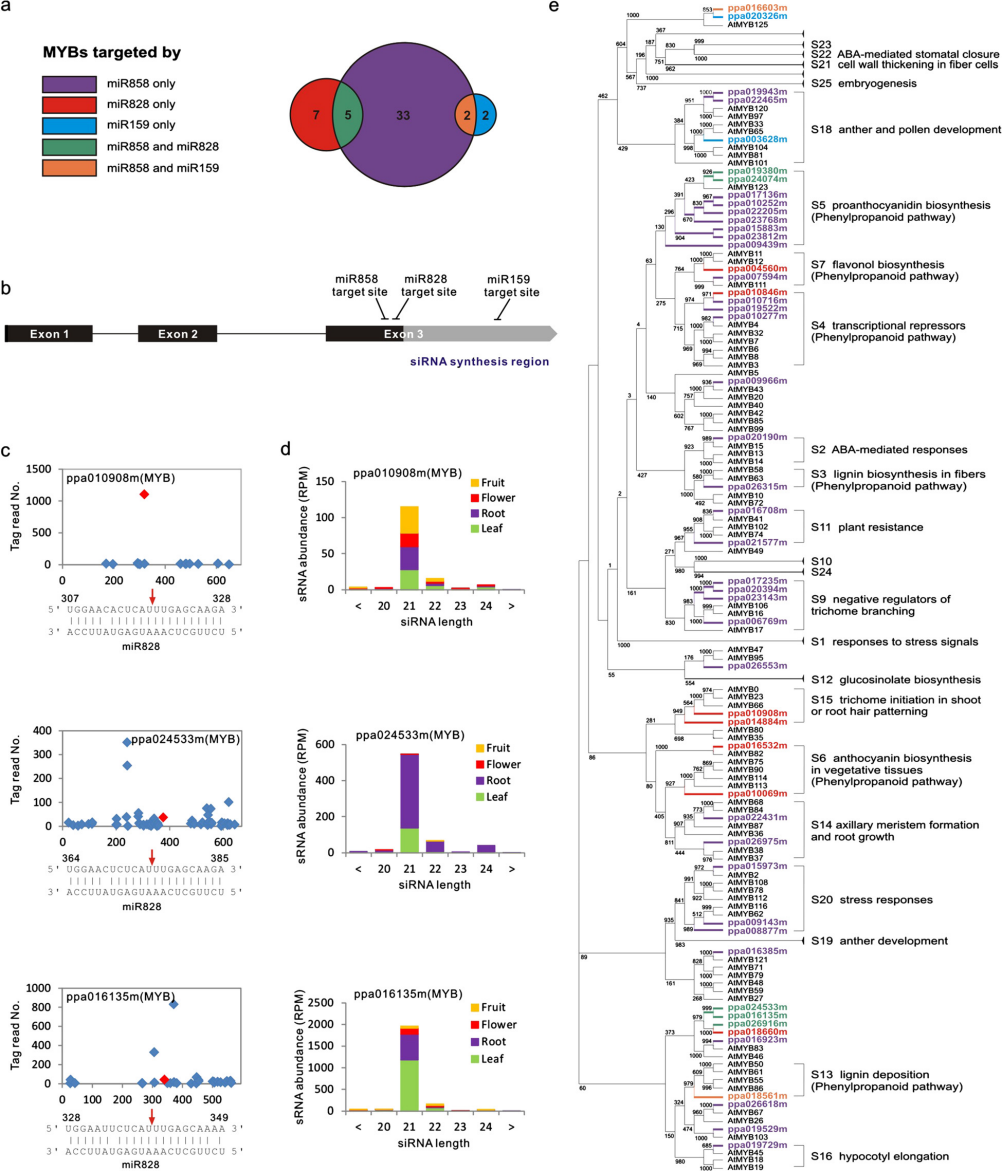
**Three MiRNAs target 49 peach MYBs.** (**a**) It was found that 49 *MYB*s were targeted by peach miR159, miR828 and miR858, some of which were targeted by more than one of the miRNAs. MiR858 targeted the majority of these *MYB*s. (**b**) Genomic organization of R2R3 *MYB* genes, location of target sites of miR159, miR828 and miR858, as well as a potential siRNA generation region. The highly conserved sequences are denoted by black area while the diverged sequence by gray box along the *MYB* coding regions. (**c**) Degradome confirmation of miR828 cleavage in three *MYB* transcripts. The red diamond and arrow indicate the cleavage site. (**d**) The tissue-specific accumulation of the phased siRNAs produced from the miR828-cleaved *MYB* transcripts is shown, as illustrated in Figure 4. (**e**) Phylogenetic analysis of functional relationship between miRNA-targeted peach R2R3 *MYB*s and the characterized *Arabidopsis* R2R3 *MYB*s according to previously work [39,40]. *MYB* genes targeted by specific miRNA are differentiated by the same colors, as illustrated in (a).

In *Arabidopsis*, miR159, miR828 and miR858 target at least 13 *MYB* genes [39]. Our degradome data found that these three miRNAs collectively targeted 19 *MYB*s in peach (Table 2). However, considering that the miRNA target number was underestimated, due to no or low expression of target genes in the specific tissues, it is likely that more *MYB* gene targets exist. Therefore, we performed *in silico* target prediction and identified an additional three, nine and 24 *MYB* genes for miR159, miR828 and miR858, respectively, with an align score of less than 5. Thus, a total of 49 *MYB* target genes were found, four for miR159, 12 for miR828 and 40 for miR858. MiR858 shared five targeted *MYB*s with miR828 and two with miR159 (Figure 5a). Most *MYB* genes that we confirmed or predicted as miRNA targets belonged to the R2R3-MYB class, sharing a similar genomic organization with a conserved 5' region and a divergent region at the 3' end (Figure 5b). Further analysis revealed that miR828 and miR858 target sites were separated by 12 nucleotides and co-located in the conserved region of the third exon while the miR159 target site was located in the divergent region of the co-targeted *MYB*s (Figure 5b). Furthermore, we found that the miR828-cleaved transcripts of three *MYB*s underwent phased 21-nt siRNA biogenesis production (Figure 5c). These three *MYB* transcripts shared similar miR828 cleavage positions, tasiRNA generation regions and patterns, intron-exon structure and sequence conservation (Figure 5b). The produced tasiRNAs displayed quite different tissue specificity; the majority of ppa024533m-derived tasiRNAs were found in root, the majority of ppa016135m-derived tasiRNAs were primarily in leaf and ppa010908m-derived tasiRNAs were distributed mostly in fruit (Figure 5d).

A large number of R2R3 *MYB* genes comprising 25 subgroups have been functionally characterized in *Arabidopsis*[39,40]. Accordingly, we did a phylogenetic analysis for all the miRNA-targeted peach *MYB* genes and found that of the four miR159-targeted *MYB*s, one was in *MYB* subgroup 18 - anther and pollen development; another co-targeted by miR858 in subgroup 13 - lignin deposition, mucilage production and stomatal aperture [39], and the remaining two were ungrouped. Twelve miR828-targeted *MYB*s grouped into five subgroups, i.e. S4, S5, S6, S7 - anthocyanin biosynthesis and S15 - trichome initiation (Figure 5e). The 40 *MYBs* targeted by miR858 fell into 11 subgroups, which were shown to regulate diverse biological processes, including organ morphogenesis, lignification, anthocyanin biosynthesis and plant response to stresses in *Arabidopsis* (Figure 5e). Therefore, miR858 could play a more fundamental and diverse role in peach, compared to miR159 or miR828. Interestingly, of the 49 *MYB*s, 19 were related to phenylpropanoid metabolism, which is a key pathway associated with stone hardening and fruit color development, suggesting an important role of these miRNAs in regulation of peach fruit development and ripening.

## Discussion

### Peach miRNAs and their targets with diverse biological significance

In plants, miRNAs are both highly conserved and rapidly evolving, and these features have been characterized in a variety of lineages [25]. While extensive research has been carried out on model plants, less is known about the characterization and functional analysis of miRNAs from plant species with agricultural and horticultural significance such as peach and apple [31,41,42]. An earlier study reported computational and experimental identification of eight miRNAs from seven conserved miRNA families in peach [31], not extensive compared to those identified in other plant species [18-21]. Here we provided a comprehensive analysis of peach miRNAs produced in different tissues and characterized their expression patterns by both sRNA-seq and RNA blot analyses. Most of the identified peach miRNAs were found to be tissue-specific, as previously observed for other plant species [9,19-21]. Many known miRNAs targets, mostly transcription factors, control diverse physiological processes and genetic programs associated with leaf polarity, lateral root formation, flowering, metabolism and stress responses [36,39,43,44]. In this study, a majority of the targets for peach miRNAs have counterparts previously identified in other species [18-23]. For example, two gene targets identified for miR160 in peach are homologus to those in *Arabidopsis* encoding two auxin response factors (ARFs) that act as repressors in auxin signaling, while a target for miR167 in peach is homologous to *AtARF6* which is an activator in the same pathway [36]. Peach tasiARFs target genes that encode auxin signal repressors as their *Arabidopsis* counterpart does. Further, miR393 that targets TIR1/AFB2 Auxin Receptor (TAAR) Clade, another class of key players for auxin signaling [45] was also found to target three TAAR homologs in peach. Evidently, miRNA- and tasiARF-mediated regulation of the auxin signaling pathway previously characterized in *Arabidopsis* is also conserved in peach. However, some of the identified known miRNAs were found to have additional or novel gene targets in peach (Table 2). For example, miR396 that is known to target a conserved family of growth-regulating factor genes also targeted a rho guanyl-nucleotide exchange factor that is involved with phytochrome signaling. Likewise, miR408, besides targeting the conserved copper ion binding protein, could also target two genes encoding a selenium-binding protein and a D-type cyclin, which regulate plant defense and growth, respectively [46,47]. These results suggest that some known miRNAs have either acquired new targets or expanded their regulatory functions in peach.

Our work found 47 peach-specific miRNAs, which were differentially regulated in various tissues and/or developmental stages (Figure 2c) and targeted a variety of genes with a wide range of biological functions. For example, miRC6a/b targeted two genes encoding pentatricopeptide (PPR) repeat-containing proteins involved in RNA editing, splicing and translation within mitochondria and chloroplasts [48,49]. MiRC3 and miRC5 both targeted the same NBS-LRR disease resistance protein [50]. Other peach-specific miRNAs were found to target genes associated with transcription/translation initiation, protein kinases, and esterase/lipase/thioesterase family proteins. MiRC45 was found to target a transcript encoding *VIP* (*VERNALIZATION INDEPENDENCE*), a gene that modulates vernalization in *Arabidopsis*[51,52]. We identified targets for only 12 of the 47 peach-specific miRNAs, which raises a question of whether these remaining miRNAs are non-functional, or alternatively regulate gene expression by translational repression, not direct cleavage of target mRNAs [53,54]. Conceivably, further improvement of degradome analysis sensitivity and detailed examination of possible translational repression functions of these miRNAs in peach or transgenic plants would provide information of whether or not these miRNAs are functional in peach.

### Differential processing of miRNA precursors in peach

In animals, many miRNAs with distinct spatio-temporal expression patterns are modulated by transcriptional and/or post-transcription regulation [55]. In plants though, miRNA transcripts are thought to be immediately processed into mature miRNAs through the processing machinery (DCL1, HYL1, or SE) inside the nucleus, hence the transcript precursor processing is coupled with the transcription [6]. However, a recent study in maize showed that transcription was not closely associated with transcript precursor processing for two miRNAs in specific tissues. The accumulation of abundant miR166a transcript precursors but not mature miR166a was detected in the tip of the maize shoot apical meristem [56]. Transcript precursors were observed for miR390 by RT-PCR in the L1 but not the L2 layer of the shoot apical meristem while similar levels of mature miR390 was revealed in the same L1 and L2 layers by *in situ* hybridization [56]. These results suggest that both miR166a and miR390 precursors could be differentially processed in various cell types or tissues, although other possibilities, including miRNA stability and mobility, could not be ruled out [56,57]. In this study, we detected differential accumulation of mature miRNAs and their transcript precursors for nine peach miRNAs (Additional file 7: Figure S2). By comparing the miRNA precursor processing efficiency among tissues for two chosen miRNAs (miRC1 and miRC26), we found that the processing efficiency of their transcript precursors varied in root, bark, leaf, flower, as well as during fruit development (Figure 3), demonstrating that differential processing modulates miRC1 and miRC26 expression. We also found that there was no correlation between miRNA transcription and precursor processing activities thereby demonstrating the two processes can likely be uncoupled. Our detection of these larger transcripts provided substantial evidence for differential processing, though still not conclusive, suggesting this might be part of the basis for the miRNA expression in specific tissues or developmental stages in peach. Similar observations were found during apple miRNA identification indicating such a regulatory mechanism may be common in fruit crops.

Although the mechanism underlying differential processing of miRNA precursors in plants remains unknown, it has been characterized in animal cells where miRNA biogenesis is initiated by processing the pri-miRNA transcript into miRNA precursors (pre-miRNA) by the microprocessor complex containing the dsRNA-binding protein DGCR8 and the RNase III enzyme Drosha as well as multiple accessory proteins [55,58]. The processed pre-miRNAs are exported to the cytoplasm and cleaved into mature 22-nt miRNAs by Dicer proteins. Two groups of protein factors that utilize distinct strategies to modulate differential processing of miRNA precursors have been identified and characterized. Group one factors (SMADs and p53) interact with the microprocessor complex and accessory factors (EWSR1, p68 and p72) to activate or repress the processing of pri-miRNAs [59-62]. Group two factors (Lin28, hnRNPs, KSRP and TRBP) recognize and bind to the terminal loop of specific miRNA precursors to facilitate the processing or degradation of the bound miRNA precursors in specific tissues or cell types [63-65]. Conceivably, various factors expressed in specific tissues or developmental stages could directly or indirectly interact in the same manner to modulate the processing of specific miRNA precursors in peach. Our findings here implied a prevailing differential stabilization of miRNA precursors, most likely to be dependent on the tissue-specific action of DCLs, HYLs, HENs and other RNA-binding proteins.

### The conserved *trans*-acting siRNA pathway in peach

To date, four *TAS* gene families have been characterized in *Arabidopsis*, of which the miR390-*TAS3* and miR828-*TAS4* pathways are conserved in plants [3,4]. Here we identified both *TAS3* and *TAS4* peach orthologs, together with their corresponding trigger miRNAs (Figure 4a,b). We also found similar siRNA biogenesis patterns in the cleaved *TAS3* and *TAS4* transcripts. The tasiRNA species are conserved as evidenced by the identification of homologous tasiARFs and *TAS4*-siRNA(−81) and their targets either confirmed by degradome analysis (Figure 4c) and/or *in silico* prediction. Together, these data indicate that both miR390-*TAS3* and miR828-*TAS4* biogenesis pathways and functions are at least partially conserved in peach. Since auxin signaling and modulation is essential for diverse biological processes in peach, especially for fruit development and ripening [66,67], miR390-*TAS3* biogenesis-derived tasiARFs in specific tissues could orchestrate auxin signaling that could be directly relevant to fruit growth and development. In *ArabidopsisTAS4*-siRNA(−81) has been shown to target genes (*AtMYB75AtMYB90* and *AtMYB113*) that positively regulate anthocyanin production [9,38]. The induction of *AtMYB75* along with anthocyanin accumulation activates miR828, *TAS4* and *TAS4*-siRNA(−81) [37]. This feedback regulatory loop is proposed to maintain proper anthocyanin levels in plant tissues under nutrient stress condition [38]. This role of *TAS4*-siRNA(−81) could also be important in peach, where anthocyanin production is directly related to fruit color, which can be highly variable and is considered as an important fruit quality trait [29,68]. While direct gene targets for *TAS4*-siRNA(−81) have not been confirmed by the degradome analysis, *in silico* prediction showed that *TAS4*-siRNA(−81) can target at least two peach *MYB*s highly homologous to *AtMYB113*. Thus, miR828, *TAS4TAS4*-siRNA(−81) and the targeted *MYB*s could form a similar feedback regulatory circle that control anthocyanin accumulation and possibly fruit coloration during peach fruit ripening, which is further supported by the observation of detectable miR828 expression in the mature but not the young fruit (Figure 2b).

### MiRNA-mediated MYB regulatory networks in peach and their biological relevance

While *MYB*s are known to be targeted by miRNAs in other species [9,21,38], the target number is limited [39]. In peach, at least 49 *MYB*s can be potentially targeted by miR159, miR828 and miR858 (Figure 5a). This expansion may have to do with the specialized developmental programs that potentially are regulated through specific *MYB*s, including but not limited to the lignin synthesis and deposition that orchestrates the stone hardening process and the flavonoid synthesis that is important to fruit color, nutritive properties and disease resistance [29,69-71]. The lignin and flavonoid biosynthesis pathways are biochemically competitive, drawing from the same phenylpropanoid precursors. Thus, they need to be tightly coordinated during peach fruit development to enable efficient phenylpropanoid metabolism in a tissue specific fashion [29]. While we were only able to detect miR828 expression during fruit development (Figure 2b), we cannot rule out the potential roles of miR858 and miR159 since they could be highly cell- or tissue-, or stage-specific during fruit development, and their expression window period might be missed in this study. Still, the potential regulatory roles of miR858, miR159 and miR828 in lignin, cell wall and flavonoid metabolism and synthesis pathways provides evidence for a significant role of sRNA in coordinating fruit development.

The finding that miR858 shares five *MYB* targets with miR828 and two with miR159, respectively, and three miR828-targeted *MYB*s undergo siRNA biogenesis supports the notion of the evolution of a miRNA- and siRNA-mediated silencing reinforcement regulatory mechanism in peach. The co-targeting of the same *MYB*s by two miRNAs is expected to strengthen their silencing function while miRNA cleavage followed by siRNA biogenesis reinforces the same silencing effect. Although these are two unrelated biological events, they achieve the same goal, executing a strong responsive regulatory function; hence the miRNA-mediated co-targeting could also enable the targeted *MYB*s to be under refined spatio-temporal regulation. Thus the observed distinct expression patterns of miR828 and miR858 among various tissues and fruit developmental stages would modulate the co-targeted *MYB* expression in an exquisite spatio-temporal manner, to precisely regulate the co-ordination of lignification in stone and mesocarp- and ectocarp-specific fruit coloring during peach fruit development and ripening.

## Conclusions

We characterized miRNAs and their potential targets in peach to provide a comprehensive list of peach miRNAs and reveal the potential scope of their regulatory functions. We show that peach has both conserved and species-specific miRNAs with distinct expression patterns, and that these miRNAs potentially target dozens of genes with a wide range of biological functions. Quite a few of the peach-specific miRNA precursors are subject to differential processing in various tissues and during fruit development, indicating possible mechanisms that define the extent of miRNA accumulation in a spatio-temporal manner. Further, both miR390-*TAS3* and miR828-*TAS4* siRNA biogenesis pathways and their functions appear to be conserved in peach; miR828 cleavage is capable of activating siRNA biogenesis in *PpTAS4* and three MYB protein-coding transcripts, indicating a silencing reinforcement in peach. In addition, we found that miR159, miR828 and miR858 collectively target 49 *MYB*s, 19 of which are known to regulate phenylpropanoid metabolism, a key pathway involved in stone hardening and fruit color development. In summary, we extensively characterize the unique expression pattern and processing regulation of peach miRNAs, and demonstrate the presence of a complex miRNA regulatory network capable of targeting a wide variety of biological functions in peach. Our results provide new and valuable information for deciphering the intricate roles of peach miRNAs and tasiRNAs in gene regulation, which will be useful for the further investigation of miRNA and tasiRNA functions in other crop species.

## Methods

### Plant materials

Root, bark, leaf, flower and fruits of different developmental stages (19, 40 and 82 days after bloom) were collected from *Prunus persica* cv. Lovell peach trees, planted at Appalachian Fruit Research Station, Kearneysville, WV. All the samples were immediately frozen in liquid nitrogen and stored at −80°C until use.

### RNA preparation and deep sequencing

Total RNA was extracted, using Plant RNA Purification Reagent (Invitrogen, CA, USA), from four different peach tissues, i.e. root, leaf, flower and mixed fruits of various developmental stages. The small RNA and degradome library construction were performed by BGI (Beijing Genomics Institute, China). In brief, the small RNAs of 18~30-nt were isolated from total RNA and ligated to a 5' RNA adapter and a 3' RNA adapter, as described previously [72]. A reverse transcription reaction followed by PCR was performed and the amplified library then underwent gel purification prior to sequencing on SOLID system or Illumina Hiseq 2000 platform.

The peach degradome library was constructed as previously described [73], with pooled total RNA from peach root, leaf, flower and mixed fruits. In brief, poly(A) RNA was extracted and ligated to a 5' RNA adapter and the products were digested after RT-PCR and ligated to a 3' dsDNA adapter. The amplified library was then gel-purified for sequencing on Illumina Hiseq 2000 platform.

The small RNA library and degradome library sequencing data are available under NCBI-GEO accession no: GSE38535.

### RNA blot analysis

For RNA blot analysis, 25 μg of total RNA from leaf, flower, fruit at 19 day after bloom (DAB), 40 DAB, 82 DAB, root and bark was separated on a denaturing 15% polyacrylamide gel. The RNA was blotted onto Amersham Hybond^TM^-NX membranes (GE Healthcare, Waukesha, WI, USA) and crosslinked using EDC (Sigma, St. Louis, MO, USA). DNA oligonucleotides probes (Additional file 8: Table S6) that are reverse complementary to miRNAs were labeled with γ^32^P-ATP using T4 polynucleotide kinase (NEB, Beverly, MA, USA). MicroRNA Marker Probe (NEB, Beverly, MA, USA) was used for size determination. MiR172 was selected as a loading control for all the RNA blots because it gave the most consistent signal among different tissues compared to other genes we tested, including U6 (Figure 2a). The membranes were hybridized at 42°C for overnight and washed twice at 55°C with washing buffer containing 2 × SSC and 2% SDS. The membranes were then exposed to the phosphorscreens and scanned with Typhoon TRIO Variable Mode Imager (GE Healthcare, Waukesha, WI, USA). We stripped the membranes with probe stripping solution and then exposed the stripped membranes overnight to the phosphorscreens to ensure that there was no trace of radiation signal detected before re-hybridizing with a new probe. Many of the hybridizations were repeated with a second blot to verify the results.

### Bioinformatics analysis

All the sequencing data was processed by removing the 3’ adaptor using CLC Genomic Workbench 4.9 (CLC Bio., Aarhus, Denmark). Any sequences without adaptor sequence were excluded from analyses. Reads homologous to non-coding RNAs and conserved miRNAs were removed by BLATN alignment against Rfam 10 (http://www.sanger.ac.uk/resources/databases/rfam.html) and mature miRNAs in miRBase (http://www.mirbase.org, release 18), allowing up to two mismatches. The remaining sRNAs were subjected to peach-specific miRNA identification. Read mapping was conducted using Bowtie [74], and Vienna RNA package was used for miRNA secondary structure prediction [75]. Those sRNAs (20~22-nt) with a canonical stem-loop structure (no more than four mismatches, and no more than one central bulge) and a miRNA/miRNA* pair accounting for over 75% of the reads matching to the respective precursor locus were considered as potential peach-specific miRNAs. A detailed screening criterion was applied according to Meyers *et al*. [76]. The total number of the reads perfectly matching the peach genome in a given library was used for the normalization of read abundance, denoted as rpm (reads per million). The degradome analysis and target categorization were performed using CLEAVE-LAND pipeline 2.0 [34,77] and Targetfinder 1.6 (http://carringtonlab.org/resources/targetfinder). The alignment score threshold was set to 4.5 for conserved and less-conserved miRNAs, and to 5 for novel and candidate miRNAs. The peach genome scaffold, CDS sequence and gene annotation information were retrieved from GDR (http://www.rosaceae.org).

### Multiple alignment and phylogenetic analysis

Multiple alignments were conducted using CLUSTAL X2 [78]. All the peach *MYB* targets for miR828, miR858, and miR159 were predicted by Targetfinder 1.6 with an align-score of no more than 5. Amino acid sequences of MYB factors in *Arabidopsis* were retrieved from TAIR (http://www.arabidopsis.org) and a phylogenetic tree was generated using the neighbor-joining method and 1000 bootstraps with putative full-length sequences using CLUSTAL X2 [78]. The subgroup and function annotation were designated according to Dubos *et al*. [39].

## Competing interests

The authors declare that they have no competing interests.

## Authors’ contributions

HZ and ZL initiated the research. HZ, ZL, BZ and YA designed the experiments. RX performed the computational analyses. HZ and ZL carried out the biological experiments, interpreted the results and prepared the manuscript. CDD and AMC provided extensive intellectual suggestion for the manuscript organization and writing. All authors critically read and approved the final version of the manuscript.

## Supplementary Material

Additional file 1 **Table S1.** Statistics of sRNA sequences from peach root, leaf, flower and fruit. Click here for file

Additional file 2 **Table S2.** Read length distribution for each conserved miRNA family recovered from peach sRNA libraries. Click here for file

Additional file 3 **Table S3.** Peach homologs of known miRNAs. Click here for file

Additional file 4 **Table S4.** Known miRNAs with canonical stem-loop structure predicted from peach sRNA libraries. Click here for file

Additional file 5 **Figure S1.** Stem-loop structures for peach miRNAs. Click here for file

Additional file 6 **Table S5.** A detailed list of peach-specific miRNAs. Click here for file

Additional file 7 **Figure S2.** Detection of pre-miRNAs in peach. Click here for file

Additional file 8 **Table S6.** Peach miRNA probes for RNA blot analysis. Click here for file

## References

[B1] Chen XM Small RNAs and Their Roles in Plant Development Annu Rev Cell Dev Biol 2009 25 21 44 10.1146/annurev.cellbio.042308.113417 19575669 PMC5135726

[B2] Jinek M Doudna JA A three-dimensional view of the molecular machinery of RNA interference Nature 2009 457 7228 405 412 10.1038/nature07755 19158786

[B3] Allen E Howell MD miRNAs in the biogenesis of trans-acting siRNAs in higher plants Semin Cell Dev Biol 2010 21 8 798 804 10.1016/j.semcdb.2010.03.008 20359543

[B4] Axtell MJ Jan C Rajagopalan R Bartel DP A two-hit trigger for siRNA biogenesis in plants Cell 2006 127 3 565 577 10.1016/j.cell.2006.09.032 17081978

[B5] Lu C Jeong DH Kulkarni K Pillay M Nobuta K German R Thatcher SR Maher C Zhang L Ware D Genome-wide analysis for discovery of rice microRNAs reveals natural antisense microRNAs (nat-miRNAs) Proc Natl Acad Sci USA 2008 105 12 4951 4956 10.1073/pnas.0708743105 18353984 PMC2290808

[B6] Voinnet O Origin, Biogenesis, and Activity of Plant MicroRNAs Cell 2009 136 4 669 687 10.1016/j.cell.2009.01.046 19239888

[B7] Allen E Xie ZX Gustafson AM Carrington JC microRNA-directed phasing during trans-acting siRNA biogenesis in plants Cell 2005 121 2 207 221 10.1016/j.cell.2005.04.004 15851028

[B8] Yoshikawa M Peragine A Park MY Poethig RS A pathway for the biogenesis of trans-acting siRNAs in Arabidopsis Gene Dev 2005 19 18 2164 2175 10.1101/gad.1352605 16131612 PMC1221887

[B9] Rajagopalan R Vaucheret H Trejo J Bartel DP A diverse and evolutionarily fluid set of microRNAs in Arabidopsis thaliana Gene Dev 2006 20 24 3407 3425 10.1101/gad.1476406 17182867 PMC1698448

[B10] Jones-Rhoades MW Bartel DP Bartel B MicroRNAs and their regulatory roles in plants Annu Rev Plant Biol 2006 57 19 53 10.1146/annurev.arplant.57.032905.105218 16669754

[B11] Lu C Tej SS Luo SJ Haudenschild CD Meyers BC Green PJ Elucidation of the small RNA component of the transcriptome Science 2005 309 5740 1567 1569 10.1126/science.1114112 16141074

[B12] Lewis BP Burge CB Bartel DP Conserved seed pairing, often flanked by adenosines, indicates that thousands of human genes are microRNA targets Cell 2005 120 1 15 20 10.1016/j.cell.2004.12.035 15652477

[B13] Bowman JL Axtell MJ Evolution of plant microRNAs and their targets Trends Plant Sci 2008 13 7 343 349 10.1016/j.tplants.2008.03.009 18502167

[B14] Sunkar R Chinnusamy V Zhu JH Zhu JK Small RNAs as big players in plant abiotic stress responses and nutrient deprivation Trends Plant Sci 2007 12 7 301 309 10.1016/j.tplants.2007.05.001 17573231

[B15] Llave C Kasschau KD Rector MA Carrington JC Endogenous and silencing-associated small RNAs in plants Plant Cell 2002 14 7 1605 1619 10.1105/tpc.003210 12119378 PMC150710

[B16] Sunkar R Zhu JK Novel and stress-regulated microRNAs and other small RNAs from Arabidopsis Plant Cell 2004 16 8 2001 2019 10.1105/tpc.104.022830 15258262 PMC519194

[B17] Zhu QH Spriggs A Matthew L Fan LJ Kennedy G Gubler F Helliwell C A diverse set of microRNAs and microRNA-like small RNAs in developing rice grains Genome Res 2008 18 9 1456 1465 10.1101/gr.075572.107 18687877 PMC2527712

[B18] Barakat A Wall PK Diloreto S Depamphilis CW Carlson JE Conservation and divergence of microRNAs in Populus BMC Genomics 2007 8 481 10.1186/1471-2164-8-481 18166134 PMC2270843

[B19] Moxon S Jing RC Szittya G Schwach F Pilcher RLR Moulton V Dalmay T Deep sequencing of tomato short RNAs identifies microRNAs targeting genes involved in fruit ripening Genome Res 2008 18 10 1602 1609 10.1101/gr.080127.108 18653800 PMC2556272

[B20] Zhang LF Chia JM Kumari S Stein JC Liu ZJ Narechania A Maher CA Guill K McMullen MD Ware D A Genome-Wide Characterization of MicroRNA Genes in Maize PLoS Genet 2009 5 11 e1000716 10.1371/journal.pgen.1000716 19936050 PMC2773440

[B21] Pantaleo V Szittya G Moxon S Miozzi L Moulton V Dalmay T Burgyan J Identification of grapevine microRNAs and their targets using high-throughput sequencing and degradome analysis Plant J 2010 62 6 960 976 20230504 10.1111/j.0960-7412.2010.04208.x

[B22] Zhao CZ Xia H Frazier TP Yao YY Bi YP Li AQ Li MJ Li CS Zhang BH Wang XJ Deep sequencing identifies novel and conserved microRNAs in peanuts (Arachis hypogaea L.) BMC Plant Biol 2010 10 3 10.1186/1471-2229-10-3 20047695 PMC2826338

[B23] Song QX Liu YF Hu XY Zhang WK Ma BA Chen SY Zhang JS Identification of miRNAs and their target genes in developing soybean seeds by deep sequencing BMC Plant Biol 2011 11 5 10.1186/1471-2229-11-5 21219599 PMC3023735

[B24] Axtell MJ Bartel DP Antiquity of microRNAs and their targets in land plants Plant Cell 2005 17 6 1658 1673 10.1105/tpc.105.032185 15849273 PMC1143068

[B25] Cuperus JT Fahlgren N Carrington JC Evolution and functional diversification of MIRNA genes Plant Cell 2011 23 2 431 442 10.1105/tpc.110.082784 21317375 PMC3077775

[B26] Layne DR Bassi D The peach: Botany, production, and uses HortScience 2008 43 4 1190 1191

[B27] Lang GA Dormancy - a New Universal Terminology Hortscience 1987 22 5 817 820

[B28] Horvath DP Anderson JV Chao WS Foley ME Knowing when to grow: signals regulating bud dormancy Trends Plant Sci 2003 8 11 534 540 10.1016/j.tplants.2003.09.013 14607098

[B29] Dardick CD Callahan AM Chiozzotto R Schaffer RJ Piagnani MC Scorza R Stone formation in peach fruit exhibits spatial coordination of the lignin and flavonoid pathways and similarity to Arabidopsis dehiscence BMC Biol 2010 8 13 10.1186/1741-7007-8-13 20144217 PMC2830173

[B30] Ryugo K The rate of dry weight accumulation by the peach pit during the hardening process Amer Soc Hort Sci 1961 78 132 137

[B31] Zhang Y Yu M Yu H Han J Song C Ma R Fang J Computational identification of microRNAs in peach expressed sequence tags and validation of their precise sequences by miR-RACE Mol Biol Rep 2012 39 2 1975 1987 10.1007/s11033-011-0944-6 21667243

[B32] Kurihara Y Watanabe Y Arabidopsis micro-RNA biogenesis through Dicer-like 1 protein functions Proc Natl Acad Sci USA 2004 101 34 12753 12758 10.1073/pnas.0403115101 15314213 PMC515125

[B33] Xie ZX Kasschau KD Carrington JC Negative feedback regulation of Dicer-Like1 in Arabidopsis by microRNA-guided mRNA degradation Curr Biol 2003 13 9 784 789 10.1016/S0960-9822(03)00281-1 12725739

[B34] Addo-Quaye C Miller W Axtell MJ CleaveLand: a pipeline for using degradome data to find cleaved small RNA targets Bioinformatics 2009 25 1 130 131 10.1093/bioinformatics/btn604 19017659 PMC3202307

[B35] Pye VE Christensen CE Dyer JH Arent S Henriksen A Peroxisomal Plant 3-Ketoacyl-CoA Thiolase Structure and Activity Are Regulated by a Sensitive Redox Switch J Biol Chem 2010 285 31 24078 24088 10.1074/jbc.M110.106013 20463027 PMC2911321

[B36] Guilfoyle TJ Hagen G Auxin response factors Curr Opin Plant Biol 2007 10 5 453 460 10.1016/j.pbi.2007.08.014 17900969

[B37] Hsieh LC Lin SI Shih ACC Chen JW Lin WY Tseng CY Li WH Chiou TJ Uncovering Small RNA-Mediated Responses to Phosphate Deficiency in Arabidopsis by Deep Sequencing Plant Physiol 2009 151 4 2120 2132 10.1104/pp.109.147280 19854858 PMC2785986

[B38] Luo QJ Mittal A Jia F Rock CD An autoregulatory feedback loop involving PAP1 and TAS4 in response to sugars in Arabidopsis Plant Mol Biol 2011 80 1 117 129 21533841 10.1007/s11103-011-9778-9 PMC3272322

[B39] Dubos C Stracke R Grotewold E Weisshaar B Martin C Lepiniec L MYB transcription factors in Arabidopsis Trends Plant Sci 2010 15 10 573 581 10.1016/j.tplants.2010.06.005 20674465

[B40] Stracke R Werber M Weisshaar B The R2R3-MYB gene family in Arabidopsis thaliana Curr Opin Plant Biol 2001 4 5 447 456 10.1016/S1369-5266(00)00199-0 11597504

[B41] Varkonyi-Gasic E Gould N Sandanayaka M Sutherland P MacDiarmid RM Characterisation of microRNAs from apple (Malus domestica 'Royal Gala') vascular tissue and phloem sap BMC Plant Biol 2010 10 159 10.1186/1471-2229-10-159 20682080 PMC3095296

[B42] Yu HP Song CN Jia QD Wang C Li F Nicholas KK Zhang XY Fang JG Computational identification of microRNAs in apple expressed sequence tags and validation of their precise sequences by miR-RACE Physiol Plant 2011 141 1 56 70 10.1111/j.1399-3054.2010.01411.x 20875055

[B43] Olsen AN Ernst HA Lo Leggio L Skriver K DNA-binding specificity and molecular functions of NAC transcription factors Plant Sci 2005 169 4 785 797 10.1016/j.plantsci.2005.05.035

[B44] Rodriguez RE Mecchia MA Debernardi JM Schommer C Weigel D Palatnik JF Control of cell proliferation in Arabidopsis thaliana by microRNA miR396 Development 2010 137 1 103 112 10.1242/dev.043067 20023165 PMC2796936

[B45] Si-Ammour A Windels D Arn-Bouldoires E Kutter C Ailhas J Meins F Vazquez F miR393 and Secondary siRNAs Regulate Expression of the TIR1/AFB2 Auxin Receptor Clade and Auxin-Related Development of Arabidopsis Leaves Plant Physiol 2011 157 2 683 691 10.1104/pp.111.180083 21828251 PMC3192580

[B46] Hugouvieux V Dutilleul C Jourdain A Reynaud F Lopez V Bourguignon J Arabidopsis Putative Selenium-Binding Protein1 Expression Is Tightly Linked to Cellular Sulfur Demand and Can Reduce Sensitivity to Stresses Requiring Glutathione for Tolerance Plant Physiol 2009 151 2 768 781 10.1104/pp.109.144808 19710230 PMC2754620

[B47] Murray J D-type cyclins and control of the cell cycle and differentiation in Arabidopsis Comparative Biochem Physiol Part A 2005 141 3 S322

[B48] Saha D Prasad AM Srinivasan R Pentatricopeptide repeat proteins and their emerging roles in plants Plant Physiol Biochem 2007 45 8 521 534 10.1016/j.plaphy.2007.03.026 17560114

[B49] Schmitz-Linneweber C Small I Pentatricopeptide repeat proteins: a socket set for organelle gene expression Trends Plant Sci 2008 13 12 663 670 10.1016/j.tplants.2008.10.001 19004664

[B50] Belkhadir Y Subramaniam R Dangl JL Plant disease resistance protein signaling: NBS-LRR proteins and their partners Curr Opin Plant Biol 2004 7 4 391 399 10.1016/j.pbi.2004.05.009 15231261

[B51] He YH Doyle MR Amasino RM PAF1-complex-mediated histone methylation of FLOWERING LOCUS C chromatin required for the vernalization-responsive, winter-annual habit in Arabidopsis Gene Dev 2004 18 22 2774 2784 10.1101/gad.1244504 15520273 PMC528897

[B52] Liu Y Geyer R van Zanten M Carles A Li Y Horold A van Nocker S Soppe WJ Identification of the Arabidopsis REDUCED DORMANCY 2 gene uncovers a role for the polymerase associated factor 1 complex in seed dormancy PLoS One 2011 6 7 e22241 10.1371/journal.pone.0022241 21799800 PMC3143138

[B53] Brodersen P Sakvarelidze-Achard L Bruun-Rasmussen M Dunoyer P Yamamoto YY Sieburth L Voinnet O Widespread translational inhibition by plant miRNAs and siRNAs Science 2008 320 5880 1185 1190 10.1126/science.1159151 18483398

[B54] Lanet E Delannoy E Sormani R Floris M Brodersen P Crete P Voinnet O Robaglia C Biochemical Evidence for Translational Repression by Arabidopsis MicroRNAs Plant Cell 2009 21 6 1762 1768 10.1105/tpc.108.063412 19531599 PMC2714937

[B55] Newman MA Hammond SM Emerging paradigms of regulated microRNA processing Gene Dev 2010 24 11 1086 1092 10.1101/gad.1919710 20516194 PMC2878647

[B56] Nogueira FT Chitwood DH Madi S Ohtsu K Schnable PS Scanlon MJ Timmermans MC Regulation of small RNA accumulation in the maize shoot apex PLoS Genet 2009 5 1 e1000320 10.1371/journal.pgen.1000320 19119413 PMC2602737

[B57] Bail S Swerdel M Liu H Jiao X Goff LA Hart RP Kiledjian M Differential regulation of microRNA stability RNA 2010 16 5 1032 1039 10.1261/rna.1851510 20348442 PMC2856875

[B58] Carthew RW Sontheimer EJ Origins and Mechanisms of miRNAs and siRNAs Cell 2009 136 4 642 655 10.1016/j.cell.2009.01.035 19239886 PMC2675692

[B59] Davis BN Hilyard AC Lagna G Hata A SMAD proteins control DROSHA-mediated microRNA maturation Nature 2008 454 7200 56 U52 10.1038/nature07086 18548003 PMC2653422

[B60] Fukuda T Yamagata K Fujiyama S Matsumoto T Koshida I Yoshimura K Mihara M Naitou M Endoh H Nakamura T DEAD-box RNA helicase subunits of the Drosha complex are required for processing of rRNA and a subset of microRNAs Nat Cell Biol 2007 9 5 604 U221 10.1038/ncb1577 17435748

[B61] Han JJ Lee Y Yeom KH Kim YK Jin H Kim VN The Drosha-DGCR8 complex in primary microRNA processing Gene Dev 2004 18 24 3016 3027 10.1101/gad.1262504 15574589 PMC535913

[B62] He XY He L Hannon GJ The guardian's little helper: MicroRNAs in the p53 tumor suppressor network Cancer Res 2007 67 23 11099 11101 10.1158/0008-5472.CAN-07-2672 18056431

[B63] He YW Smith R Nuclear functions of heterogeneous nuclear ribonucleoproteins A/B Cell Mol Life Sci 2009 66 7 1239 1256 10.1007/s00018-008-8532-1 19099192 PMC7079774

[B64] Trabucchi M Briata P Garcia-Mayoral M Haase AD Filipowicz W Ramos A Gherzi R Rosenfeld MG The RNA-binding protein KSRP promotes the biogenesis of a subset of microRNAs Nature 2009 459 7249 1010 U1144 10.1038/nature08025 19458619 PMC2768332

[B65] Viswanathan SR Daley GQ Lin28: A MicroRNA Regulator with a Macro Role Cell 2010 140 4 445 449 10.1016/j.cell.2010.02.007 20178735

[B66] Bonghi C Trainotti L Botton A Tadiello A Rasori A Ziliotto F Zaffalon V Casadoro G Ramina A A microarray approach to identify genes involved in seed-pericarp cross-talk and development in peach BMC Plant Biol 2011 11 107 10.1186/1471-2229-11-107 21679395 PMC3141638

[B67] Trainotti L Tadiello A Casadoro G The involvement of auxin in the ripening of climacteric fruits comes of age: the hormone plays a role of its own and has an intense interplay with ethylene in ripening peaches J Exp Bot 2007 58 12 3299 3308 10.1093/jxb/erm178 17925301

[B68] Vizzotto M Cisneros-Zevallos L Byrne DH Okie WR Ramming DW Total Phenolic, Carotenoid, and Anthocyanin Content and Antioxidant Activity of Peach and Plum Genotypes Proc 6th Intl Peach Symposium 2006 713

[B69] Abeles F Biles C Characterization of peroxidases in lignifying peach fruit endocarp Plant Physiol 1991 95 269 273 10.1104/pp.95.1.269 16667963 PMC1077517

[B70] Lombardo VA Osorio S Borsani J Lauxmann MA Bustamante CA Budde CO Andreo CS Lara MV Fernie AR Drincovich MF Metabolic profiling during peach fruit development and ripening reveals the metabolic networks that underpin each developmental stage Plant Physiol 2011 157 4 1696 1710 10.1104/pp.111.186064 22021422 PMC3327199

[B71] Ryugo K Changes in methoxyl content in the peach endocarp and some of its soluble phenolic constituents during lignification Amer Soc Hort Sci 1963 84 110 115

[B72] Lu C Meyers BC Green PJ Construction of small RNA cDNA libraries for deep sequencing Methods 2007 43 2 110 117 10.1016/j.ymeth.2007.05.002 17889797

[B73] Addo-Quaye C Eshoo TW Bartel DP Axtell MJ Endogenous siRNA and miRNA targets identified by sequencing of the Arabidopsis degradome Current Biol 2008 18 10 758 762 10.1016/j.cub.2008.04.042 PMC2583427 18472421

[B74] Langmead B Trapnell C Pop M Salzberg SL Ultrafast and memory-efficient alignment of short DNA sequences to the human genome Genome Biol 2009 10 3 R25 10.1186/gb-2009-10-3-r25 19261174 PMC2690996

[B75] Hofacker IL Vienna RNA secondary structure server Nucleic Acids Res 2003 31 13 3429 3431 10.1093/nar/gkg599 12824340 PMC169005

[B76] Meyers BC Axtell MJ Bartel B Bartel DP Baulcombe D Bowman JL Cao X Carrington JC Chen XM Green PJ Criteria for Annotation of Plant MicroRNAs Plant Cell 2008 20 12 3186 3190 10.1105/tpc.108.064311 19074682 PMC2630443

[B77] Addo-Quaye C Eshoo TW Bartel DP Axtell MJ Endogenous siRNA and miRNA targets identified by sequencing of the Arabidopsis degradome Curr Biol 2008 18 10 758 762 10.1016/j.cub.2008.04.042 18472421 PMC2583427

[B78] Larkin MA Blackshields G Brown NP Chenna R McGettigan PA McWilliam H Valentin F Wallace IM Wilm A Lopez R Clustal W and clustal X version 2.0 Bioinformatics 2007 23 21 2947 2948 10.1093/bioinformatics/btm404 17846036

