# Supplementary material for: Unique expression, processing regulation, and regulatory network of peach (Prunus persica) miRNAs
Source: BMC Plant Biol. 2012 Aug 21;12:149. doi: 10.1186/1471-2229-12-149 (PMC3542160; doi:10.1186/1471-2229-12-149)

Name: ppe-miR156a

Sequence: UGACAGAAGAAAGAGAGCAC

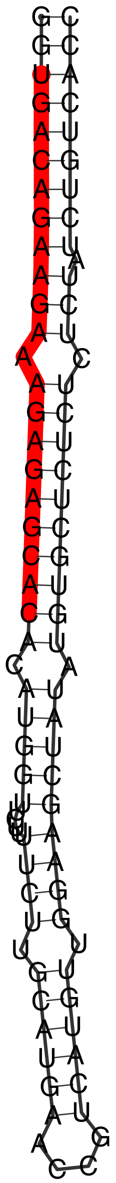

Name: ppe-miR156a

Sequence: UGACAGAAGAAAGAGAGCAC

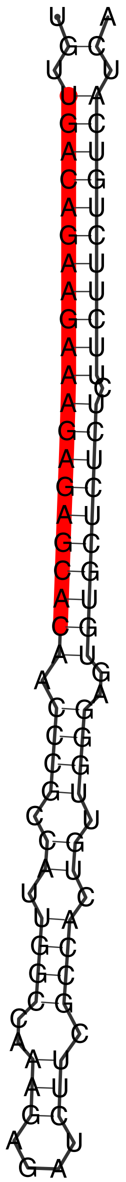

Name: ppe-miR156b

Sequence: UGACAGAAGAGAGUGAGCAC

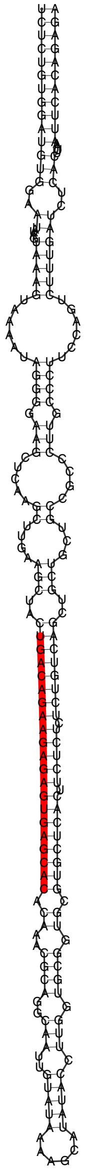

Name: ppe-miR156b

Sequence: UGACAGAAGAGAGUGAGCAC

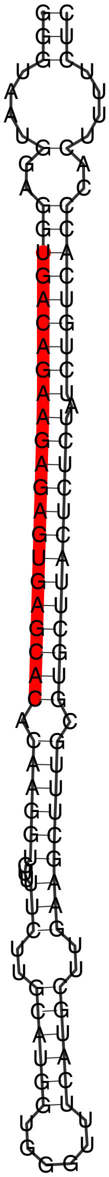

Name: ppe-miR156b

Sequence: UGACAGAAGAGAGUGAGCAC

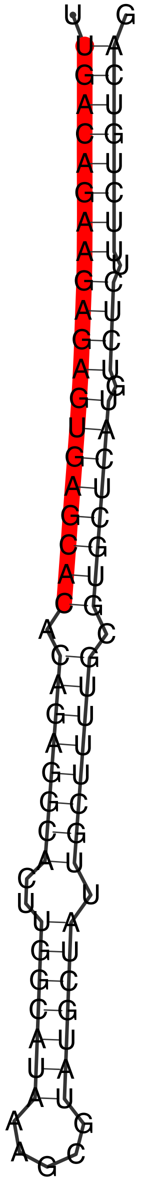

Name: ppe-miR156c

Sequence: UGACAGAAGAUAGAGAGCAC

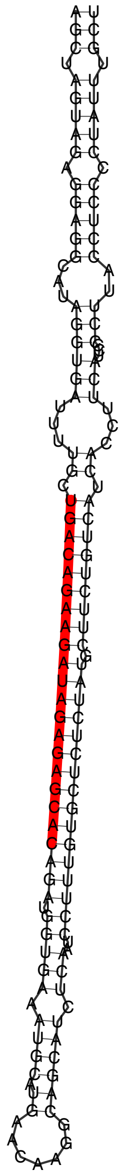

Name: ppe-miR156d

Sequence: UUGACAGAAGAUAGAGAGCAC

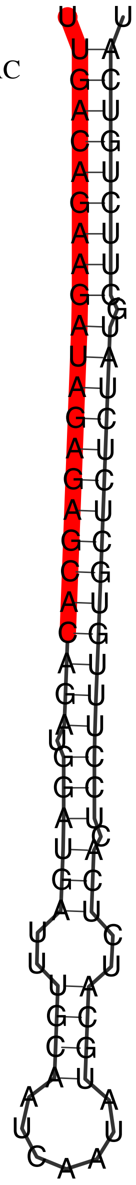

Name: ppe-miR156d

Sequence: UUGACAGAAGAUAGAGAGCAC

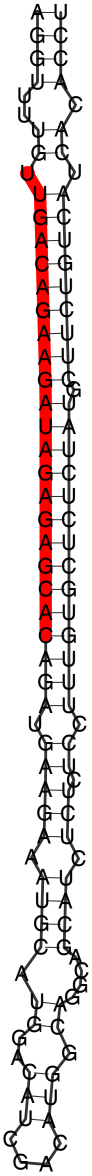

Name: ppe-miR156d

Sequence: UUGACAGAAGAUAGAGAGCAC

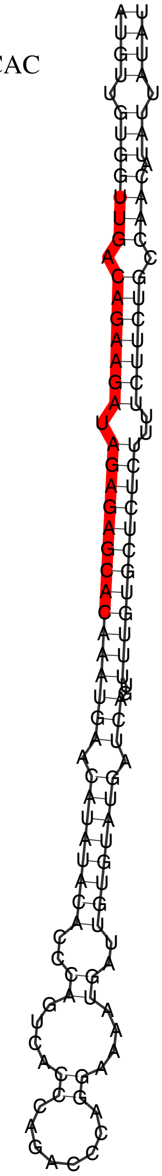

Name: ppe-miR156e

Sequence: UUGACAGAAGAAAGAGAGCAC

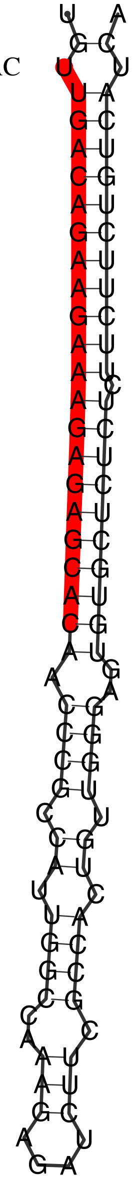

Name: ppe-miR156f

Sequence: CUGACAGAAGAUAGAGAGCAC

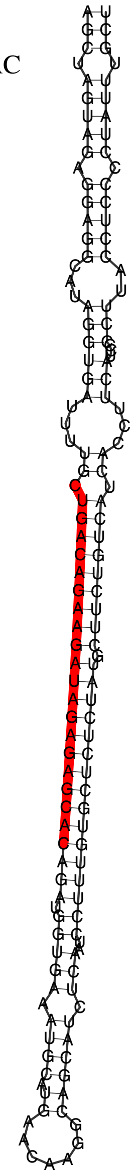

Name: ppe-miR159

Sequence: UUUGGAUUGAAGGGAGCUCUA

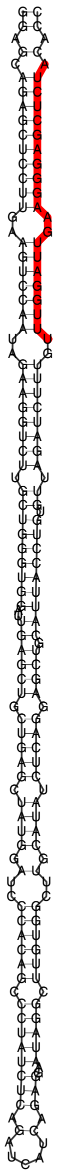

Name: ppe-miR160

Sequence: UGCCUGGCUCCCUGUAUGCCA

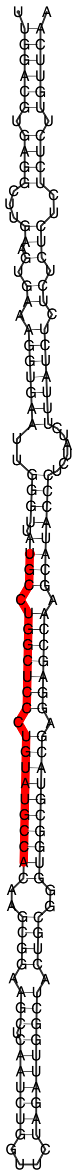

Name: ppe-miR160

Sequence: UGCCUGGCUCCCUGUAUGCCA

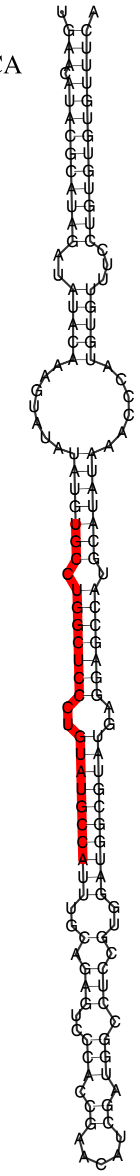

Name: ppe-miR162

Sequence: UCGAUAAACCUCUGCAUCCAG

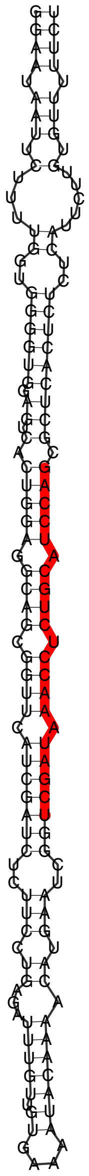

Name: ppe-miR164a

Sequence: UGGAGAAGCAGGGCACGUGCA

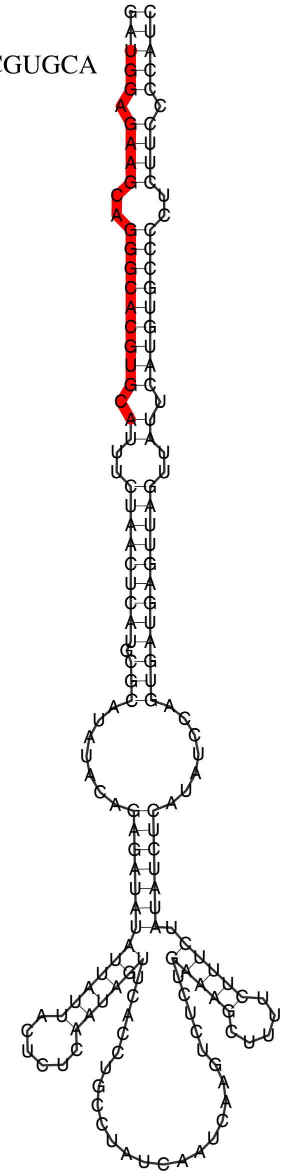

Name: ppe-miR164a

Sequence: UGGAGAAGCAGGGCACGUGCA

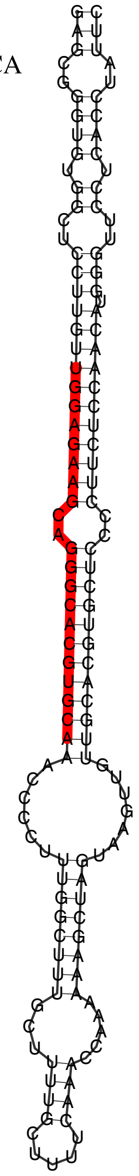

Name: ppe-miR164a

Sequence: UGGAGAAGCAGGGCACGUGCA

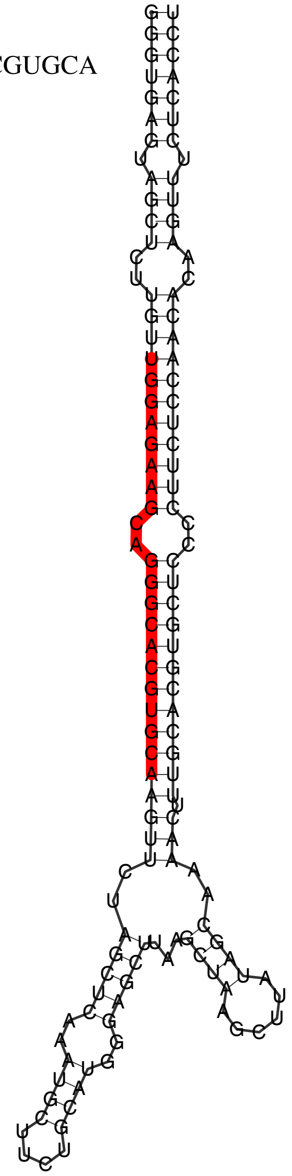

Name: ppe-miR164b

Sequence: UGGAGAAGCAGGGCACAUGCU

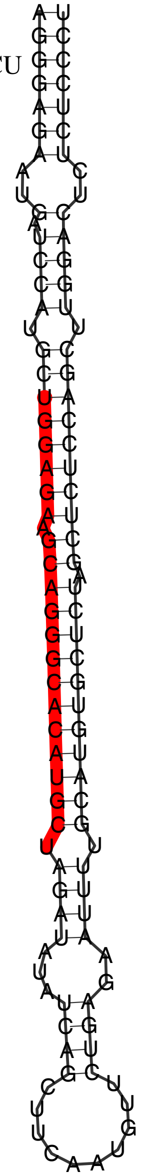

Name: ppe-miR166

Sequence: UCGGACCAGGCUUCAUUCCCC

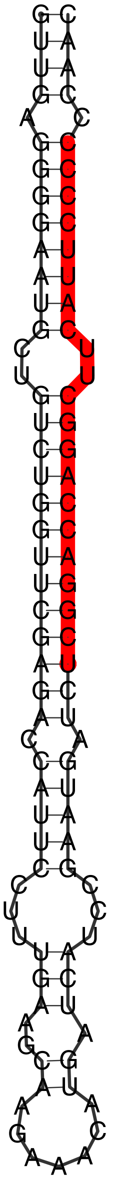

Name: ppe-miR166

Sequence: UCGGACCAGGCUUCAUUCCCC

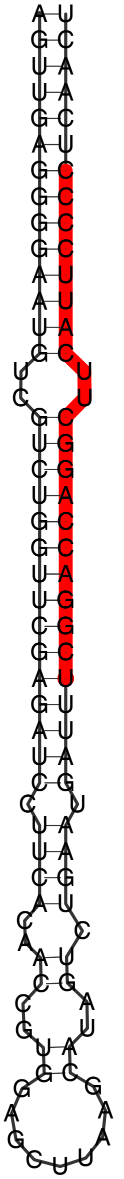

Name: ppe-miR166

Sequence: UCGGACCAGGCUUCAUUCCCC

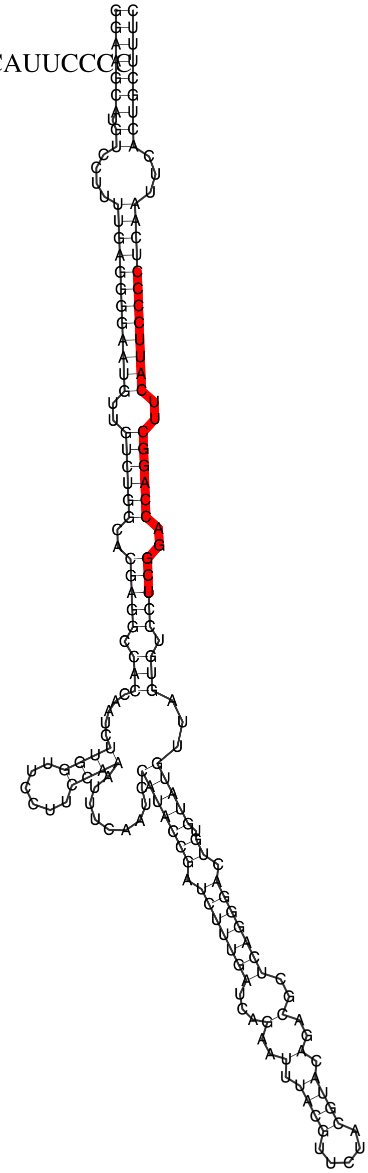

Name: ppe-miR166

Sequence: UCGGACCAGGCUUCAUUCCCC

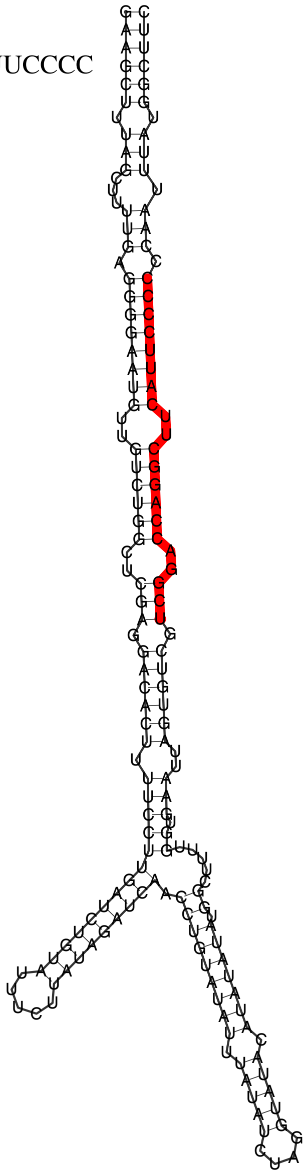

Name: ppe-miR166

Sequence: UCGGACCAGGCUUCAUUCCCC

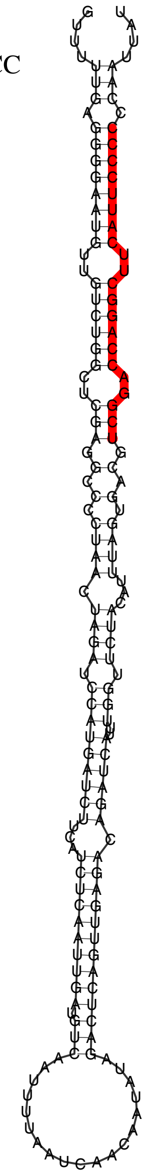

Name: ppe-miR167a

Sequence: UGAAGCUGCCAGCAUGAUCUA

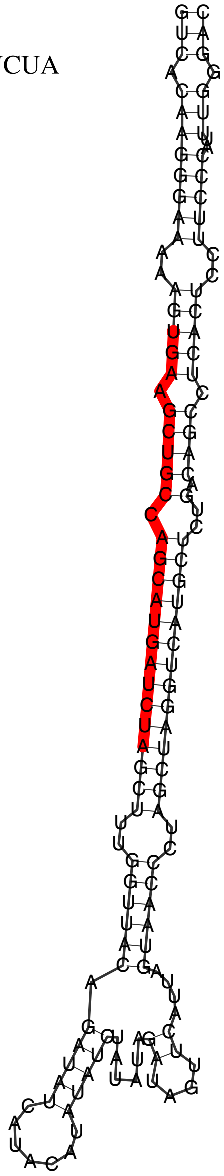

Name: ppe-miR167a

Sequence: UGAAGCUGCCAGCAUGAUCUA

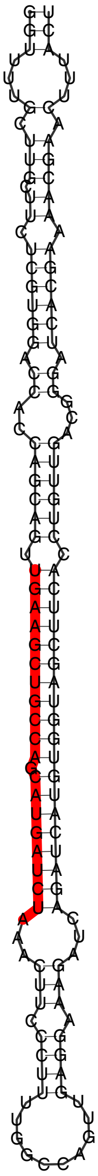

Name: ppe-miR167b

Sequence: UGAAGCUGCCAGCAUGAUCUGA

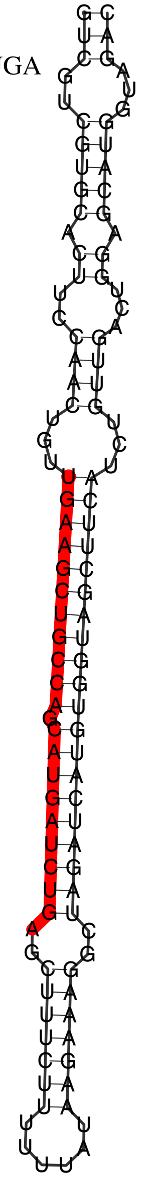

Name: ppe-miR167c

Sequence: UGAAGCUGCCAGCAUGAUCUG

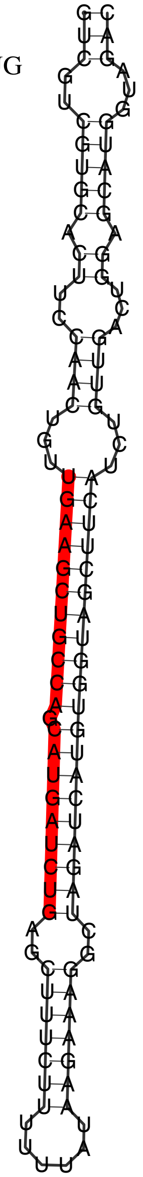

Name: ppe-miR167d

Sequence: UGAAGCUGCCAGCAUGAUCUUA

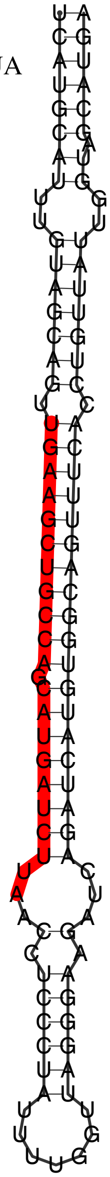

Name: ppe-miR168

Sequence: UCGCUUGGUGCAGGUCGGGAA

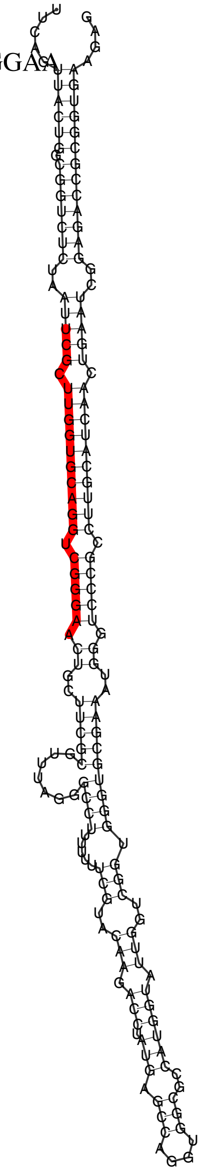

Name: ppe-miR169a

Sequence: CAGCCAAGGAUGACUUGCCGG

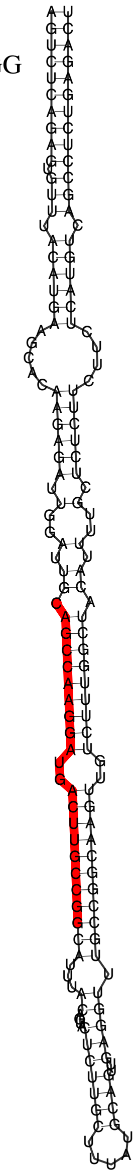

Name: ppe-miR169a

Sequence: CAGCCAAGGAUGACUUGCCGG

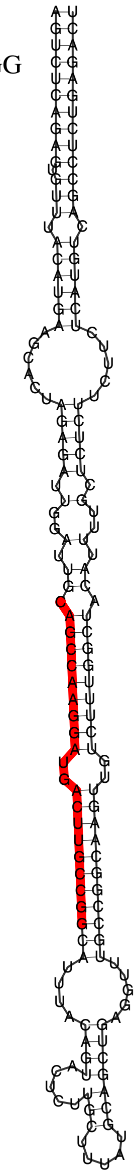

Name: ppe-miR169a

Sequence: CAGCCAAGGAUGACUUGCCGG

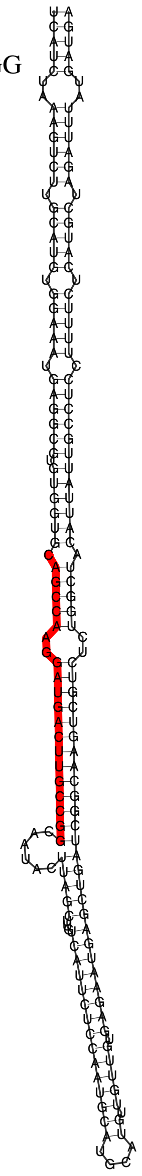

Name: ppe-miR169b

Sequence: UGAGCCAAGGAUGACUUGCCA

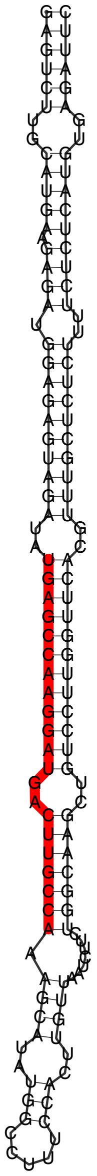

Name: ppe-miR169b

Sequence: UGAGCCAAGGAUGACUUGCCA

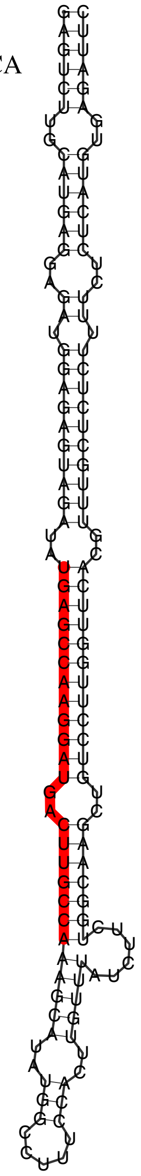

Name: ppe-miR169c

Sequence: UAGCCAAGGAUGACUUGCCUGC

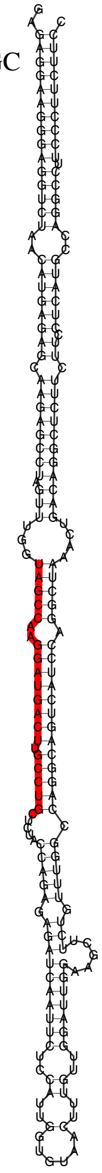

Name: ppe-miR169c

Sequence: UAGCCAAGGAUGACUUGCCUGC

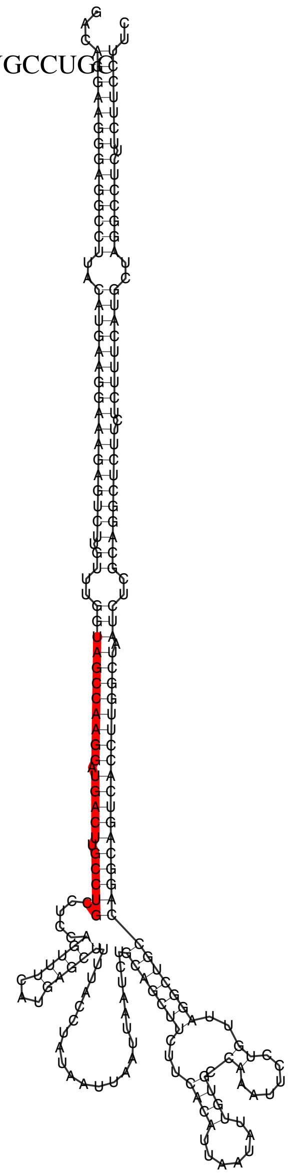

Name: ppe-miR169c

Sequence: UAGCCAAGGAUGACUUGCCUGC

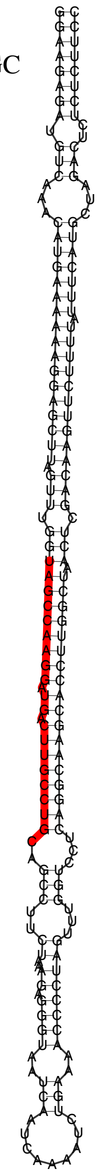

Name: ppe-miR169c

Sequence: UAGCCAAGGAUGACUUGCCUGC

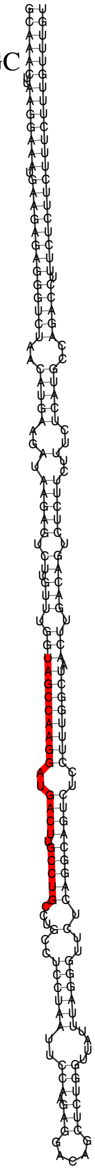

Name: ppe-miR169c

Sequence: UAGCCAAGGAUGACUUGCCUGC

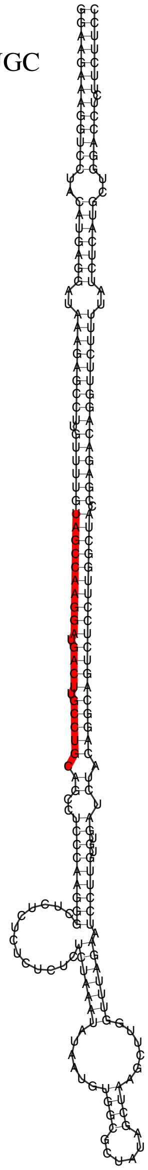

Name: ppe-miR169d

Sequence: GAGCCAAGGAUGAAUUGCCGG

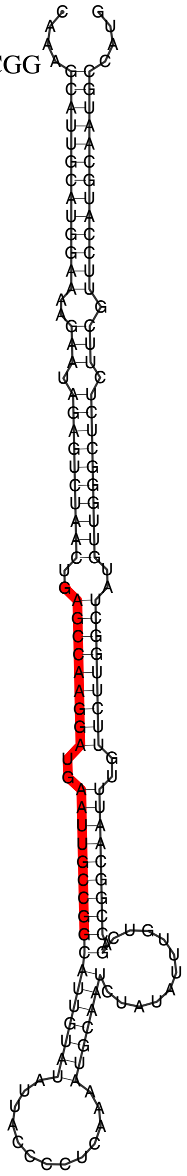

Name: ppe-miR169d

Sequence: GAGCCAAGGAUGAAUUGCCGG

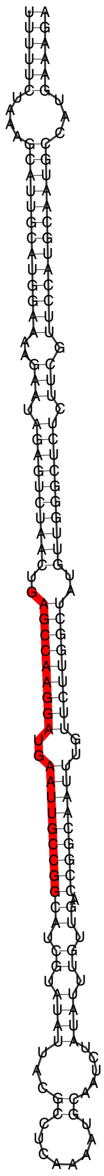

Name: ppe-miR171a

Sequence: CGAGCCGAAUCAAUAUCACUC

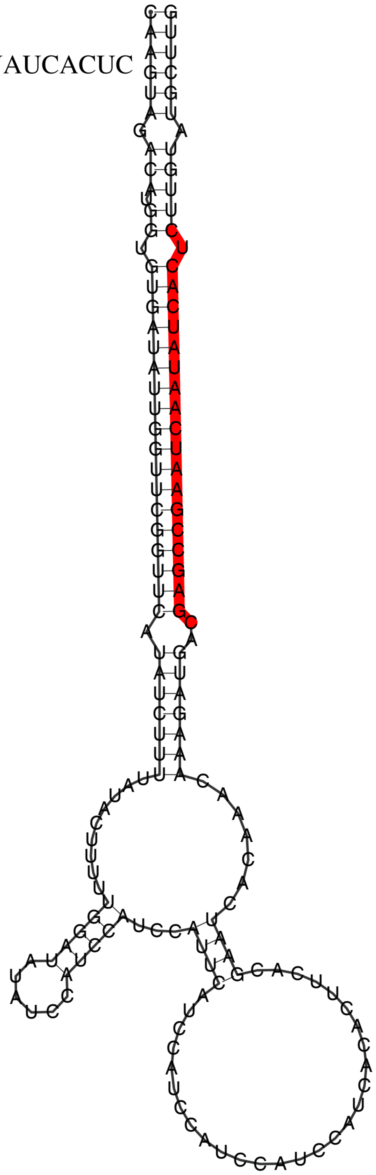

Name: ppe-miR171b

Sequence: UGAUUGAGCCGUGCCAAUAUC

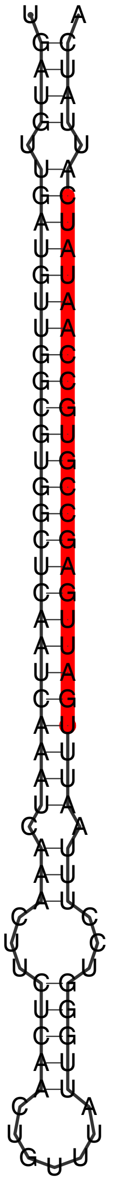

Name: ppe-miR171b

Sequence: UGAUUGAGCCGUGCCAAUAUC

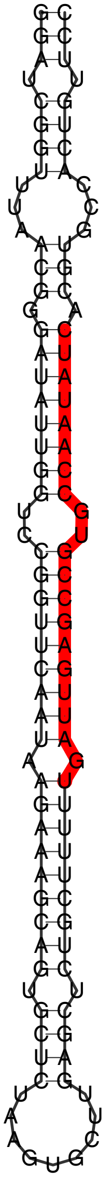

Name: ppe-miR171b

Sequence: UGAUUGAGCCGUGCCAAUAUC

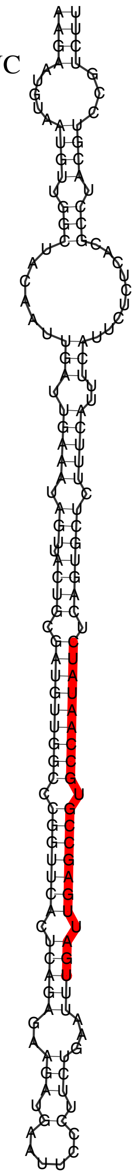

Name: ppe-miR171b

Sequence: UGAUUGAGCCGUGCCAAUAUC

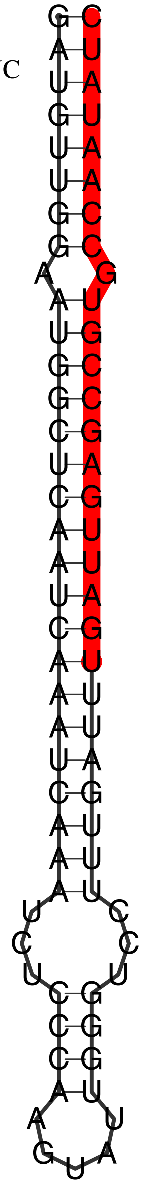

Name: ppe-miR171c

Sequence: UUGAGCCGCGUCAAUAUCUCC

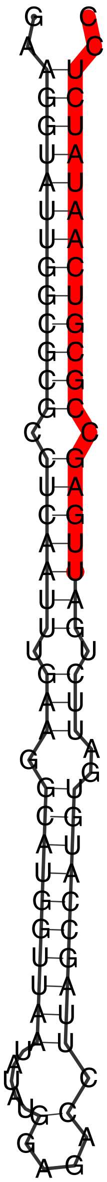

Name: ppe-miR172a

Sequence: GUAGCAUCAUCAAGAUUCACG

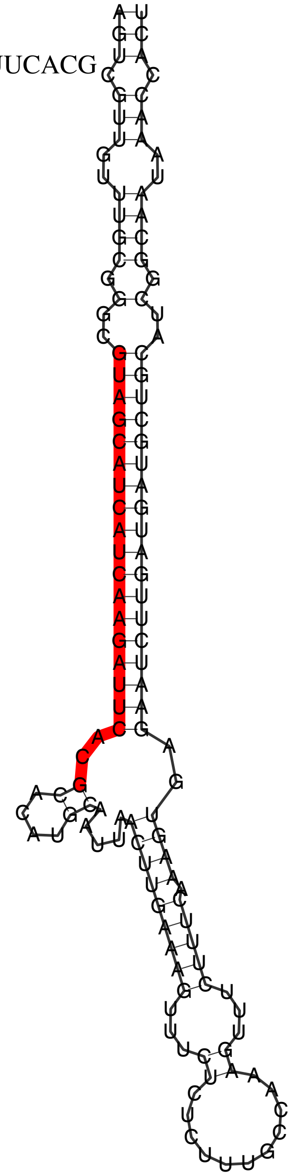

Name: ppe-miR172b

Sequence: GUAGCAUCAUCAAGAUUCAC

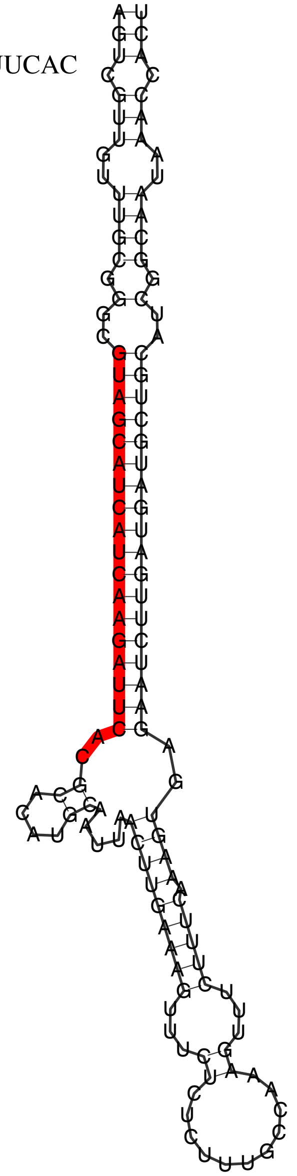

Name: ppe-miR172c

Sequence: AGAAUCUUGAUGAUGCUGCAU

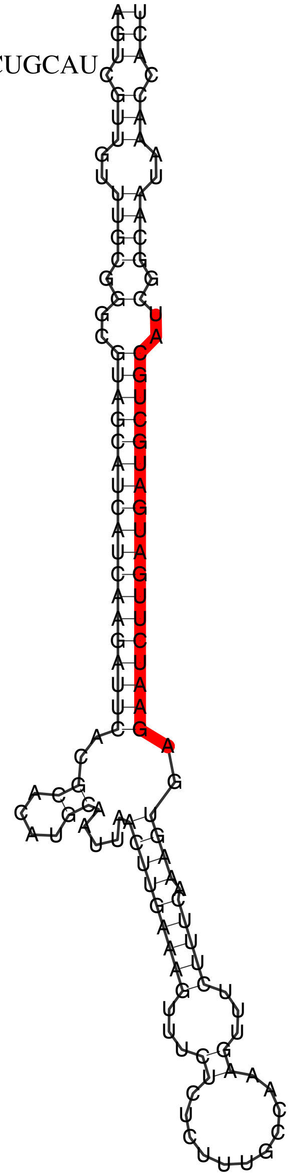

Name: ppe-miR172c

Sequence: AGAAUCUUGAUGAUGCUGCAU

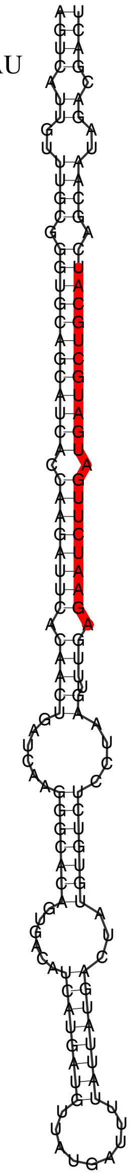

Name: ppe-miR172d

Sequence: GGAAUCUUGAUGAUGCUGCAU

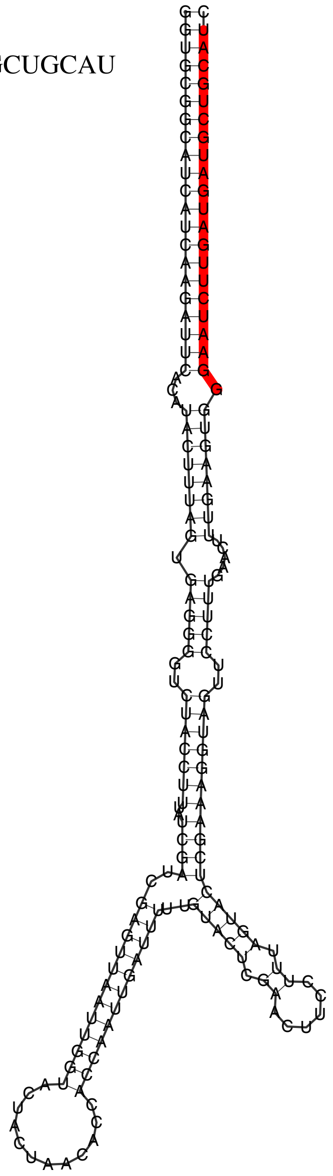

Name: ppe-miR172e

Sequence: GGAAUCUUGAUGAUGCUGCAG

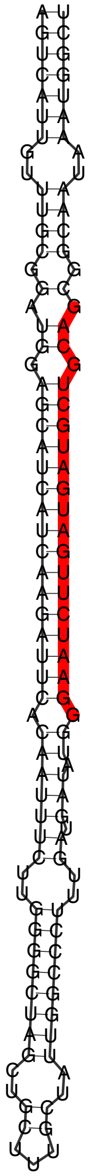

Name: ppe-miR390

Sequence: AAGCUCAGGAGGGAUAGCGCC

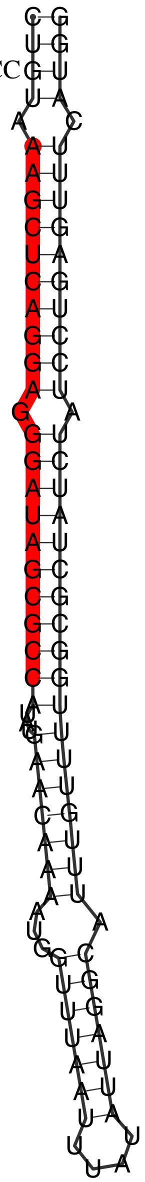

Name: ppe-miR393a

Sequence: CAUCCAAAGGGAUCGCAUUGA

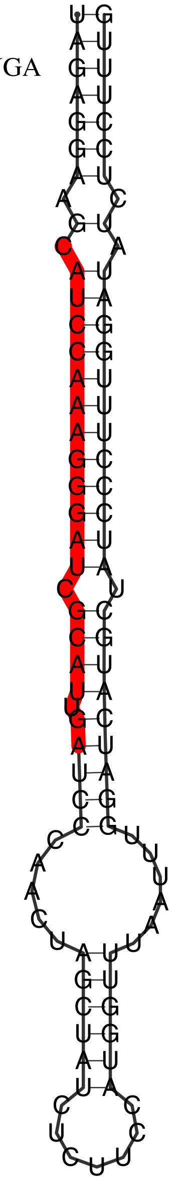

Name: ppe-miR393b

Sequence: UCCAAAGGGAUCGCAUUGAUC

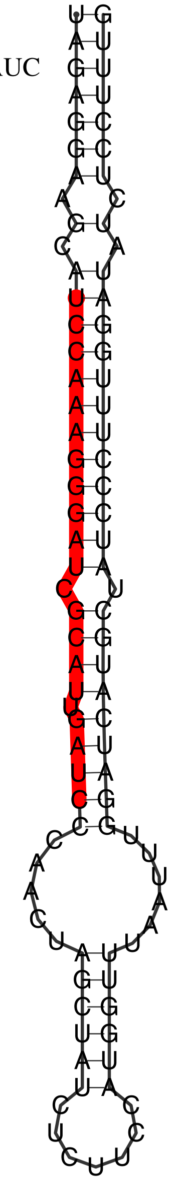

Name: ppe-miR393b

Sequence: UCCAAAGGGAUCGCAUUGAUC

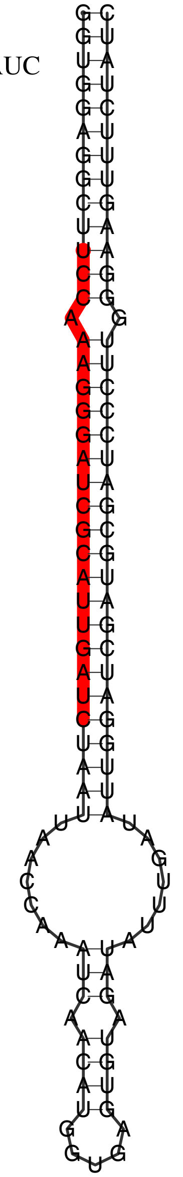

Name: ppe-miR394a

Sequence: UUGGCAUUCUGUCCACCUCC

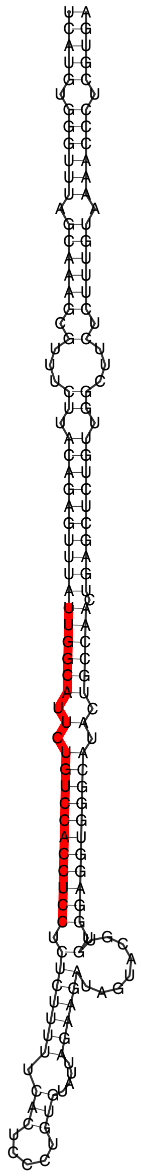

Name: ppe-miR394a

Sequence: UUGGCAUUCUGUCCACCUCC

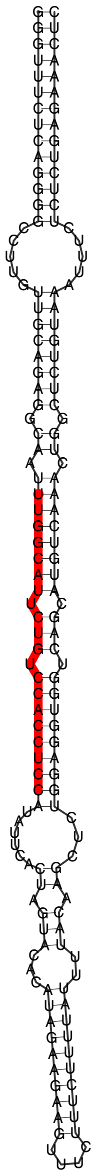

Name: ppe-miR394b

Sequence: UUUGGCAUUCUGUCCACCUCC

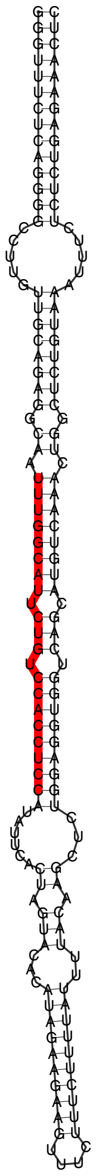

Name: ppe-miR395a

Sequence: CUGAAGUGUUUGGGGGACCC

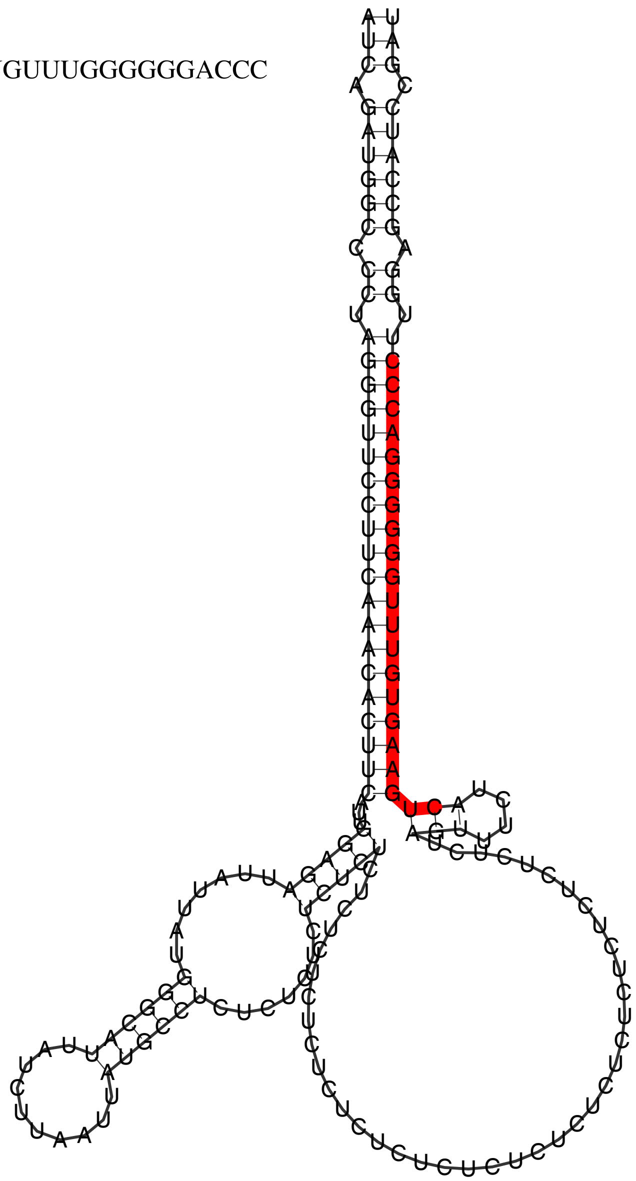

Name: ppe-miR395a

Sequence: CUGAAGUGUUUGGGGGGACCC

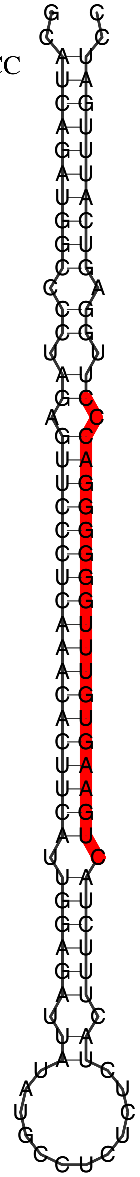

Name: ppe-miR395a

Sequence: CUGAAGUGUUUGGGGGGACCC

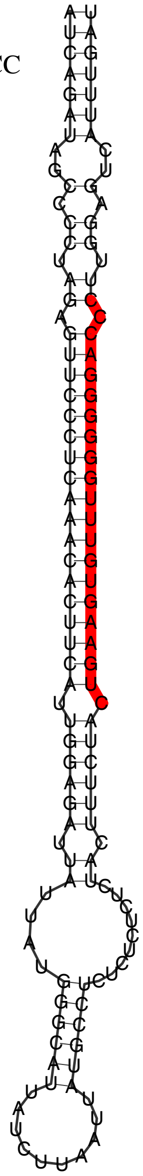

Name: ppe-miR395b

Sequence: CUGAAGUGUUUGGGGGAACUC

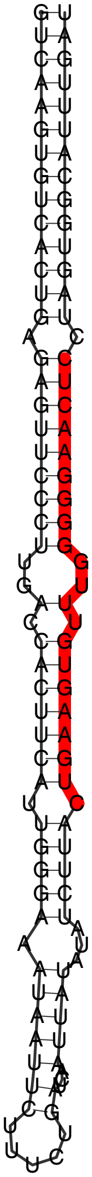

Name: ppe-miR395b

Sequence: CUGAAGUGUUUGGGGGAACUC

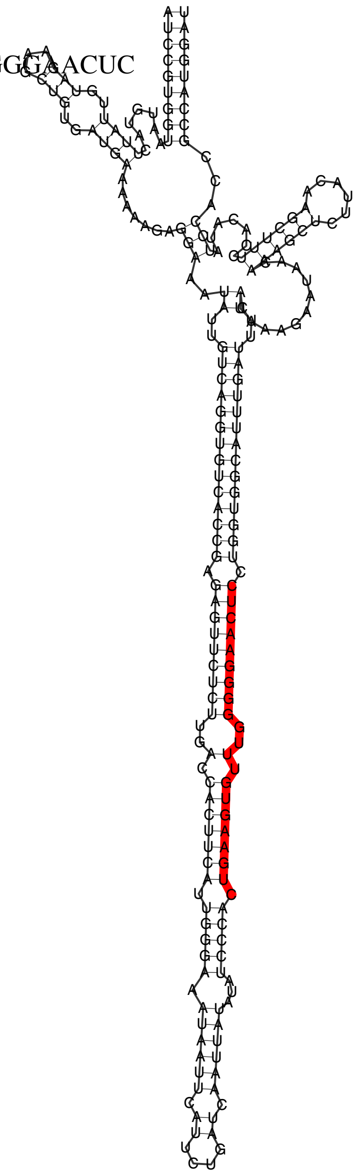

Name: ppe-miR395b

Sequence: CUGAAGUGUUUGGGGGAACUC

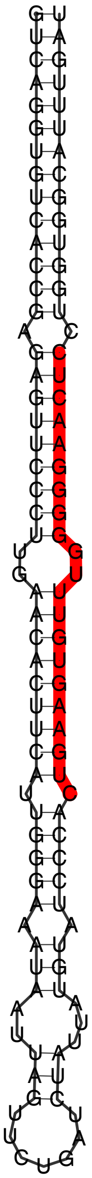

Name: ppe-miR395b

Sequence: CUGAAGUGUUUGGGGGAACUC

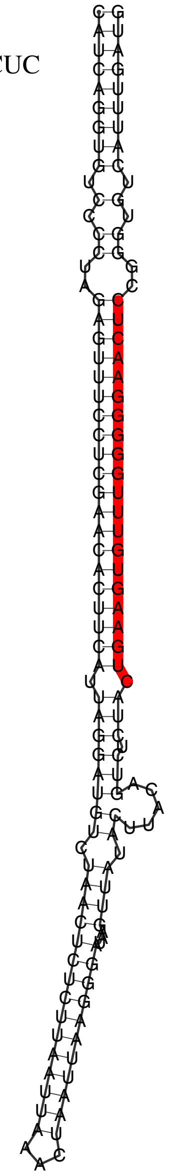

Name: ppe-miR395b

Sequence: CUGAAGUGUUUGGGGGAACUC

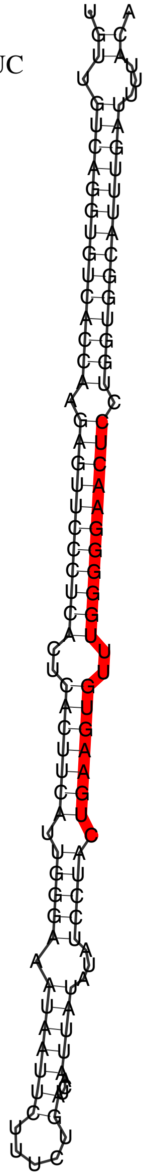

Name: ppe-miR395b

Sequence: CUGAAGUGUUUGGGGGAACUC

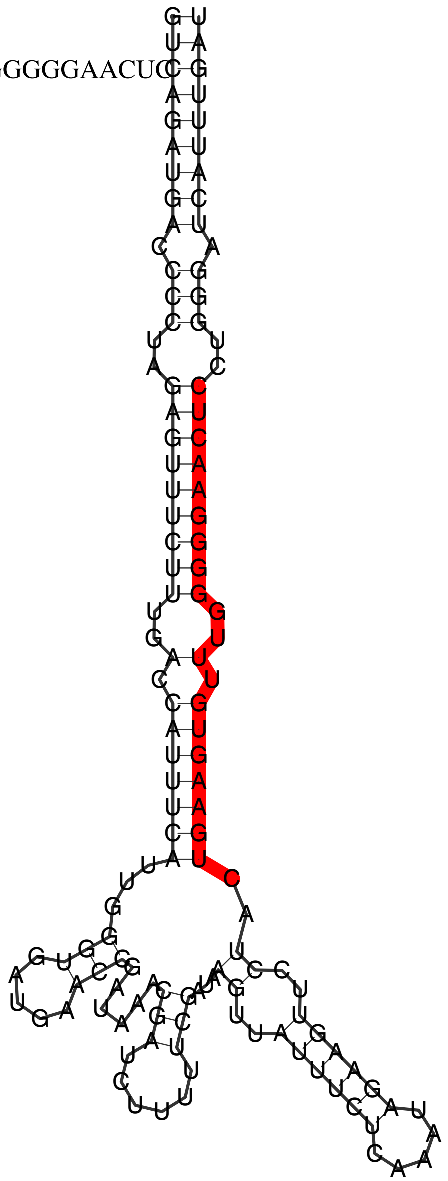

Name: ppe-miR395b

Sequence: CUGAAGUGUUUGGGGGAACUC

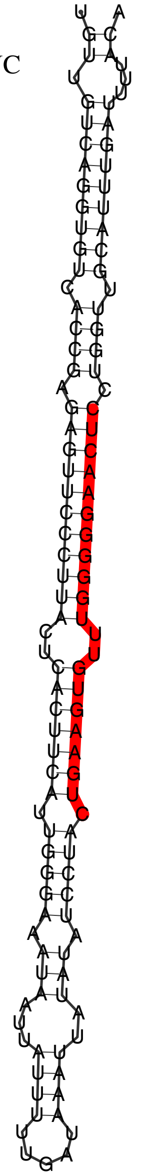

Name: ppe-miR395b

Sequence: CUGAAGUGUUUGGGGGAACUC

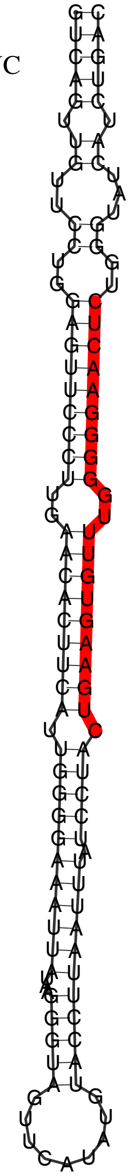

Name: ppe-miR395b

Sequence: CUGAAGUGUUUGGGGGAACUC

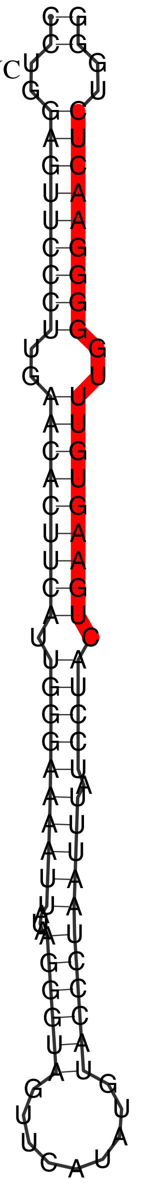

Name: ppe-miR395b

Sequence: CUGAAGUGUUUGGGGGAACUC

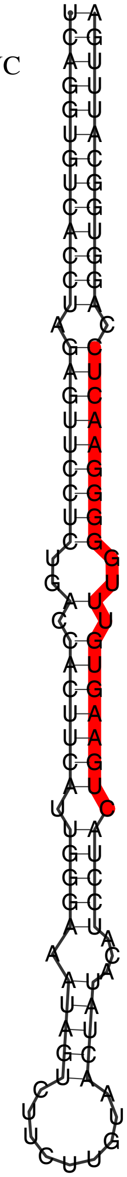

Name: ppe-miR395b

Sequence: CUGAAGUGUUUGGGGGAACUC

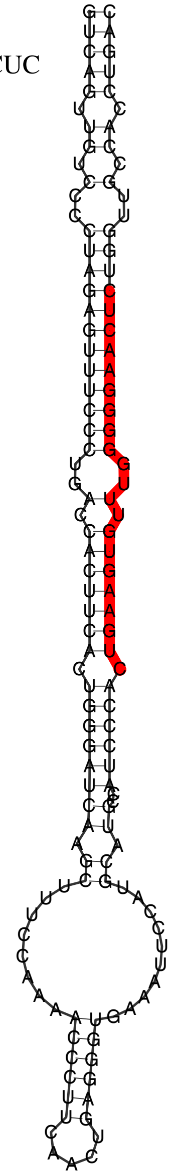

Name: ppe-miR395b

Sequence: CUGAAGUGUUUGGGGGAACUC

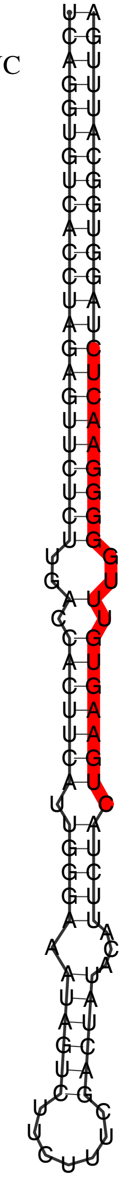

Name: ppe-miR395c

Sequence: GUUCCCUCAAACACUUCAUU

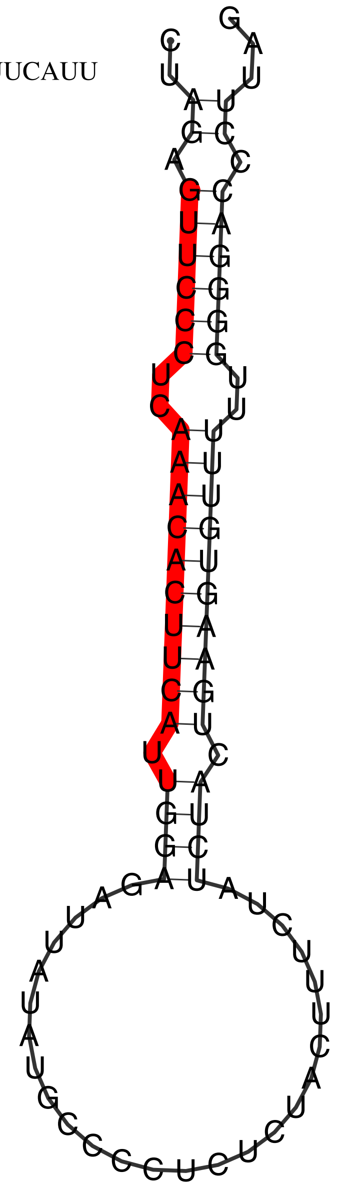

Name: ppe-miR395d

Sequence: GUUCCUUCAAACACUUCAUU

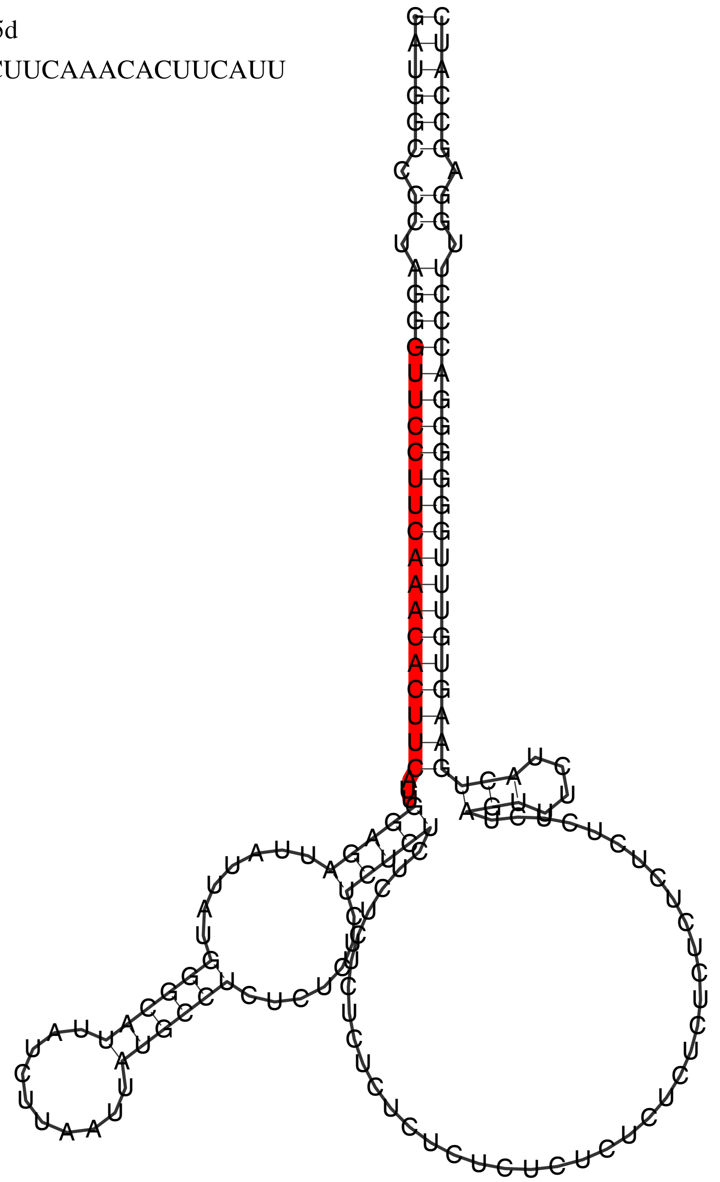

Name: ppe-miR396a

Sequence: UUCCACAGCUUUCUUGAACGU

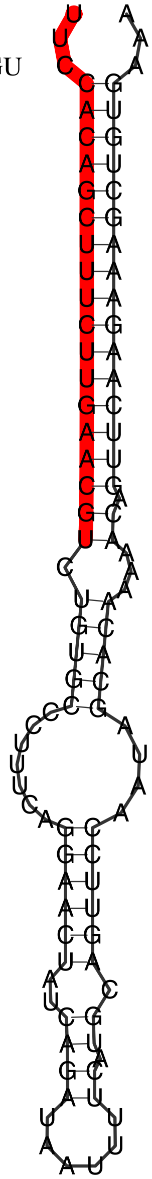

Name: ppe-miR396b

Sequence: UUCCACAGCUUUCUUGAACUG

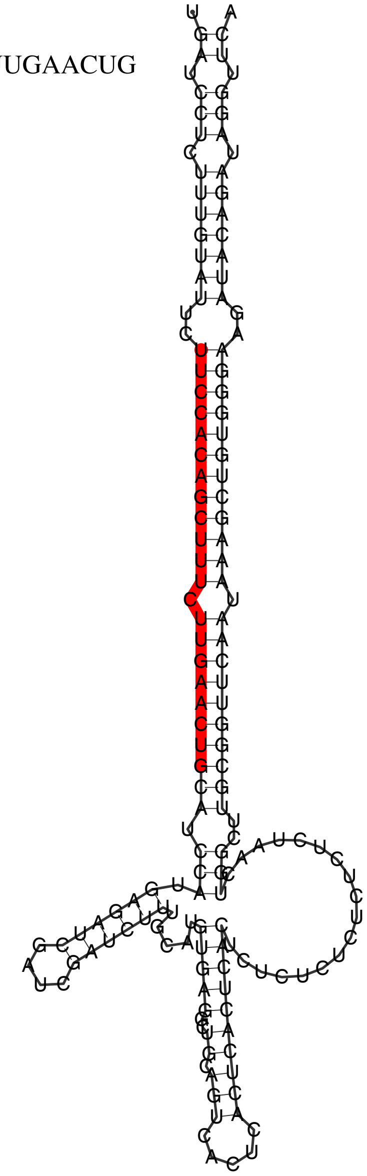

Name: ppe-miR396c

Sequence: UUCCACAGCUUUCUUGAACUU

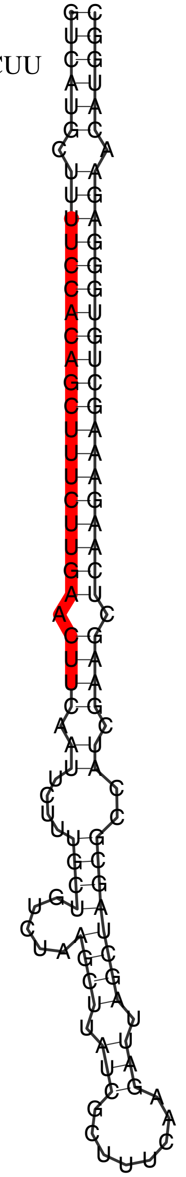

Name: ppe-miR397

Sequence: UCAUUGAGUGCAGCGUUGAUG

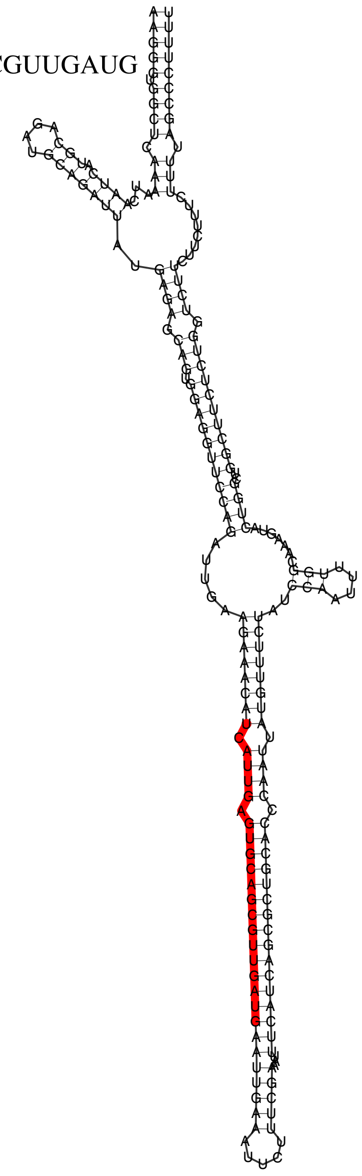

Name: ppe-miR398a

Sequence: UGUGUUCUCAGGUCGCCCCUG

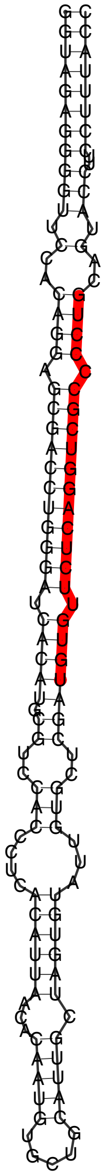

Name: ppe-miR398b

Sequence: CGUGUUCUCAGGUCGCCCCUG

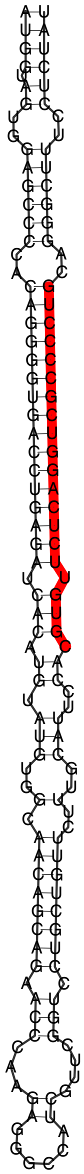

Name: ppe-miR399a

Sequence: CGCCAAAGGAGAGUUGCCCUU

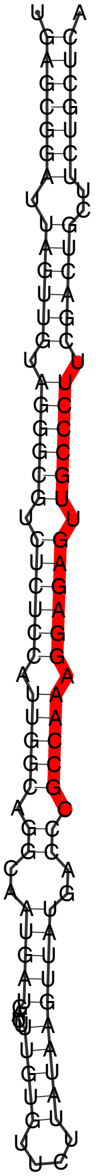

Name: ppe-miR399b

Sequence: UCUGCCAAAGGAGAAUUGCCC

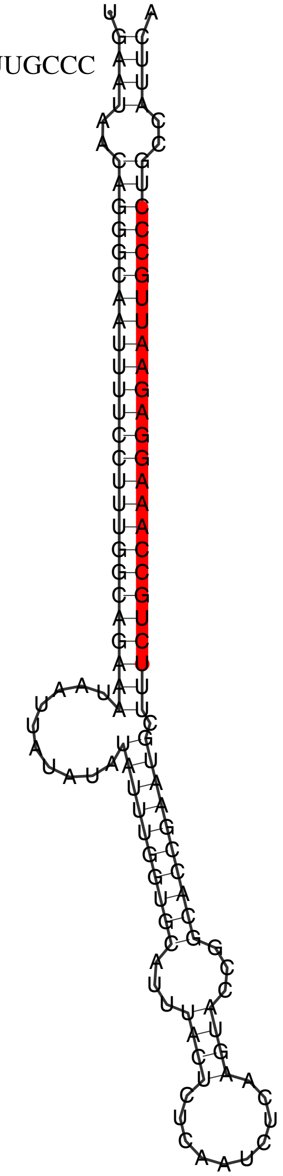

Name: ppe-miR399c

Sequence: UGCCAAAGAAGAGUUGCCCUA

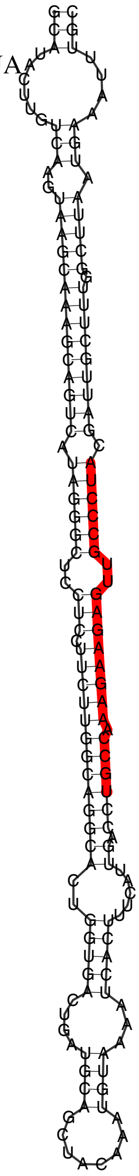

Name: ppe-miR399c

Sequence: UGCCAAAGAAGAGUUGCCCUA

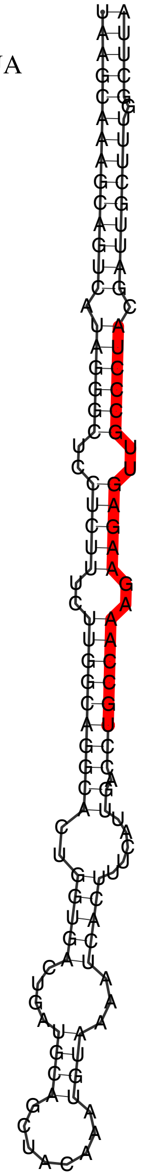

Name: ppe-miR399c

Sequence: UGCCAAAGAAGAGUUGCCCUA

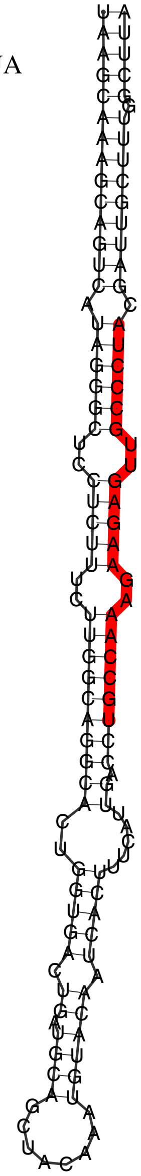

Name: ppe-miR399c

Sequence: UGCCAAAGAAGAGUUGCCCUA

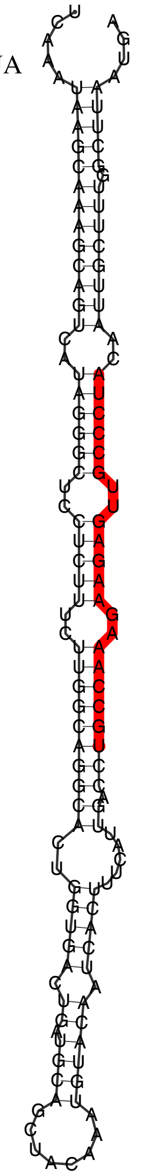

Name: ppe-miR399c

Sequence: UGCCAAAGAAGAGUUGCCCUA

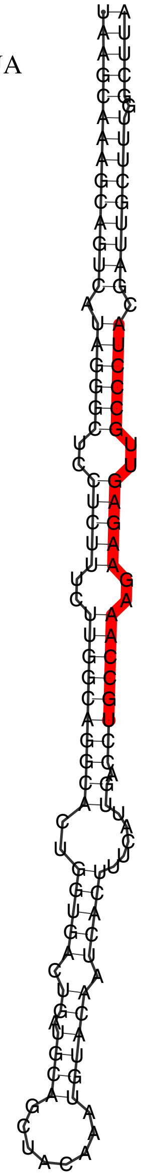

Name: ppe-miR399c

Sequence: UGCCAAAGAAGAGUUGCCCUA

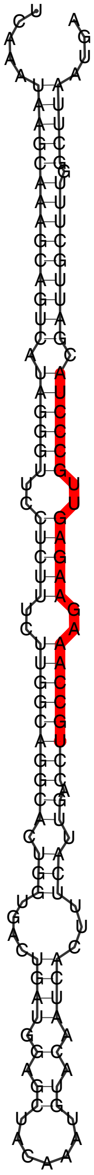

Name: ppe-miR399c

Sequence: UGCCAAAGAAGAGUUGCCCUA

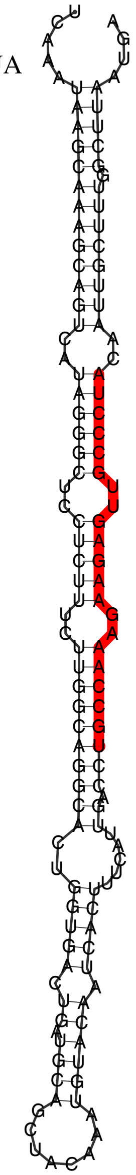

Name: ppe-miR399c

Sequence: UGCCAAAGAAGAGUUGCCCUA

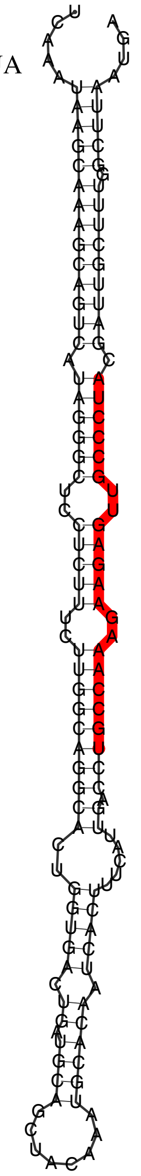

Name: ppe-miR399c

Sequence: UGCCAAAGAAGAGUUGCCCUA

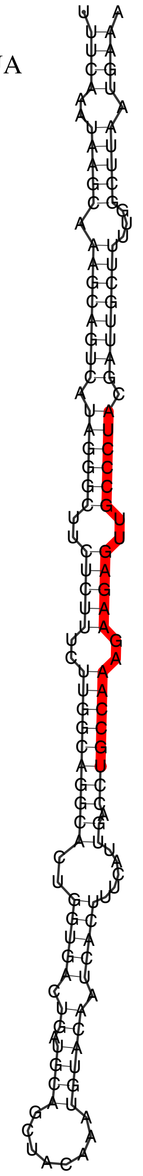

Name: ppe-miR399c

Sequence: UGCCAAAGAAGAGUUGCCCUA

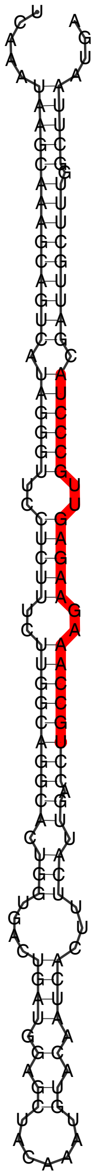

Name: ppe-miR399d

Sequence: UGCCAAAGGAGAUUUGCUCGG

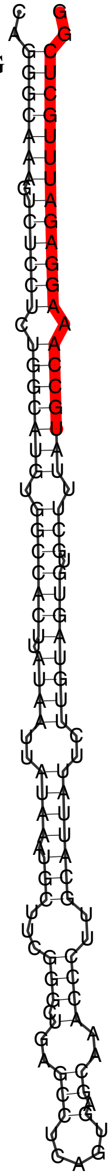

Name: ppe-miR399d

Sequence: UGCCAAAGGAGAUUUGCUCGG

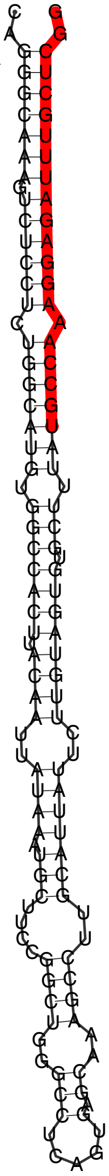

Name: ppe-miR403

Sequence: UUAGAUUCACGCACAAACUCG

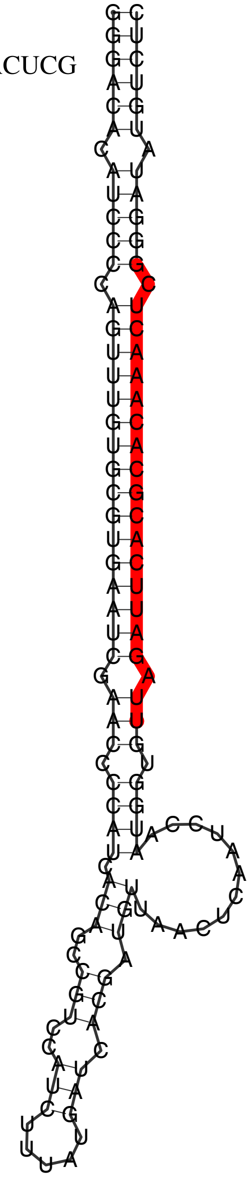

Name: ppe-miR479

Sequence: UGUGAUAUUGGUUCGGUUCAUA

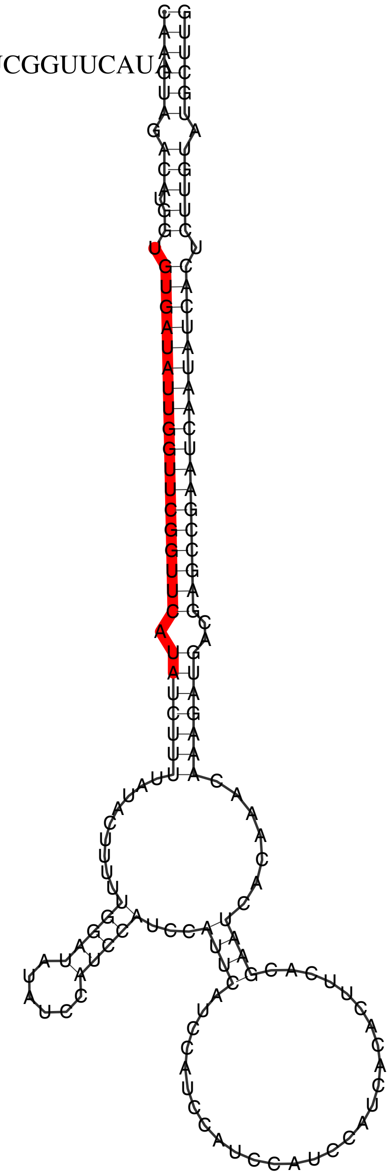

Name: ppe-miR482a

Sequence: UUCCCAAGCCCGCCCAUUCCAA

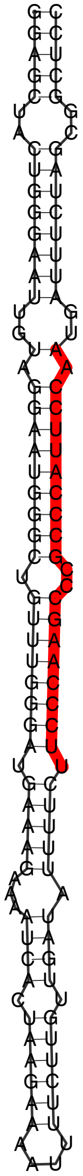

Name: ppe-miR482b

Sequence: UCUUUCCUACUCCACCCAUUCC

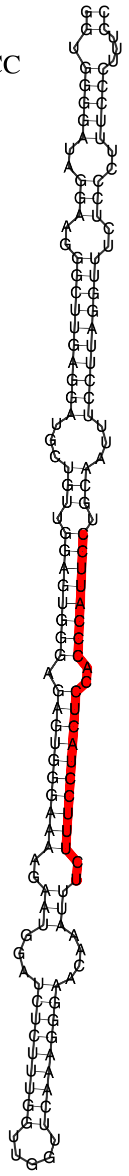

Name: ppe-miR530

Sequence: UCUGCAUUUGCACCUGCACCU

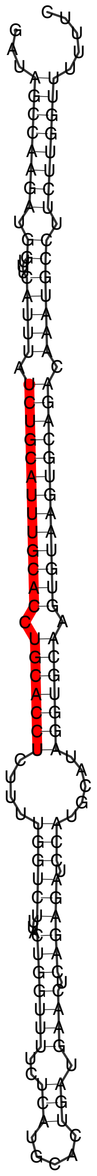

Name: ppe-miR535a

Sequence: UGACAACGAGAGAGAGCACGC

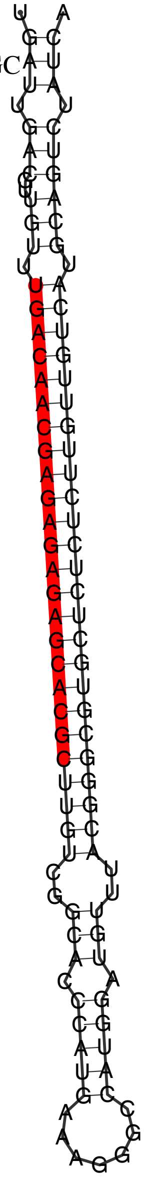

Name: ppe-miR535b

Sequence: UGACGACGAGAGAGAGCACGC

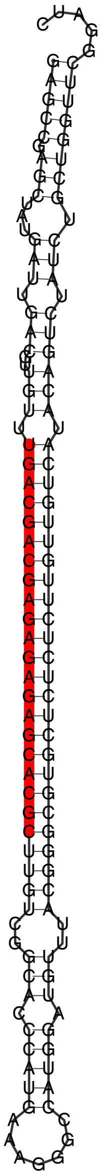

Name: ppe-miR827

Sequence: UUAGAUGACCAUCAACAAACA

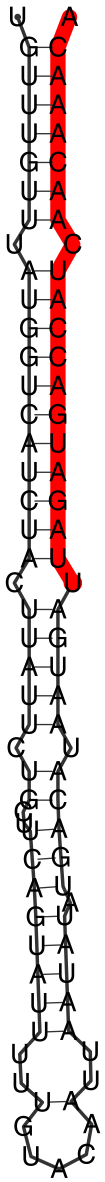

Name: ppe-miR828

Sequence: UCUUGCUCAAAUGAGUAUUCCA

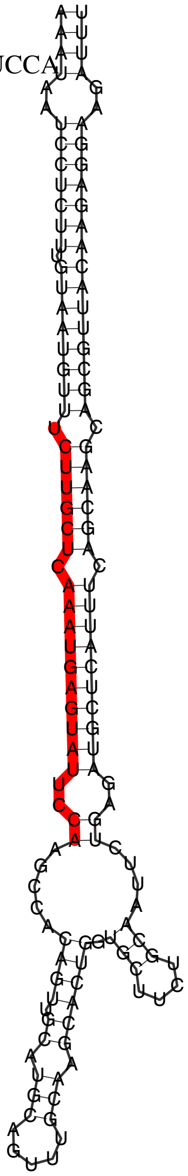

Name: ppe-miR2111

Sequence: UAAUCUGCAUCCUGAGGUUUA

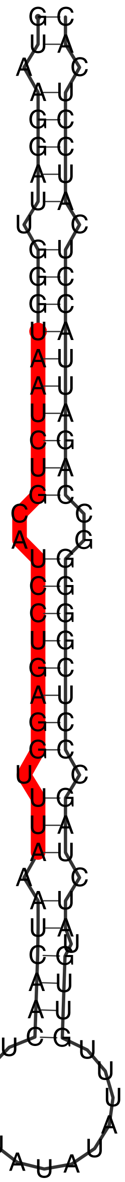

Name: ppe-miR2111

Sequence: UAAUCUGCAUCCUGAGGUUUA

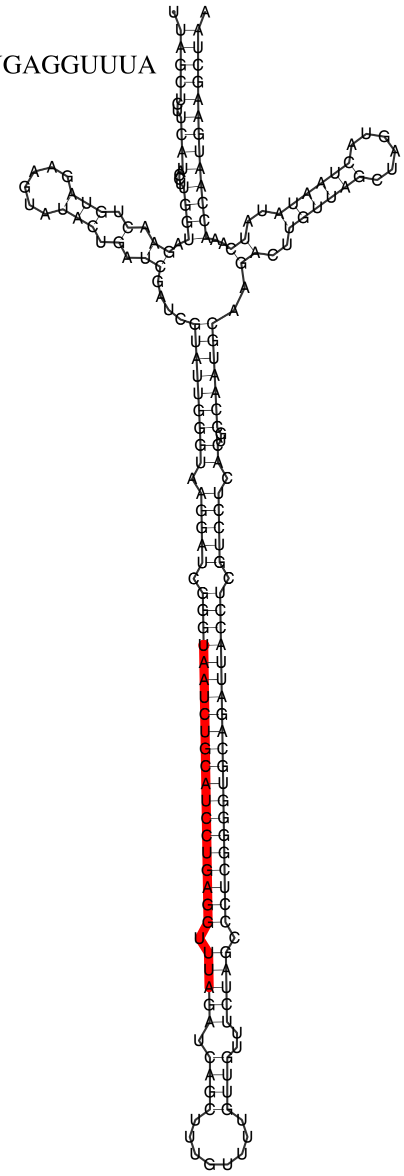

Name: ppe-miR2111

Sequence: UAAUCUGCAUCCUGAGGUUUA

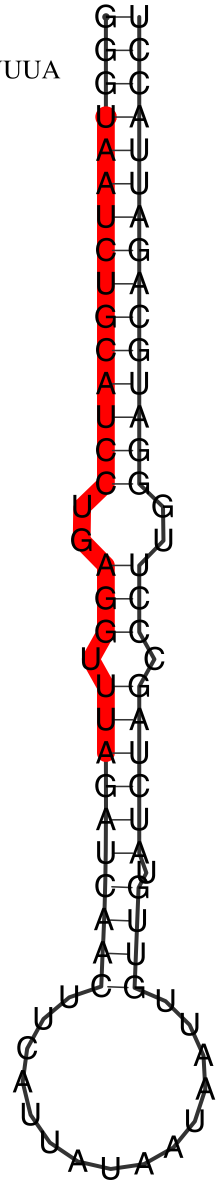

Name: ppe-miR2111

Sequence: UAAUCUGCAUCCUGAGGUUUA

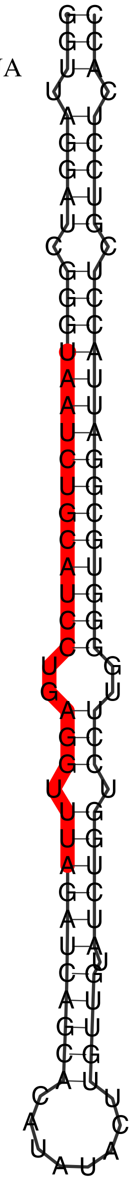

Name: miRC1

Sequence: ACCUGGCUCUGAUACCAUAAC

Abundance: 23436

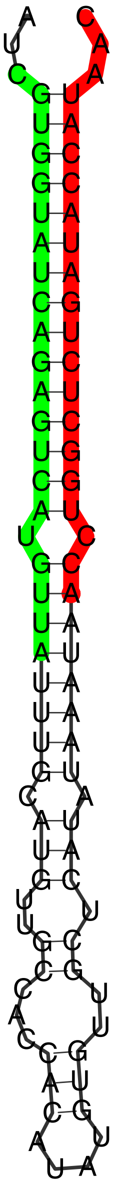

Name: miRC2

Sequence: UGAAGGAAGAUUUGUGGAAAG

Abundance: 5705

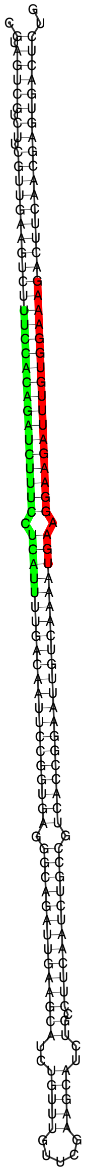

Name: miRC3

Sequence: CUUCCCAAACCUCCCAUUCCUA

Abundance: 4165

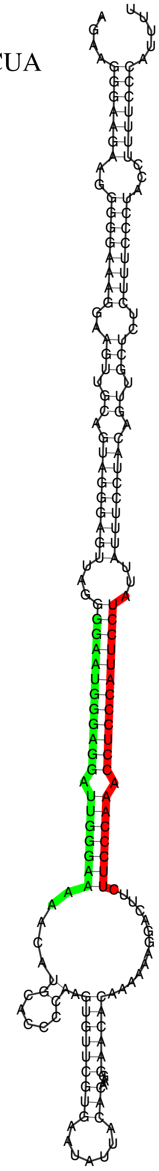

Name: miRC4

Sequence: UGAGCAAUGGCACACAGCCCU

Abundance: 3110

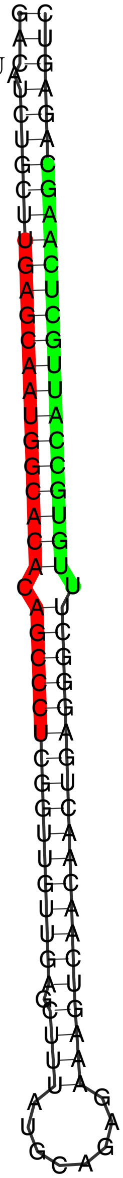

Name: miRC5

Sequence: UUUCCGAAACCUCCCAUUCCAA

Abundance: 2658

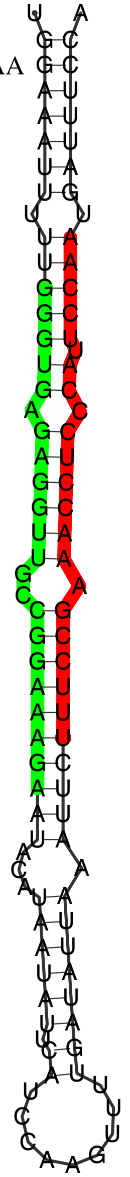

Name: miRC6a

Sequence: UUAUACAAUGAAAUCACGGCCG

Abundance: 2172

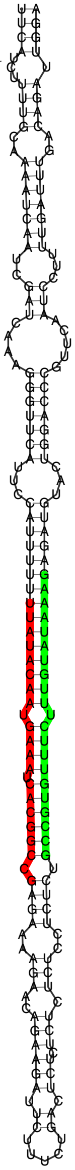

Name: miRC6b

Sequence: UUAUACAAUGAAAUCACGGUCG

Abundance: 180

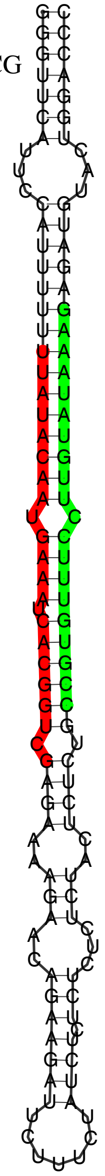

Name: miRC7

Sequence: UGGCACCAAUGAUACCAAGUUU

Abundance: 1390

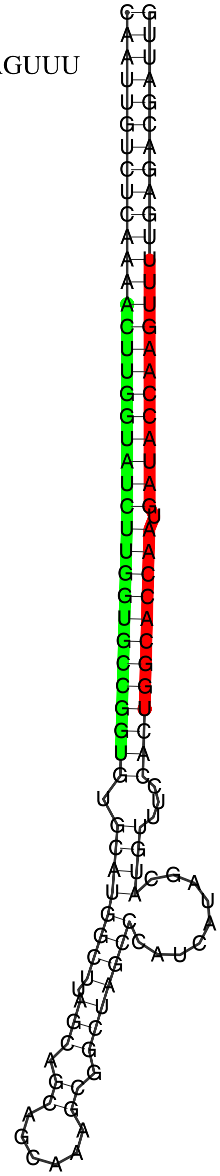

Name: miRC8

Sequence: CAGGAAAGAAUGUGAUGAGUA

Abundance: 504

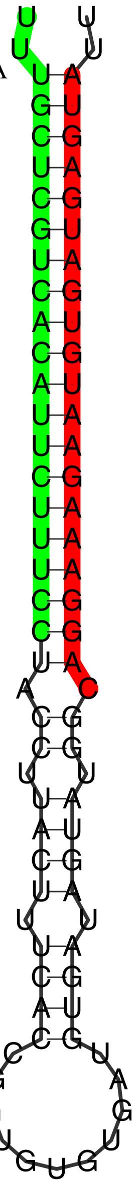

Name: miRC9

Sequence: UCGCAGGAGAGAUGGCACUGUC

Abundance: 125

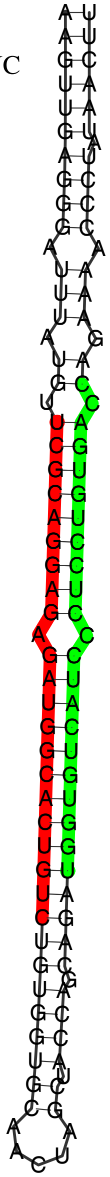

Name: miRC10

Sequence: CGAACUUAUUGCAACUAGCUU

Abundance: 78

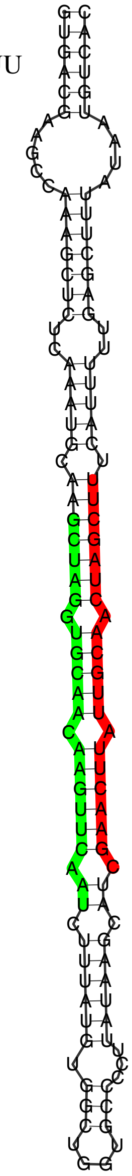

Name: miRC11

Sequence: GGAGCGACCUGGGAUCACAUG

Abundance: 49

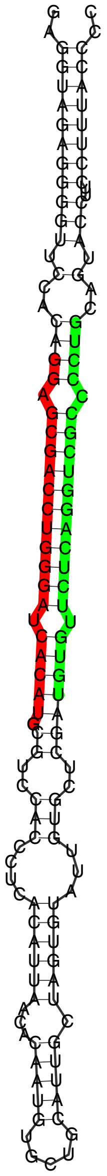

Name: miRC12

Sequence: UCUGAGUCAGAUUACUGAAUA

Abundance: 43

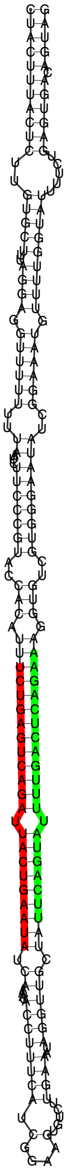

Name: miRC13

Sequence: ACUCUCCCUCAAAGGCUUCUAG

Abundance: 37

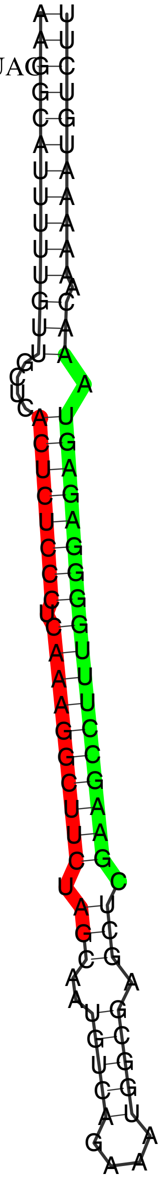

Name: miRC14

Sequence: UAGAGAGAUGGUCAGCAAUGU

Abundance: 33

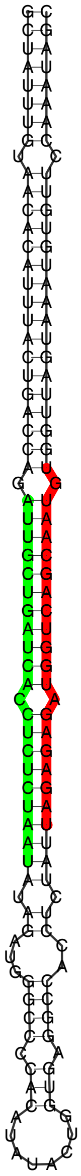

Name: miRC15

Sequence: CCACAUUUAUAGAUUACCUUG

Abundance: 29

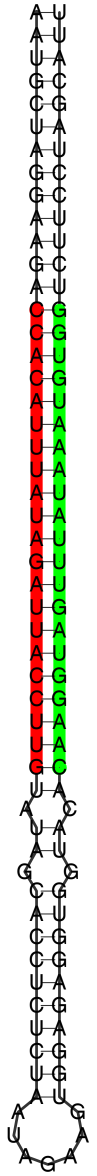

Name: miRC16

Sequence: UUCAAAGGGUACAUCCACAGU

Abundance: 22

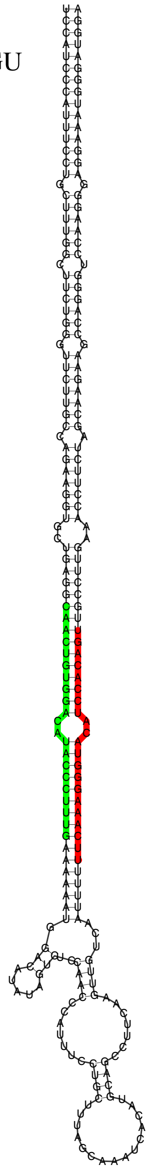

Name: miRC17

Sequence: UCUGUCGUAGGAGAGAUGGCCC

Abundance: 21

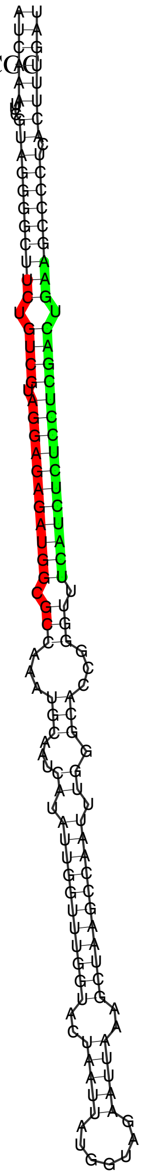

Name: miRC18

Sequence: UCGUGGGGAGAGAUCUAAUCG

Abundance: 18

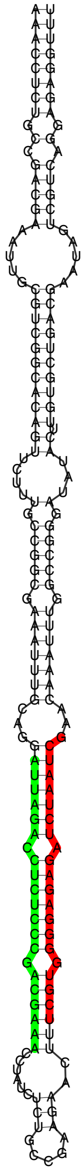

Name: miRC19

Sequence: CCUCCCAUGCCACGCAUUUCUA

Abundance: 17

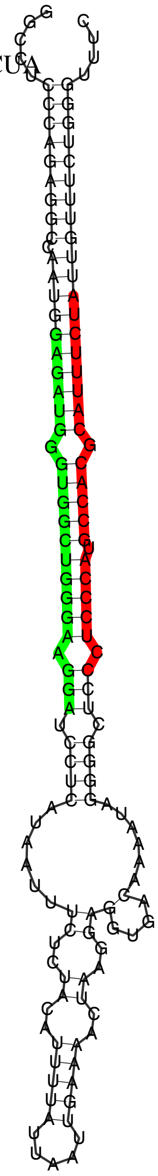

Name: miRC20

Sequence: AUUUCGACUAAUAACACAAUG

Abundance: 16

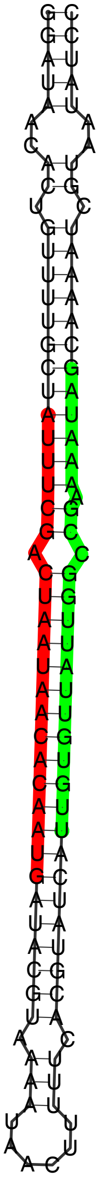

Name: miRC21

Sequence: AUAAUAAUGUCCGGAUGUCAA

Abundance: 11

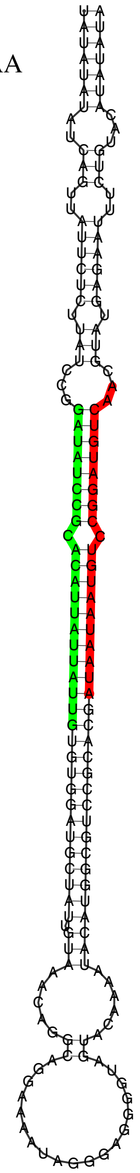

Name: miRC22

Sequence: CCCUUCCAGUAAGGCACCCCC

Abundance: 11

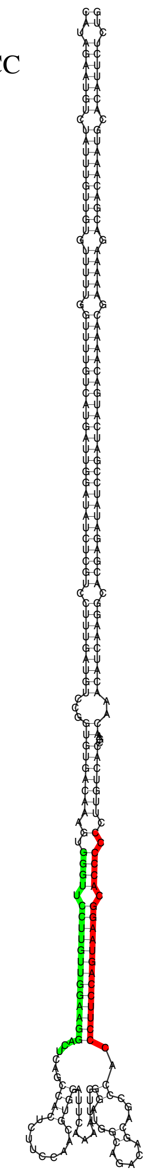

Name: miRC23

Sequence: AUUUCAGCUAAGUUGAGUUGU

Abundance: 10

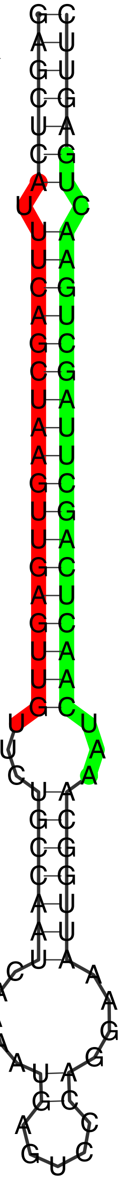

Name: miRC24

Sequence: UCCCUCAAGGGCUCCCAAUAUU

Abundance: 10

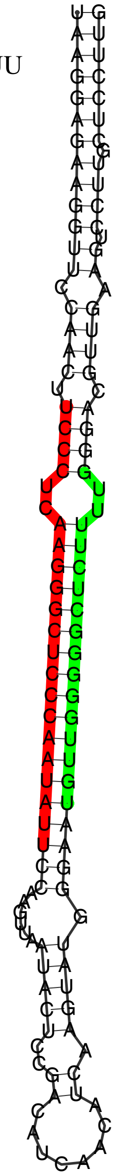

Name: miRC25

Sequence: UCAAUUAGAAAAUGAUAAGUG

Abundance: 7

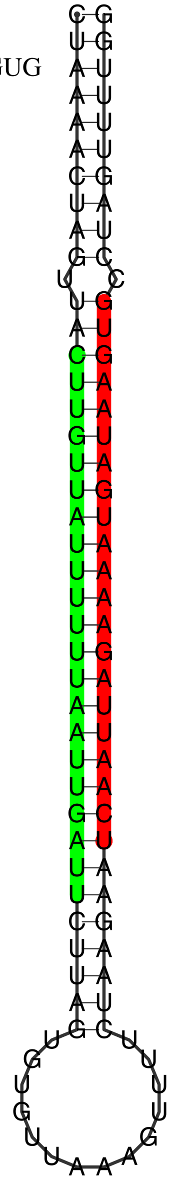

Name: miRC26

Sequence: UCCAACGAUGGGUGACCACAA

Abundance: 7

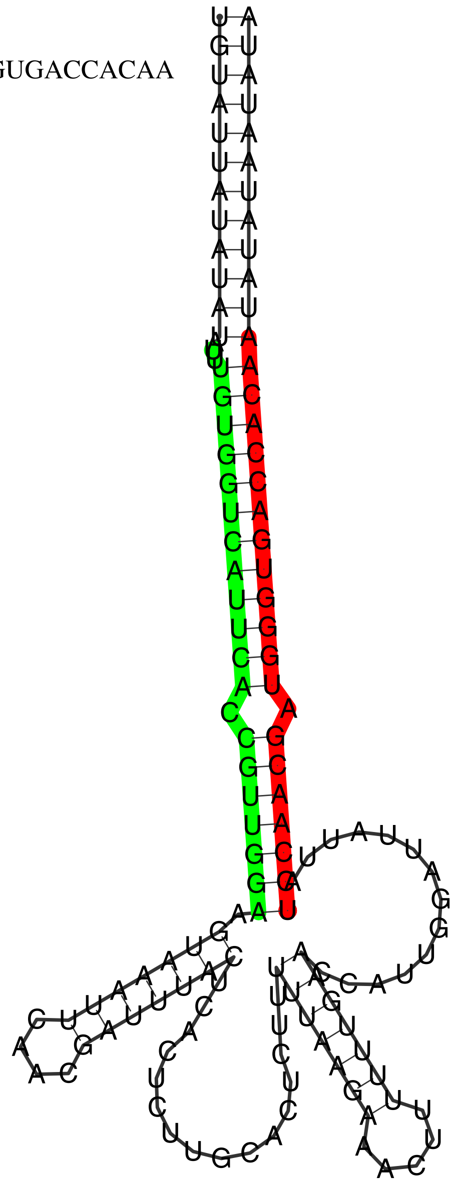

Name: miRC27

Sequence: UCCUGUGCGAACGUCCAGAAG

Abundance: 7

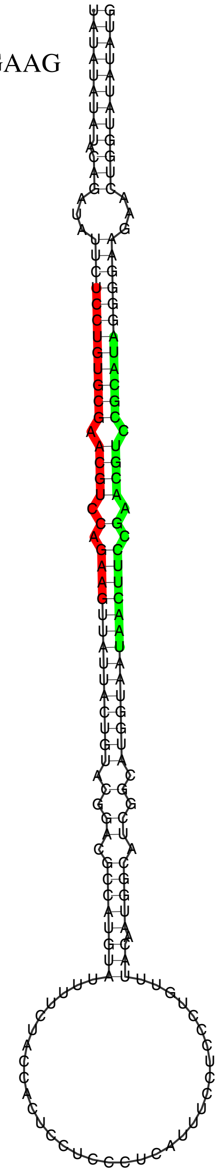

Name: miRC28

Sequence: CUUGUUAUUUUUUAAUUGAUU

Abundance: 5

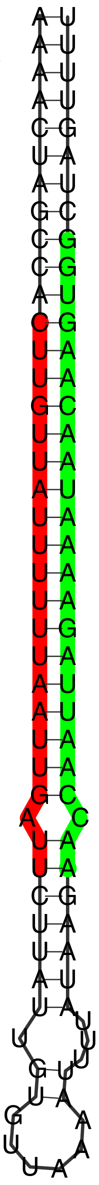

Name: miRC29

Sequence: AAAGACUAAAAUACCCUUGA

Abundance: 60

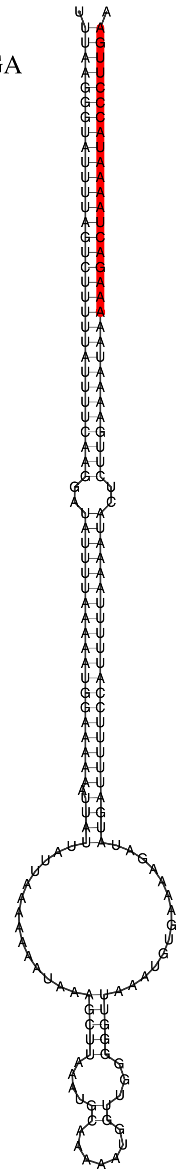

Name: miRC29

Sequence: AAAGACUAAAAUACCCUUGA

Abundance: 60

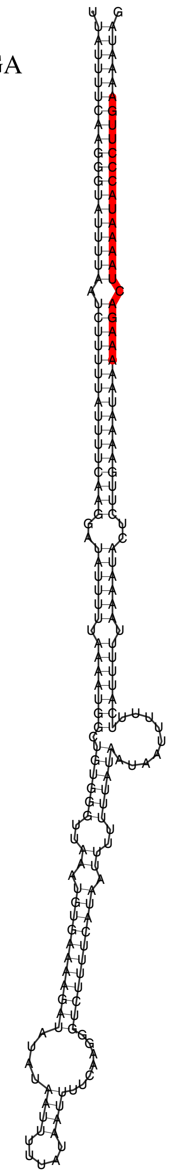

Name: miRC30

Sequence: UACUUGACCCCACAACUGGUU

Abundance: 44

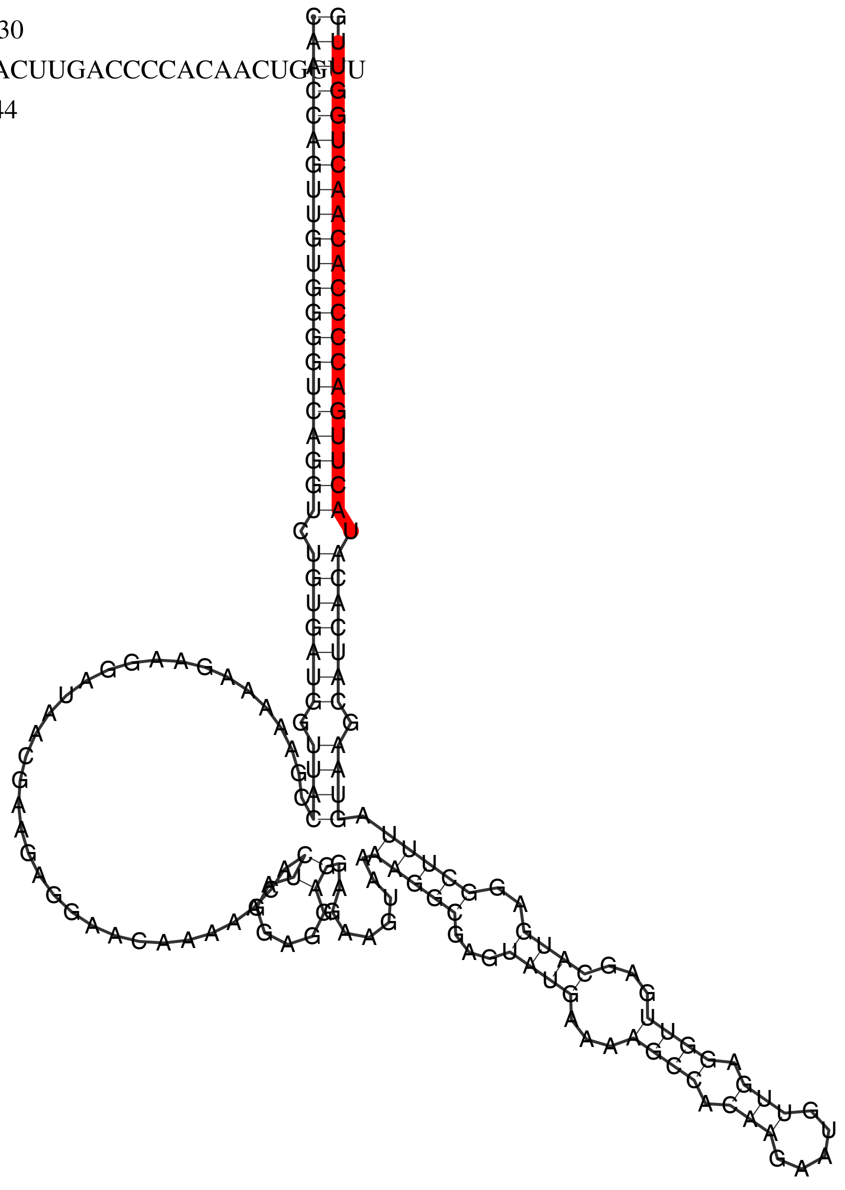

Name: miRC31

Sequence: UGGGCACGCCAGAAUAAAGCAA

Abundance: 39

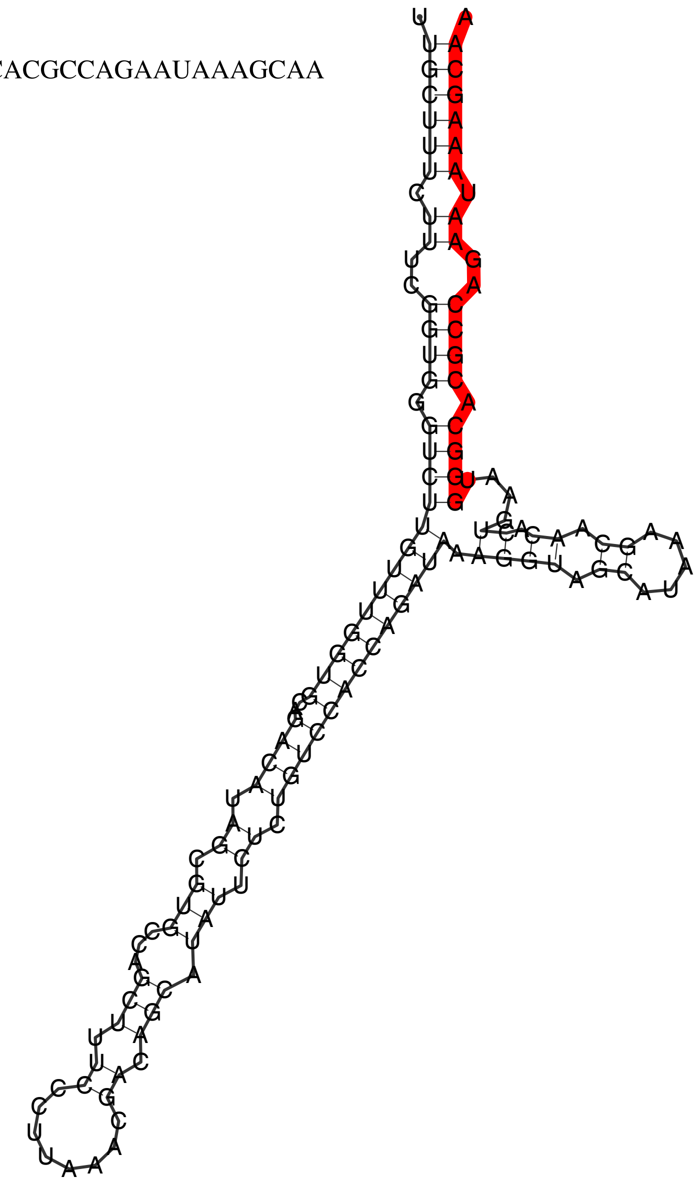

Name: miRC32

Sequence: UAAGGUUGAGCCGGAAAUCGGA

Abundance: 26

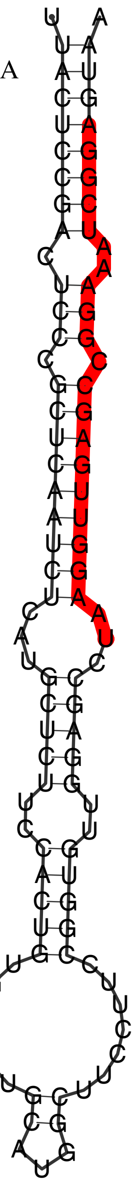

Name: miRC33

Sequence: CUCUUAAUCGUUGGAUCAAAUU

Abundance: 22

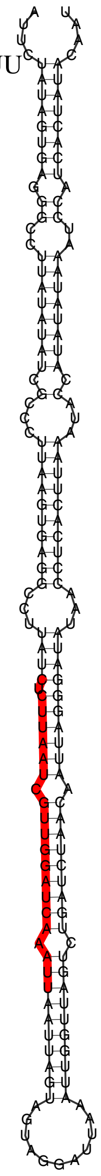

Name: miRC33

Sequence: CUCUUAAUCGUUGGAUCAAAUU

Abundance: 22

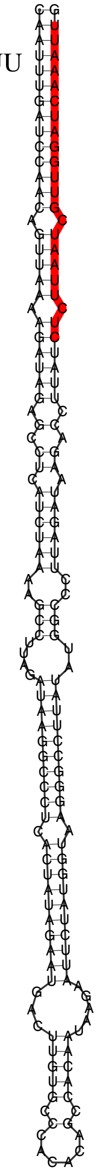

Name: miRC33

Sequence: CUCUUAAUCGUUGGAUCAAAUU

Abundance: 22

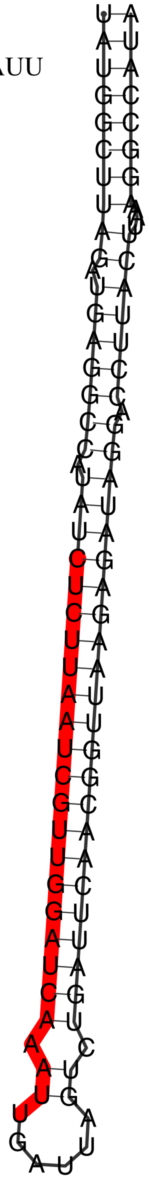

Name: miRC34

Sequence: UGCUUGUUGAGAUGUGCGGUU

Abundance: 19

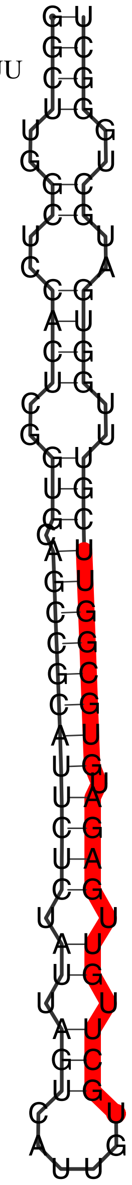

Name: miRC35

Sequence: UGUGUUAAUCGUAGAAAAUAU

Abundance: 17

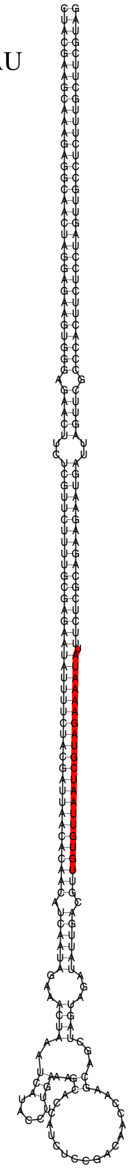

Name: miRC36

Sequence: AAUGUCACCUCCCACACUCCU

Abundance: 16

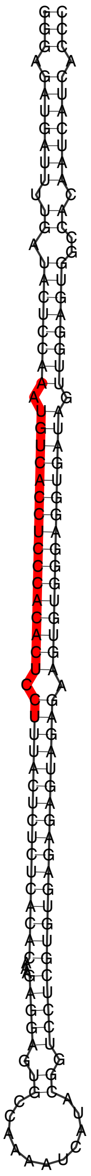

Name: miRC37

Sequence: UGGACGUCUAGAAAAAUACGG

Abundance: 15

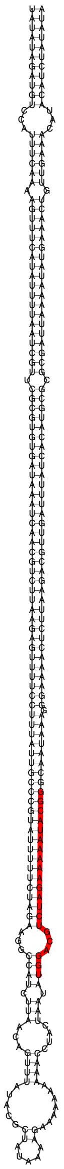

Name: miRC38

Sequence: UUAAGCCCAAGAAAGCCCGAC

Abundance: 14

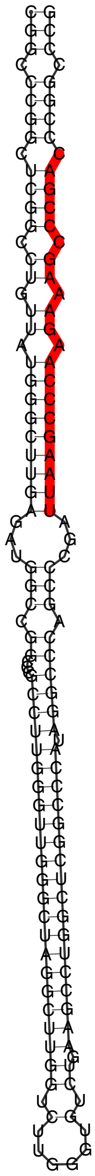

Name: miRC39

Sequence: ACCUCUUAUAGAUAGUCCCCA

Abundance: 12

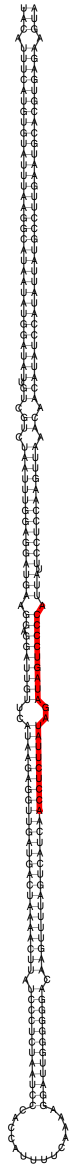

Name: miRC39

Sequence: ACCUCUUAUAGAUAGUCCCCA

Abundance: 12

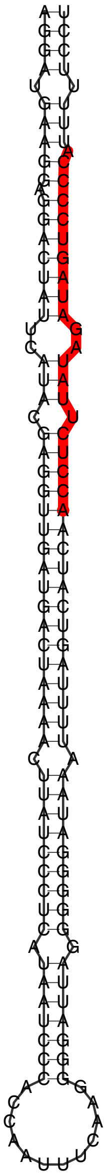

Name: miRC39

Sequence: ACCUCUUAUAGAUAGUCCCCA

Abundance: 12

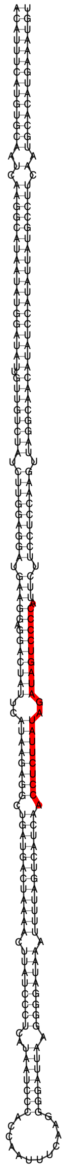

Name: miRC39

Sequence: ACCUCUUAUAGAUAGUCCCCA

Abundance: 12

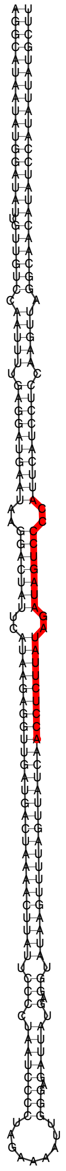

Name: miRC40

Sequence: AGACAGGUUCUUUUAUCUCAUG

Abundance: 12

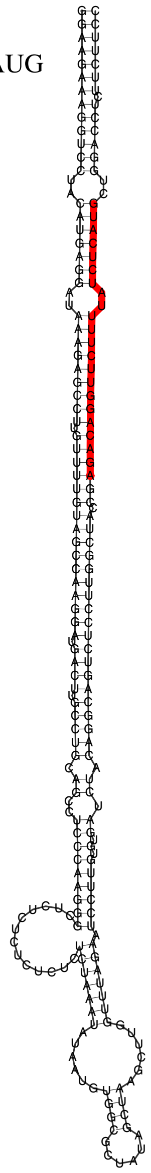

Name: miRC41

Sequence: UCGAUUUUAUGUUUUAAGUAU

Abundance: 11

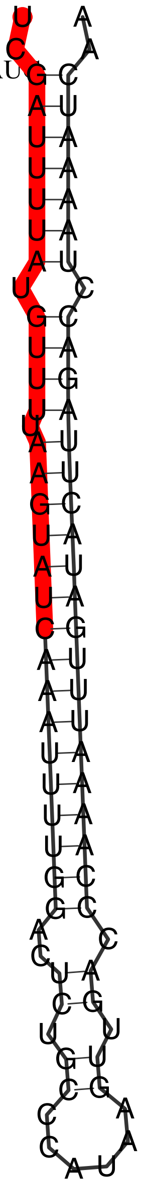

Name: miRC42

Sequence: UCUGACUUUUACCAGAAUCUGA

Abundance: 8

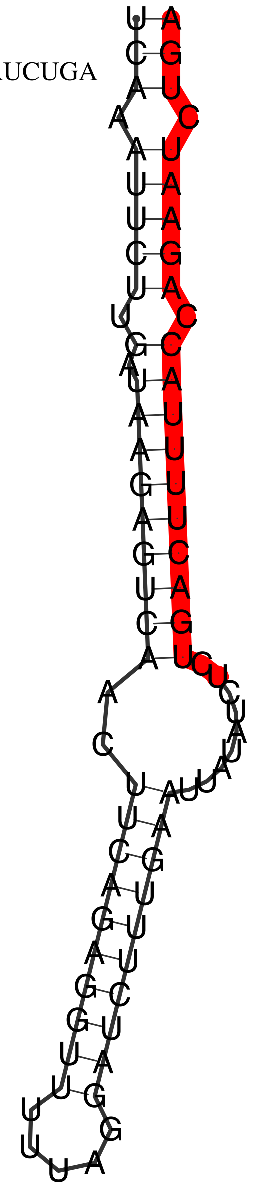

Name: miRC43

Sequence: CAUUAGAGCGGUGGUACACAA

Abundance: 7

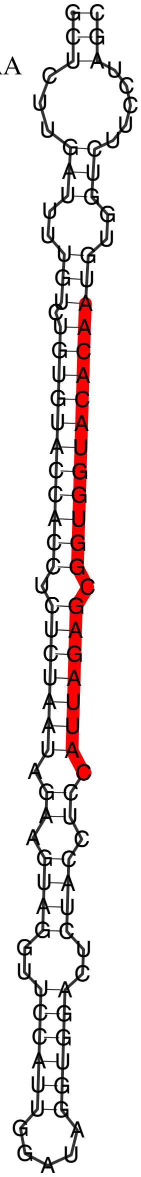

Name: miRC44

Sequence: UGCCAAGAAAGAGUUGCCCUA

Abundance: 5

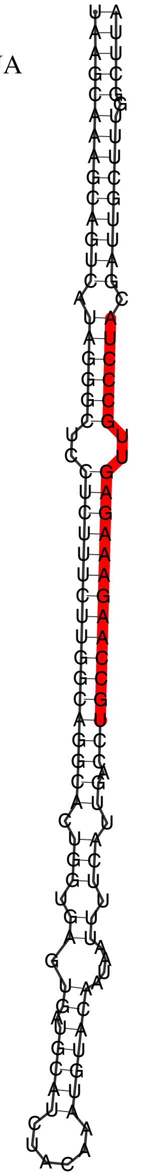

Name: miRC45

Sequence: ACCUCCUCAUUCUAACCCCUCA

Abundance: 4

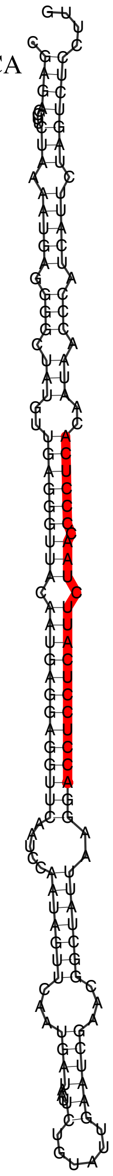

Name: miRC46

Sequence: UGCAUGCACCUUGAUAGAUGU

Abundance: 4

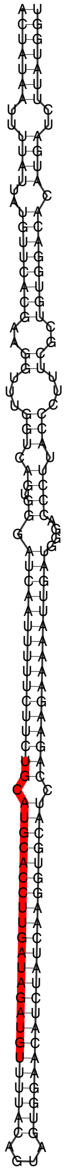

Supplement: Additional file 5 — Figure S1. Stem-loop structures for peach miRNAs. [file 1471-2229-12-149-S5.pdf]
