# Supplementary figures and images for: Unique expression, processing regulation, and regulatory network of peach (Prunus persica) miRNAs
Source: BMC Plant Biol. 2012 Aug 21;12:149. doi: 10.1186/1471-2229-12-149 (PMC3542160; doi:10.1186/1471-2229-12-149)

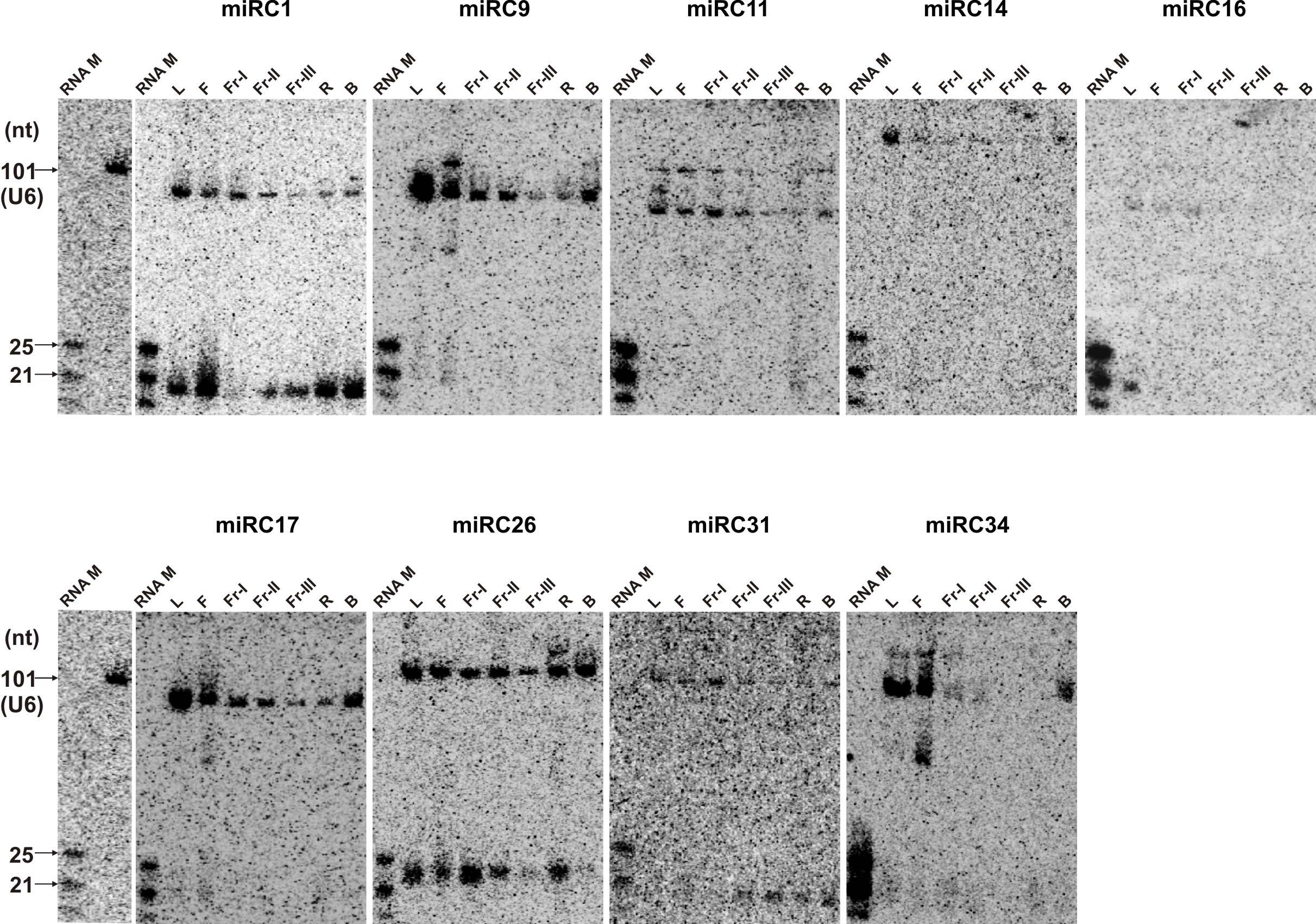

Supplement: Additional file 7 — Figure S2. Detection of pre-miRNAs in peach. [file 1471-2229-12-149-S7.jpeg]
